# Modified aryldifluorophenylsilicates with improved activity and selectivity in nucleophilic fluorination of secondary substrates†

**DOI:** 10.1039/d4ra04332d

**Published:** 2024-07-15

**Authors:** Michal Trojan, Adam Hroch, Evelin Gruden, Josef Cvačka, Jan Čejka, Gašper Tavčar, Markéta Rybáčková, Jaroslav Kvíčala

**Affiliations:** a Department of Organic Chemistry, University of Chemistry and Technology, Prague Technická 5 166 28 Prague 6 Czech Republic kvicalaj@vscht.cz; b Department of Inorganic Chemistry and Technology, “Jožef Stefan” Institute Jamova Cesta 39 Ljubljana Slovenia; c Institute of Organic Chemistry and Biochemistry of the Czech Academy of Sciences Flemingovo Náměstí 542/2 160 00 Prague 6 Czech Republic; d Department of Solid State Chemistry, University of Chemistry and Technology, Prague Technická 5 166 28 Prague 6 Czech Republic

## Abstract

Nucleophilic fluorination of secondary aliphatic substrates, especially of halides, still remains a challenge. Among the available reagents, TBAT belongs to one of the best choices due to its stability, affordable price and low toxicity. With the aim to improve its selectivity, we synthesized three analogues modified in the aryl part of the TBAT reagent with one or two electron donating methoxy groups or with one electron withdrawing trifluoromethyl group. All three reagents are air-stable compounds and their structure was confirmed by a single crystal X-ray analysis. In testing the reactivity and selectivity of the reagents with a library of secondary bromides, as well as of other selected primary and secondary substrates, we found that substitution with methoxy groups mostly improves both reactivity and selectivity compared to TBAT, while the substitution with trifluoromethyl group leads to inferior results. Difluorosilicates modified by more than two electron donating methoxy groups proved to be unstable and decomposed spontaneously to the HF_2_^−^ anion. DFT calculations of tetramethylammonium analogues of the studied reagents disclosed that the substitution of the phenyl group with the methoxy substituent lowers the transitions state energy of the decomposition to a fluorosilane–fluoride complex, while the substitution with the trifluoromethyl group has an opposite effect.

## Abstract 2

Difluorosilicates with MeO group(s) gave improved nucleophilic fluorination activity and selectivity of secondary substrates, while the CF_3_ group gave inferior results.

## Graphical abstract

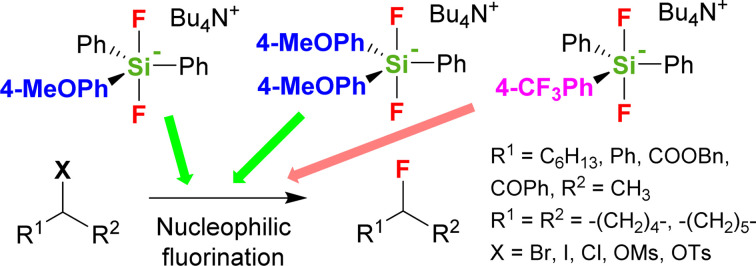

## Introduction

Organofluorine compounds find plentiful applications in medicinal chemistry,^1–3^ agrochemistry^4^ and industrial chemistry.^5^ Because radical or electrophilic fluorination requires the use of either highly corrosive fluorine gas or expensive electrophilic fluorinating reagents, nucleophilic fluorination still remains a basic tool for the synthesis of fluorinated substances.^6,7^ In analogy to other halogenations, either substitution of a hydroxy group forming *in situ* both leaving group and fluoride anion^8^ or fluorination of an appropriate leaving group such as sulfonate or halide group can be employed. For secondary aliphatic substrates, cheap reagents such as KF lead to preferential elimination due to its high basicity and poor solubility, secondary halides being most sensitive.^9^ Although it can be partially solved using crown ethers, cryptands or ionic liquids,^6,9^ especially in the combination with sterically hindered alcohols,^10–12^ these systems were not tested on secondary halides. Secondary bromide was successfully fluorinated by the combination of KF, sterically hindered alcohol and complex calixcrown ether.^13,14^ While the problem of solubility can be solved using quaternary ammonium cations, high basicity of TBAF (1) still remains the issue,^15,16^ which was partially solved by the use of sterically hindered hydrogen bond donors such as *tert*-butyl alcohol.^17^ The structure of KF–alcohol complexes was studied further both experimentally^17^ and theoretically.^18^ Unfortunately, no secondary halides were tested as substrates.

**Fig. 1:**
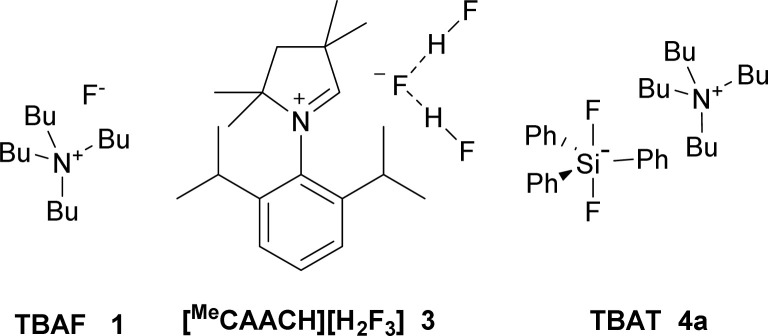
Reagents used for nucleophilic fluorination of 2-bromooctane (2a).

2-Bromooctane (2a) belongs to the substrates most prone to elimination and hence is often used as a benchmark for assignment of activity and selectivity of fluorinating reagents. The best results were reported to be achieved by a combination of tetrabutylammonium hydrogen difluoride in a 1 : 1 mixture with pyridine (75% substitution, 25% elimination), but no full experimental details are given.^19^ Second best reported yield was achieved using expensive AgF in combination with 2,2′-bipyridine (55%).^20^ Recently, 35% yield of fluorination was reported using a new class of reagents, stable dihydropyrrole NHC based dihydrogen trifluoride 3 (Fig. 1).^21^

One of the most promising, stable and commercially available fluorinating reagents, TBAT (4a), gave 2-fluorooctane with only 34% selectivity and high sixfold excess of reagent was used.^22^ We recently found that a twofold excess of TBAT is sufficient and that tetrabutylammonium cation prone to elimination can be substituted by other quaternary cations.^23^

**Scheme 1:**
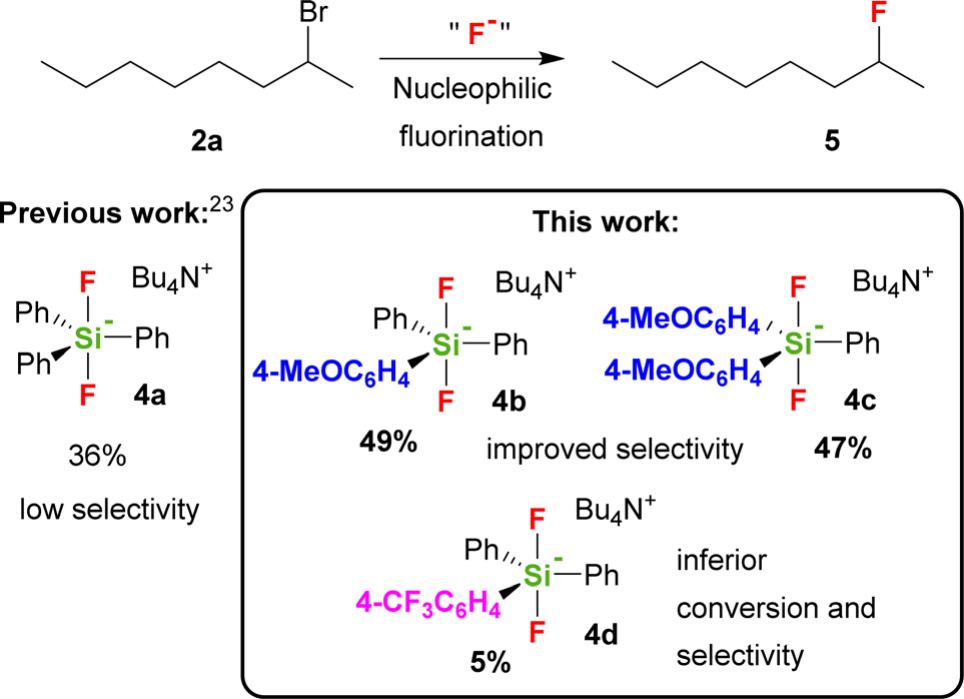
Nucleophilic fluorination with difluorosilicates.

The role of the presence of electron donating or electron withdrawing substituents on the activity of the TBAT analogue has never been studied with the exception of short remark by Mąkosza *et al.*, which synthesized tetrabutylammonium tris(4-chlorophenyl)difluorosilicate and tetrabutylammonium difluorotris(4-methylphenyl)silicate.^24^ They found that the latter reagent acts significantly more quickly than TBAT in fluorination of benzyl bromide.^25^ We hence wondered how the modification of the phenyl groups in TBAT (4a) with more strong electron donating or electron withdrawing groups will influence the activity and selectivity of the reagent not only with 2-bromooctane, but also with a larger series of secondary substrates. Our choice of possible substituents was limited by the synthetic approach including organometallic reagents, thus excluding stronger electron donating, *e.g.* amino groups, as well as stronger electron accepting, *e.g.* nitro or carbonyl groups. Using DFT methods, we also decided to study how this substitution will influence the PES (potential energy surface) of decomposition of difluorosilicates to fluorosilane–fluoride complexes as a first step of larger computational studies, targeted to understand better the mechanism of fluorinations with difluorosilicates. We found that modification of TBAT reagent with one or two electron donating methoxy groups (MeOTBAT, 4b, (MeO)_2_TBAT, 4c), improved the selectivity of fluorination for several secondary substrates including 2-bromooctane (2a), while the substitution with the electron withdrawing trifluoromethyl group led to inferior activity and selectivity (Scheme 1).

## Results and discussion

**Scheme 2:**
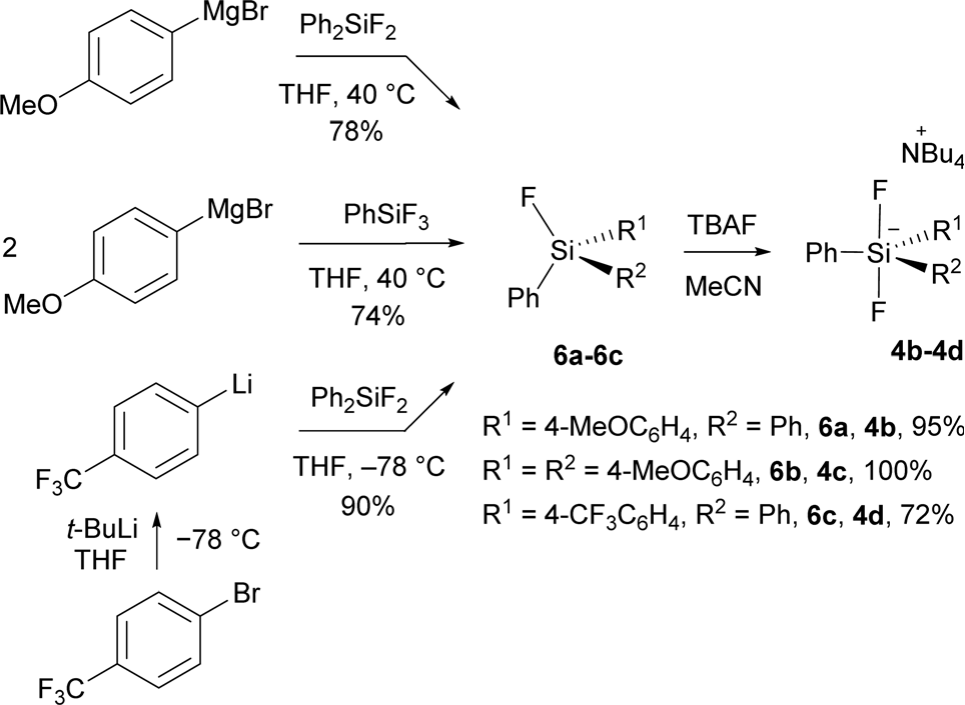
Preparation of new difluorosilicates 4b–4d.

New difluorosilicates 4b–4d were obtained by the reaction of the corresponding fluorosilanes 6a–6c with the solution of TBAF in THF. While fluorosilanes 6a, 6b containing one or two 4-methoxyphenyl groups were obtained by the reaction of commercial organomagnesium reagent with difluorodiphenylsilane or trifluorophenylsilane, fluorosilane 6c containing 4-(trifluoromethyl)phenyl group was formed using 4-(trifluoromethyl)phenyllithium, which was prepared from 1-bromo-4-(trifluoromethyl)benzene and *tert*-butyllithium (Scheme 2).

**Fig. 2:**
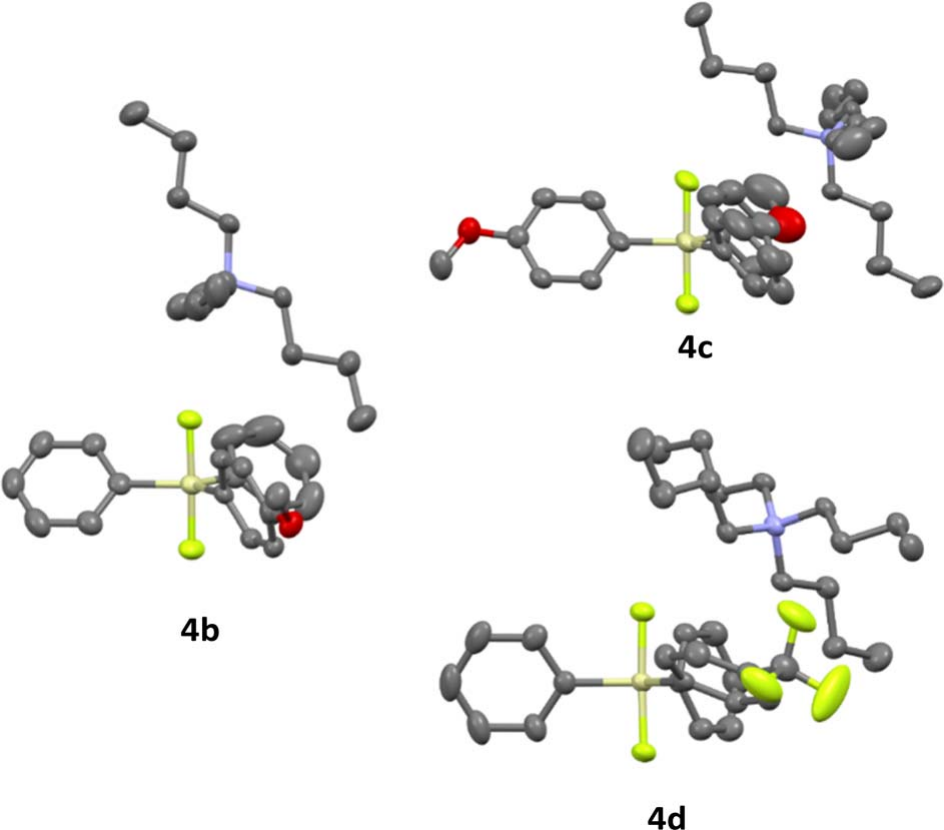
Single crystal structures of difluorosilicates 4b–4d.

Difluorosilicates 4b–4d are air stable compounds, their single crystal structures were obtained and are shown on Fig. 2. The details of crystallographic analyses are given in ESI.† The Si–F distances are quite identical for all three structures (172.6–172.7 pm for one SiF bond and 173.3–173.7 pm for the other), while the energy of the fluorosilane–fluoride complex probably does not depend much on the Si–F–N angle and hence this varies quite significantly from nearly perpendicular (107.6°) for 4c to nearly linear (162.3°) for 4b.

**Scheme 3:**
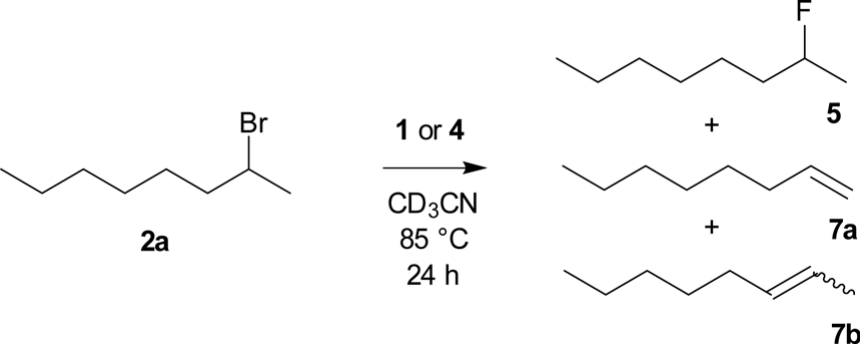
Fluorination of 2-bromooctane (2a) with TBAF (1) or difluorosilicates 4.

In the fluorination of 2-bromooctane, both difluorosilicates 4b, 4c modified with electron donating groups, gave better selectivities than both TBAF (1) and TBAT (4a) (Scheme 3, Table 1), while both the activity and selectivity of electron withdrawing CF_3_ group modified difluorosilicate 4d was significantly lower.

**Table 1:** Results of fluorination^a^ of 2-bromooctane (2a) with TBAF (1) or difluorosilicates 4

Entry	Reagent	Equiv.	Conversion	Product ratio 5 : 7b^b^ : 7a
1^d^	TBAF (1)^c^	2	99%	28 : 62 : 10
2^d^	TBAT (4a)	2	92%	39 : 53 : 8
3	MeOTBAT (4b)	2	96%	51 : 44 : 5
4	MeOTBAT (4b)	1	73%	36 : 58 : 6
5	(MeO)_2_TBAT (4c)	2	96%	49 : 45 : 6
6	CF_3_TBAT (4d)	2	78%	6 : 79 : 15

a CD_3_CN, 85 °C, 24 h, determined by ^1^H NMR.

b Mixture of (*E*)- and (*Z*)-isomers.

c TBAF solution in THF.

d From ref. 23.

In contrast to the previous research where high six fold excess has been employed,^22^ we in an analogy to our recent paper^23^ employed just two fold excess of the reagent and obtained nearly full conversion (Table 1 entry 3). On the other hand, the use of just one equivalent resulted in both inferior conversion and selectivity (Table 1 entry 4). Twofold excess of the reagents was thus used for all further fluorinations and products of elimination to *E*/*Z*-oct-2-ene 7b and oct-1-ene (7a) were observed as the only side products.

We also studied the role of the leaving group on the octan-2-yl moiety. The substitution/elimination ratio decreased in the order:–OMs > –Br > –I > –Cl.

**Fig. 3:**
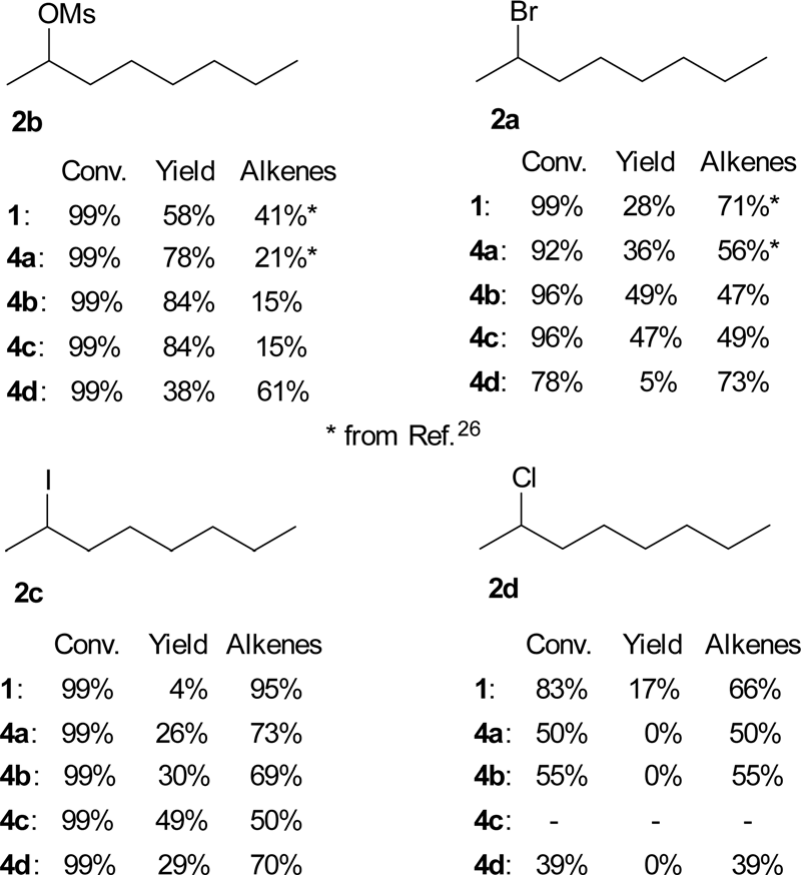
Conversions and NMR yields of fluorinations of various octan-2-yl substrates 2 (CD_3_CN, 85 °C, 24 h, determined by ^1^H NMR).

The results for TBAF (1) and both the known TBAT (4a) and new difluorosilicates 4b–4d are shown in Fig. 3. Comparison of 2-bromooctane (2a) with similar secondary substrates showed that, as expected, octan-2-yl mesylate (2b) gave better selectivity, where again commercial TBAT (4a) was surpassed by methoxylated TBAT analogues 4b, 4c with nearly total conversion and 84% and 84% respective NMR yield. Quite surprisingly and in agreement with ref. 22, 2-iodooctane (2c) gave more elimination than 2-bromooctane (2b). On the other hand, fluorination of 2-chlorooctane (2d) resulted in poor conversion and complete elimination to alkenes 7.

**Fig. 4:**
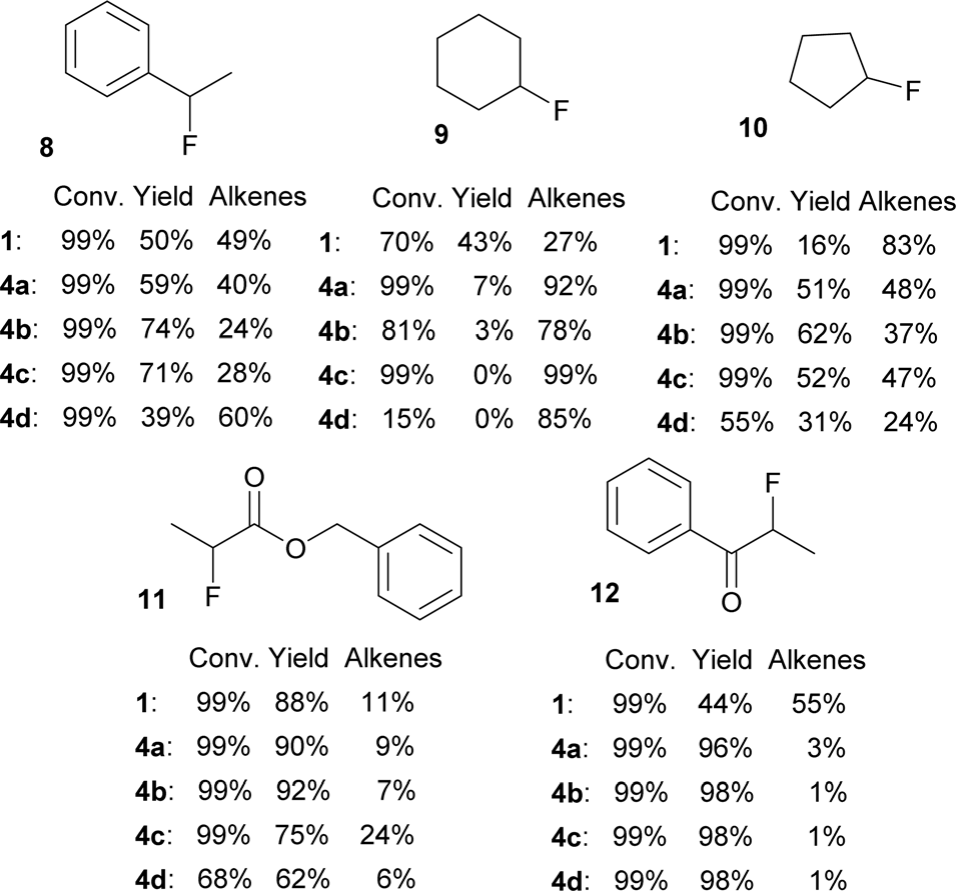
Conversions and NMR yields of fluorinations of secondary bromides with TBAF (1) or difluorosilicates 4 (CD_3_CN, 85 °C, 24 h, determined by ^1^H NMR).

A small set of secondary bromides was fluorinated with difluorosilicates 4 and the results are shown in Fig. 4. Fluorination of (1-bromoethyl)benzene (8) and bromocyclopentane (10) was in line with the previous results. Quite surprisingly, TBAF (1) gave the best results for fluorination of bromocyclohexane (9) and all difluorosilicates 4 failed to give acceptable results. On the other hand, secondary bromides with adjacent electron withdrawing groups (ester, ketone) gave very good yields of fluorination for all difluorosilicates 4, showing that they are not optimal substrates for testing purposes.

Regarding the poor results of fluorinations of bromide 9, we believe that steric hindrance can play here the major role together with high proneness to E2 elimination from the favorable cyclohexane ring conformation.

**Fig. 5:**
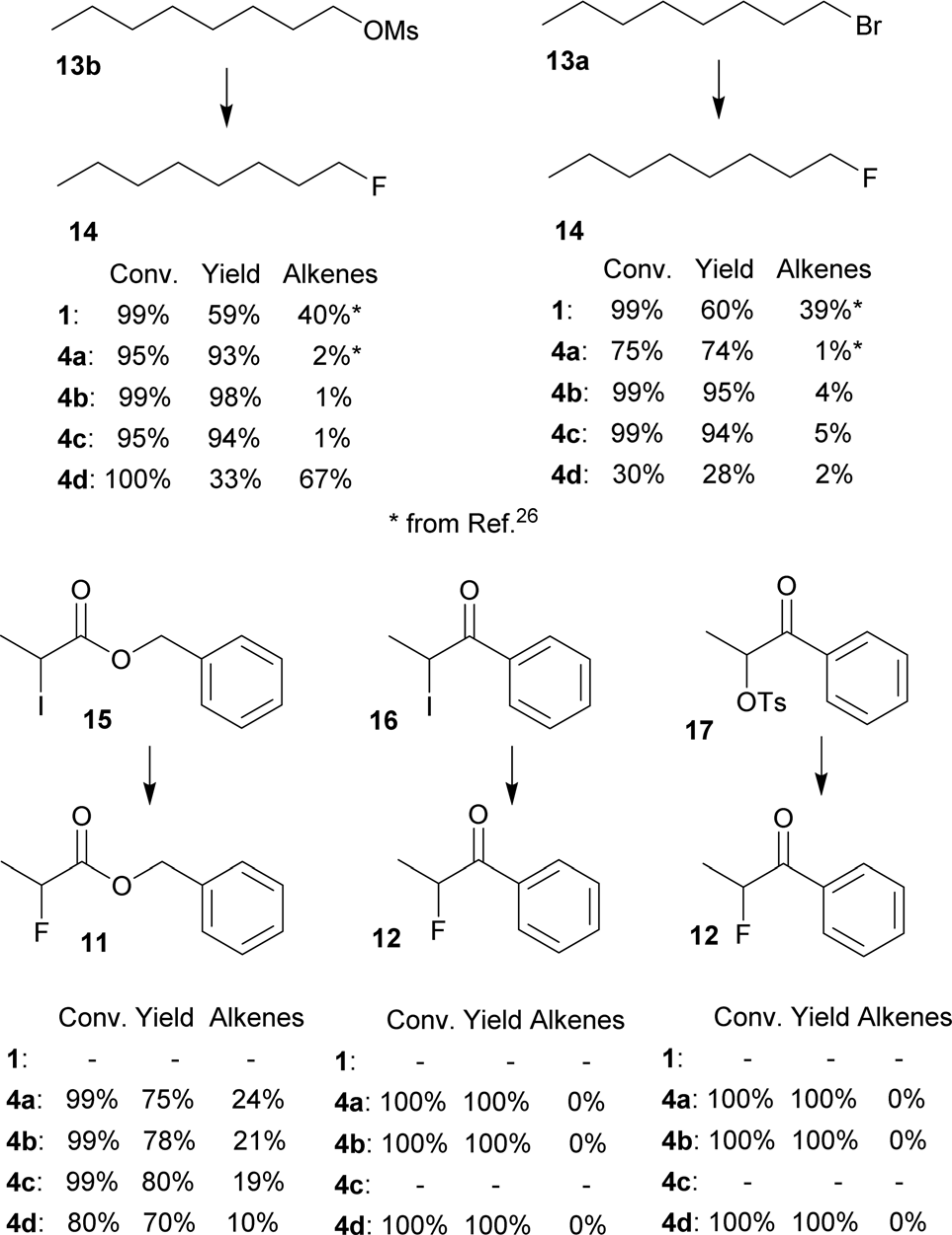
Conversions and NMR yields of fluorinations of other substrates (CD_3_CN, 85 °C, 24 h, determined by ^1^H NMR).

Furthermore, several primary and secondary substrates were also fluorinated for comparison in good to excellent yields (Fig. 5).

**Scheme 4:**
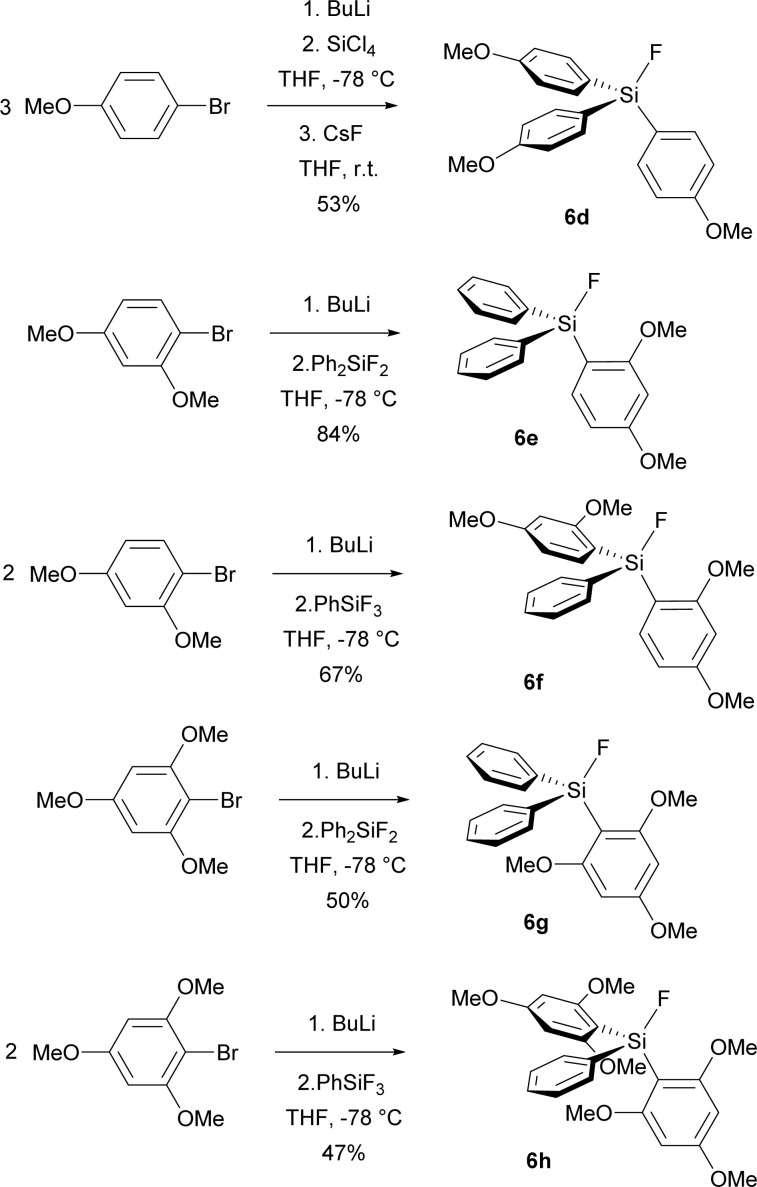
Preparation of fluorosilanes 6d–6h.

Encouraged by improved selectivity of methoxy group substituted analogues of TBAT, we synthesized starting fluorosilanes 6d–6g, containing even more methoxy groups. The synthesis employed similar strategy as the synthesis of fluorosilane 6c, namely bromine–lithium exchange by BuLi, followed by the reaction with the corresponding fluorophenylsilane or, in the case of fluorosilane 6d, with tetrachlorosilane followed by fluorination with CsF (Scheme 3). First purification was performed by vacuum distillation. In the case of fluorosilanes 6g, 6h bearing trimethoxyphenyl groups, lower conversion was caused by steric hindrance and the distilled products were contaminated by starting fluorophenylsilanes and other side-products. Fortunately, while trituration with a 7 : 1 hexane/dichloromethane mixture removed starting fluorophenylsilanes, pure hexane subsequently removed other organic side-products to give pure products 6g, 6h (Scheme 4).

**Scheme 5:**
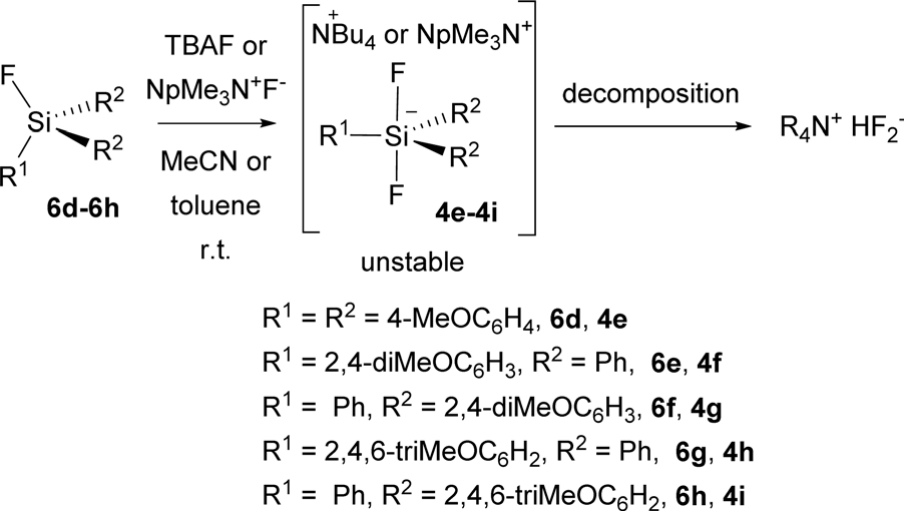
Attempted preparation of difluorosilicates 6d–6h.

Unfortunately, all attempts to prepare the corresponding difluorosilicates 4e–4i in an analogy to difluorosilicates 4a–4c resulted in the formation of unstable difluorosilicates or difluorosilicate/HF_2_^−^ mixtures, which slowly decomposed in an analogy to difluoromethyldiphenyl silicate anion, reported by us earlier,^23^ to HF_2_^−^ anion (see the spectra in ESI†) (Scheme 5).

Thus, difluorosilicate 4d probably represents the borderline stable structure. We further attempted to prevent possible elimination of HF from Bu_4_N^+^ cation, substituting it for neopentyltrimethyl-ammonium (NpMe_3_N^+^) cation and changing the solvent for toluene, but with the same outcome (see the spectrum in ESI†). The source of the proton to form HF_2_^−^ anion from unstable difluorosilicates 6d–6h is unknown to us especially in the latter case, both starting fluorosilanes and neopentyltrimethyl-ammonium fluoride were dried for several days under high vacuum. This will be the aim of our further studies.

### Computations

Finally, we wondered how substitution with electron donating methoxy group or electron withdrawing trifluoromethyl group will influence the geometry of aryldifluorodiphenylsilicates and how it will influence the activation energy of their decomposition to fluorosilane–fluoride complexes. Recently, the mechanism of the decomposition of TBAT to fluorosilane and TBAF was studied by a variety of NMR methods in THF and MeCN and found to be 72.5 kJ mol^−1^ at 300 K in THF and probably lower in MeCN.^26^ We hence started a DFT study of these compounds. This is the first part of a larger computational study related to the mechanism of nucleophilic fluorination with difluorosilicates, which is yet unknown. In more detail, it is not known whether direct transfer of fluorine (and which of the two fluorine atoms) to the substrate proceeds, or whether this is a two step process with first decomposition to fluorosilane and fluoride, followed by fluorination with naked fluoride anion. For simplicity, we substituted tetrabutylammonium cation with simpler tetramethyl ammonium. While preliminary calculations were performed using Gaussian 16 program suite,^27^ quantitative results were obtained with the ORCA computational program.^28^ Full details and discussion of the minimal geometries are given in ESI.†

**Fig. 6:**
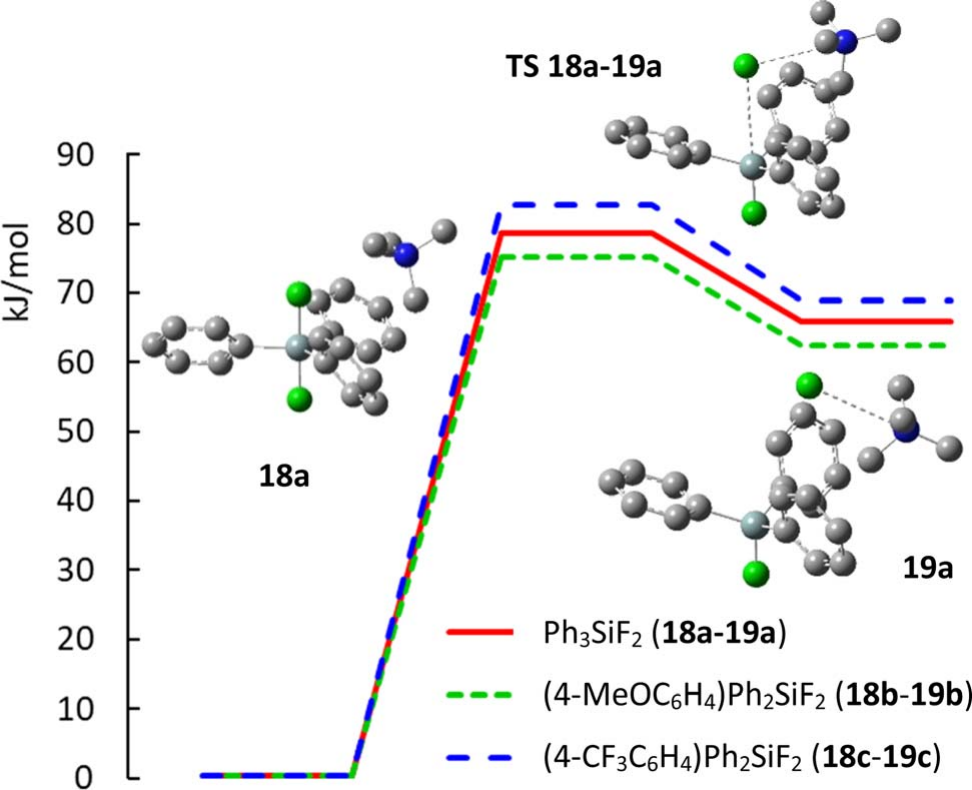
Potential energy curve of decomposition of difluorosilicates 18a–18c to fluorosilane–fluoride complexes 19a–19c.

The computed structures of difluorosilicate 18a containing three phenyl groups, difluorosilicate 18b modified with the methoxygroup and difluorosilicate 18c with the trifluoromethyl group agree well with the obtained single crystal structures, the error in the Si–F lengths not exceeding 1 pm. On the other hand, the Si–F–N angle differs significantly from the crystal structures, because this is given mostly by crystal packing. Compared to difluorotriphenylsilicate (18a), the presence of the electron-donating methoxygroup in the aryl in 18b results in lowering of both the transition state and fluorosilane–fluoride complex 19b energies by about 4 kJ mol^−1^, while the presence of the electron-accepting group in 18c has just the opposite effect (19c) (see Fig. 6 for the key structures and saddle point energies).

To bring some rationale to the experimental results, analysis of the computed structures shows that the presence of electron-donating methoxy group on the phenyl ring decreases the positive hyperconjugation from the Si–F bonds, resulting in smaller Mulliken charge on silicon and longer Si–F bond (−0.327 and 1.741 Å, respectively) compared to unsubstituted phenyl (−0.351 and 1.740 Å, respectively). On the other hand, the presence of electron-accepting trifluoromethyl group on the ring results in higher positive hyperconjugation from the Si–F bonds, resulting in higher Mulliken charge on silicon and shorter Si–F bond (−0.602 and 1.733 Å). Correspondingly, longer Si–F bonds in the silicate imply higher nucleophilic reactivity with a softer reagent and shorter Si–F bonds lower nucleophilic reactivity and enhanced elimination due to a harder reagent.

## Experimental

### Materials and methods

All reactions were performed under an argon atmosphere in oven dried flasks using standard inert technique, unless otherwise noted. Fluorinations were performed in sealed vials. ^1^H NMR spectra were recorded with Agilent 400-MR DDR2 spectrometer at working frequencies 399.94 MHz for ^1^H NMR, 376.29 MHz for ^19^F NMR and 100.58 MHz for ^13^C NMR or with JEOL-ECZL400G spectrometer at working frequencies 399.78 MHz for ^1^H NMR, 376.17 MHz for ^19^F NMR and 100.53 MHz for ^13^C NMR, in deuterated solvents. Chemical shifts (*δ*) are reported in parts per million (ppm) with reference to the residual solvent peak. Signals are described as s = singlet, d = doublet, t = triplet, m = multiplet, bs – broad singlet.

Coupling constants (*J*) are reported in Hz. Mass spectra were recorded on LTW Orbitrap XL (Thermo Fisher Scientific) instrument. Difluorodiphenylsilane and 2-bromooctane (2a, containing 10% of 3-bromooctane as impurity) was purchased from Fluorochem, (1-bromoethyl)benzene, bromocyclohexane and bromocyclo-pentane were purchased from Merck. Octyl mesylate (13b),^29^ octan-2-yl mesylate (2b),^30^ 2-iodooctane (2c),^31^ 2-chlorooctane (2d),^32^ benzyl 2-bromopropionate,^33^ benzyl 2-iodopropionate (15),^33^ 2-bromo-1-phenylpropan-1-one,^34^ 2-iodo-1-phenylpropan-1-one (16),^35^ 1-oxo-1-phenylpropan-2-yl tosylate (17),^36^ phenyltrifluorosilane,^37^ 1-bromo-2,4-dimethoxybenzene^38,39^ and 2-bromo-1,3,5-trimethoxybenzene^39^ were prepared according to the published procedures.

### Preparation of silanes 6

#### Fluoro(4-methoxyphenyl)diphenylsilane (6a)

To a Ph_2_SiF_2_ solution (0.58 g; 2.6 mmol) in an anhydrous THF (3 mL), a solution of 4-methoxyphenylmagnesium bromide (0.5 M in THF; 5.3 mL; 2.6 mmol) was added and the mixture was stirred for 8 h at 40 °C. The solvent was then evaporated and the mixture was purified by column chromatography (hexane/EtOAc) to give fluorosilane 6a (0.63 g, 78%). ^1^H NMR (399.94 MHz, CDCl_3_):^40^*δ* 3.82 (s, C**H**_3_O, 3H); 6.89–7.00 (m (app. d, ^3^*J*_H–H_ = 8.7 Hz), ^4-MeO^C**H**, 2H); 7.40 (m, ^Ar^C**H**, 4H); 7.45–7.50 (m, ^Ar^C**H**, 2H); 7.54–7.57 (m (app. d, ^3^*J*_H–H_ = 8.7 Hz), ^4-MeO^C**H**, 2H); 7.61–7.65 (m, ^Ar^C**H**, 4H) ppm. ^13^C NMR (100.58 MHz, CDCl_3_): *δ* 55.2 (**C**H_3_O, 1C); 114.0 (^4-MeO-*meta*^**C**H, 2C); 123.4 (d, ^3^*J*_C–F_ = 17.6 Hz, ^4-MeO-*ipso*^**C**–Si, 1C); 128.2 (^*meta*^**C**H, 4C); 130.9 (^*para*^**C**H, 2C); 133.0 (d, ^3^*J*_C–F_ = 17.5 Hz, ^*ipso*^**C**–Si, 2C); 135.1 (d, ^4^*J*_C–F_ = 1.8 Hz, ^*ortho*^**C**H, 4C); 136.9 (d, ^4^*J*_C–F_ = 1.5 Hz, ^4-MeO-*ortho*^**C**H, 2C); 161.9 (^4-MeO-*para*^**C**, 1C) ppm. ^19^F NMR (376.29 MHz, CDCl_3_):^2^*δ* −168.50 (s, 1F) ppm. HRMS (EI): calcd for C_19_H_17_FOSi [M]^+^ 308.1027, found 308.1030.

#### Fluorobis(4-methoxyphenyl)(phenyl)silane (6b)

A solution of 4-methoxyphenylmagnesium bromide (0.5 M in THF; 25.9 mL; 13 mmol) was added to a solution of PhSiF_3_ (1.0 g; 6.5 mmol) in an anhydrous THF (10 mL) and the mixture was stirred for 8 h at 40 °C. The solvent was then evaporated and the crude product was purified by column chromatography (CH_2_Cl_2_) to give fluorosilane 6b (1.6 g, 74%). ^1^H NMR (399.94 MHz, CDCl_3_): *δ* 3.83 (s, C**H**_3_O, 6H); 6.95 (m (app. d, ^3^*J*_H–H_ = 8.7 Hz), ^4-MeO-*ortho*^C**H**, 4H); 7.39–7.43 (m, ^*meta*-Ph^C**H**, 2H); 7.45–7.50 (m, ^*para*-Ph^C**H**, 1H); 7.54–7.58 (m (app. d, ^3^*J*_H–H_ = 8.7 Hz), ^4-MeO-*meta*^C**H**, 4H); 7.62–7.65 (d, ^3^*J*_H–H_ = 8.7 Hz, ^*ortho*-Ph^C**H**, 2H) ppm. ^13^C NMR (100.58 MHz, CDCl_3_): *δ* 55.2 (**C**H_3_O, 2C); 114.0 (^4-MeO-*meta*^**C**H, 4C); 123.9 (d, ^3^*J*_C–F_ = 18.0 Hz, ^4-MeO-*ipso*^**C**–Si, 2C); 128.1 (^*meta*^**C**H, 2C); 130.7 (^*para*^**C**H, 1C); 133.4 (d, ^3^*J*_C–F_ = 17.8 Hz, ^*ipso*^**C**–Si, 1C); 135.0 (d, ^4^*J*_C–F_ = 1.7 Hz, ^*ortho*^**C**H, 2C); 136.9 (d, ^4^*J*_C–F_ = 1.5 Hz, ^4-MeO-*ortho*^**C**H, 4C); 161.8 C (^4-MeO-*para*^**C**, 2C) ppm. ^19^F NMR (376.29 MHz, CDCl_3_): *δ* −167.84 (s, 1F) ppm. HRMS (EI): calcd for C_20_H_19_FO_2_Si [M]^+^ 338.1133, found 338.1135.

#### Fluorodiphenyl[4-(trifluoromethyl)phenyl]silane (6c)

A solution of 1-bromo-4-(trifluoromethyl)benzene (1.09 g; 4.84 mmol) in an anhydrous THF (5 mL) was added to a solution of *tert*-butyllithium (1.4 M solution in pentane; 7.5 mL; 10 mmol) in an anhydrous THF (10 mL) at −78 °C and the mixture was stirred for 15 min at −78 °C. The reaction mixture was then transferred through a capillary into a solution of difluorodiphenylsilane (2.341 g; 10.61 mmol) in an anhydrous THF (10 mL) at −78 °C. Reaction was stirred at −78 °C for 3 h and left to warm to r.t. overnight. After filtration through a syringe filter, the solvent was evaporated. Crude product was purified by vacuum distillation (104–115 °C/9 Pa) yielding silane 6c (1.47 g, 88%) containing about 20% of *tert*-butylfluorodiphenyl-silane. ^1^H NMR (399.78 MHz, CDCl_3_): *δ* 7.41–7.47 (m, ^*ortho*^C**H**, 4H); 7.49–7.55 (m, ^*para*^C**H**, 2H); 7.61–7.65 (m, ^*meta*^C**H**, 4H); 7.65–7.69 (m (app. d, ^3^*J*_H–H_ = 8.0 Hz), ^4-CF_3_^C**H**, 2H); 7.75–7.80 (m, (app. d, ^3^*J*_H–H_ = 8.0 Hz), ^4-CF_3_^C**H**, 2H), ppm. ^13^C NMR (100.53 MHz, CDCl_3_): *δ* 124.0 (q, ^1^*J*_C–F_ = 272 Hz, **C**F_3_, 1C); 124.8 (q, ^3^*J*_C–F_ = 3.8 Hz, ^4-CF_3_-*meta*^**C**, 1C); 128.4 (^*meta*^**C**H, 4C); 131.4 (^*para*^CCH, 2C); 131.5 (d, ^3^*J*_C–F_ = 16.7 Hz, ^*ipso*^**C**–Si, 2C); 132.2 (q, ^2^*J*_C–F_ = 32.5 Hz, ^4-CF_3_-*para*^**C**, 1C); 135.1 (d, ^4^*J*_C–F_ = 1.7 Hz, ^*ortho*^**C**H, 4C); 135.4 (d, ^4^*J*_C–F_ = 1.9 Hz, ^*ortho*^**C**H, 2C); 137.5 (d, ^3^*J*_C–F_ = 17.1 Hz, ^4-CF_3_-*ipso*^**C**–Si, 1C) ppm. ^19^F NMR (376.17 MHz, CDCl_3_): *δ* −63.64 (s, C**F**_3_, 3F); −170.35 (s, Si**F**, 1F) ppm. HRMS (ESI): calcd for C_19_H_14_F_4_Si [M]^+^ 346.0795, found 346.0797.

#### Fluorotris(4-methoxyphenyl)silane (6d)

A flask was charged with a solution of 1-bromo-4-methoxybenzene (4.49 g; 24.0 mmol) in an anhydrous THF (25 mL) and cooled to −78 °C. Another flask was charged with anhydrous THF (25 mL) and a solution of BuLi (2.45 M in hexanes; 10 mL, 24.5 mmol). The content of this flask was transferred to the first flask and the mixture was left to stir for 30 min at −78 °C. In the third flask, a solution of SiCl_4_ (1.30 g, 7.70 mmol) in an anhydrous THF was cooled to −78 °C, the content of the first flask was transferred to it and the mixture was left to stir at −78 °C for 2 h. The mixture was left to stir and warm to r.t. overnight, filtered and transferred to the flask, which was before charged in glove box with anhydrous CsF (2.24 g, 14.7 mmol). The mixture was left to stir overnight, solids were filtered off and the solvent were evaporated. Final purification by vacuum distillation (205–215 °C/30 Pa) afforded silane 6d (1.49 g, 53%). ^1^H NMR (399.94 MHz, CDCl_3_): *δ* 3.84 (s, C**H**_3_O, 9H); 6.96 (m (app. d, ^3^*J*_H–H_ = 8.5 Hz), ^*meta*-Ar^C**H**, 6H); 7.57 (m (app. d, ^3^*J*_H–H_ = 8.5 Hz), ^*ortho*-Ar^C**H**, 6H) ppm. ^13^C NMR (100.53 MHz, CDCl_3_): *δ* 55.2 (O**C**H_3_, 3C); 113.9 (^*meta*-Ar^**C**H, 6C); 124.3 (d, ^3^*J*_C–F_ = 18.4 Hz, ^*ipso*-Ar^**C**Si, 3C); 136.9 (^*ortho*-Ar^**C**H, 6C); 161.7 (3C, COCH_3_) ppm. ^19^F NMR (376.17 MHz, CDCl_3_): *δ* −166.67 (s, 1F) ppm. HRMS (EI): calcd for C_21_H_21_FO_3_Si 368.1239, found 368.1245.

#### (2,4-Dimethoxyphenyl)(fluoro)(diphenyl)silane (6e)

A flask was charged with a solution of 1-bromo-2,4-dimethoxybenzene (1.35 g; 6.23 mmol) in an anhydrous THF (8 mL) and cooled to −78 °C. Another flask was charged with anhydrous THF (8 mL) and a solution of BuLi (2.45 M in hexanes; 2.6 mL, 6.4 mmol). The content of this flask was transferred to the first flask and the mixture was left to stir for 30 min at −78 °C. In the third flask, a solution of difluorodiphenylsilane (1.54 g, 6.97 mmol) in an anhydrous THF (10 mL) was cooled to −78 °C and the content of the first flask was added to it over 10 min. The mixture was then stirred at −78 °C for 3 h and left to warm overnight while stirred. The solvents were evaporated to a half volume of the mixture, which was then filtered through a syringe filter, and remaining solvents were evaporated to dryness. Final purification by vacuum distillation (170–175 °C/40 Pa) gave silane 6e (1.78 g, 84%) as a viscous liquid, which solidified over a week into a waxy solid. ^1^H NMR (399.78 MHz, CDCl_3_): *δ* 3.66 (s, ^*ortho*^OC**H**_3_, 6H); 3.85 (s, ^*para*^OC**H**_3_, 6H); 6.48 (d, ^4^*J*_H–H_ = 2.0 Hz, ^*meta*-Ar^C**H**, 1H); 6.58 (dd, ^3^*J*_H–H_ = 8.2 Hz, ^4^*J*_H–H_ = 2.0 Hz, ^*meta*-Ar^C**H**, 1H); 7.37–7.42 (m, ^*meta*-Ph^C**H**, 4H); 7.44–7.49 (m, ^*para*-Ph^C**H**, 2H); 7.49 (d, ^3^*J*_H–H_ = 8.2 Hz, ^*ortho*-Ar^C**H**, 1H); 7.69 (dd, ^3^*J*_H–H_ = 8.0 Hz, ^4^*J*_H–H_ = 1.5 Hz, ^*ortho*-Ph^C**H**, 4H) ppm. ^13^C NMR (100.53 MHz, CDCl_3_): *δ* 55.3 (^*ortho*^O**C**H_3_, 1C); 55.4 (^*para*^O**C**H_3_, 1C); 98.2 (^*meta*-Ar^**C**H, 1C); 105.4 (^*meta*-Ar^**C**H, 1C); 112.48 (d, ^3^*J*_C–F_ = 17.0 Hz, ^*ipso*-Ar^**C**Si, 1C); 127.9 (^*meta*-Ph^**C**H, 4C); 130.4 (^*para*-Ph^**C**H, 2C); 133.9 (d, ^3^*J*_C–F_ = 17.0 Hz, ^*ipso*-Ph^**C**Si, 2C); 134.9 (d, ^4^*J*_C–F_ = 2.0 Hz, ^*ortho*-Ph^**C**H, 4C); 137.7 (d, ^4^*J*_C–F_ = 2.7 Hz, ^*ortho*-Ar^**C**H, 1C); 164.2 (^*para*-Ar^**C**OCH_3_, 1C); 166.2 (d, ^4^*J*_C–F_ = 2.3 Hz, ^*ortho*-^**ArCOCH3**, 1C) ppm. ^19^F NMR (376.17 MHz, CDCl_3_): *δ* −171.00 (s, Si**F**, 1F) ppm. HRMS (EI): calcd for C_20_H_19_FO_2_Si 338.1133, found 338.1130.

#### Bis(2,4-dimethoxyphenyl)(fluoro)(phenyl)silane (6f)

A flask was charged with a solution of 1-bromo-2,4-dimethoxybenzene (3.99 g; 18.4 mmol) in an anhydrous THF (19 mL) and cooled to −78 °C. Another flask was charged with anhydrous THF (19 mL) and a solution of BuLi (2.45 M in hexanes; 7.5 mL, 18.4 mmol). The content of this flask was transferred to the first flask and the mixture was left to stir for 15 min at −78 °C. In the third flask, a solution of trifluorophenylsilane (1.49 g, 9.18 mmol) in an anhydrous THF (19 mL) was cooled to −78 °C and the content of the first flask was added to it over 10 min. The mixture was then stirred at −78 °C for 3 h and left to warm overnight while stirred. The solvents were evaporated to a half volume of the mixture, which was then filtered through a syringe filter, and remaining solvents were evaporated to dryness. Final purification by vacuum distillation (205–210 °C/10 Pa) gave silane 6f (2.47 g, 67%) as a viscous liquid, which solidified over two days into a waxy solid. ^1^H NMR (399.78 MHz, CDCl_3_): *δ* 3.64 (s, ^*ortho*^OC**H**_3_, 6H); 3.83 (s, ^*para*^OC**H**_3_, 6H); 6.45 (d, ^4^*J*_H–H_ = 2.0 Hz, ^*meta*-Ar^C**H**, 2H); 6.51 (dd, ^3^*J*_H–H_ = 8.2 Hz, ^4^*J*_H–H_ = 2.0 Hz, ^*meta*-Ar^C**H**, 2H); 7.33 (d, ^3^*J*_H–H_ = 8.2 Hz, ^*ortho*-Ar^C**H**, 2H); 7.36 (m, ^*para*-Ph^C**H**, 1H); 7.66 (dd, ^3^*J*_H–H_ = 8.2 Hz, ^3^*J*_H–H_ = 2.1 Hz, ^*ortho*-Ph^C**H**, 2H) ppm. ^13^C NMR (100.53 MHz, CDCl_3_): *δ* 55.4 (^*ortho*^O**C**H_3_, 2C); 55.4 (^*para*^O**C**H_3_, 2C); 98.1 (^*meta*-^**ArCH**, 2C); 105.1 (^*meta*-**Ar**^**C**H, 2C); 113.51 (d, ^3^*J*_C–F_ = 16.7 Hz, ^*ipso*-Ar^**C**Si, 2C); 127.6 (^*meta*-Ph^**C**H, 2C); 129.8 (^*para*-Ph^**C**H, 1C); 134.6 (d, ^4^*J*_C–F_ = 2.0 Hz, ^*ortho*-Ph^**C**H, 2C); 135.2 (d, ^3^*J*_C–F_ = 17.8 Hz, ^*ipso*-Ph^**C**Si, 1C); 138.1 (d, ^4^*J*_C–F_ = 3.0 Hz, ^*ortho*-Ar^**C**H, 2C); 163.7 (^*para*-Ar^**C**OCH_3_, 2C); 166.3 (^*ortho*-Ar^**C**OCH_3_, 2C) ppm. ^19^F NMR (376.17 MHz, CDCl_3_): *δ* −169.46 (s, Si**F**, 1F) ppm. HRMS (EI): calcd for C_22_H_23_FO_4_Si 398.1344, found 398.1355.

#### (Fluoro)(diphenyl)(2,4,6-trimethoxyphenyl)silane (6g)

A flask was charged with a solution of 2-bromo-1,3,5-dimethoxybenzene (1.09 g; 4.41 mmol) in an anhydrous THF (10 mL) and cooled to −78 °C. Another flask was charged with anhydrous THF (10 mL) and a solution of BuLi (2.45 M in hexanes; 1.8 mL, 4.41 mmol). The content of this flask was transferred to the first flask and the mixture was left to stir for 30 min at −78 °C. In the third flask, a solution of difluorodiphenylsilane (0.98 g, 4.45 mmol) in an anhydrous THF (10 mL) was cooled to −78 °C and the content of the first flask was added to it. The mixture was then stirred at −78 °C for 3 h and left to warm overnight while stirred. The solvents were evaporated to a half volume of the mixture, which was then filtered through a syringe filter, and remaining solvents were evaporated to dryness. A crude product was transferred to a vial, hexane/dichloromethane 7 : 1 mixture (2 mL) was added to it and the mixture was left for 5 min in an ultrasound bath. After separation of the supernatant, silane 6g was obtained after final drying *in vacuo* (0.83 g, 50%). ^1^H NMR (399.78 MHz, CDCl_3_): *δ* 3.57 (s, ^*ortho*^OC**H**_3_, 6H); 3.84 (s, ^*para*^OC_3_, 3H); 6.11 (s, ^*meta*-^**ArCH**, 2H); 7.33–7.34 (m, ^*meta*+*para*-Ph^C**H**, 6H); 7.64–7.67 (m, ^*ortho*-Ph^C**H**, 4H) ppm. ^13^C NMR (100.53 MHz, CDCl_3_): *δ* 55.4 (^*para*^O**C**H_3_, 1C); 55.5 (^*ortho*^O**C**H_3_, 2C); 91.0 (^*meta*-Ar^**C**H, 2C); 99.6 (d, ^3^*J*_C–F_ = 14.5 Hz, ^*ipso*-Ar^**C**Si, 1C); 127.6 (^*meta*-Ph^**C**H, 4C); 129.9 (^*para*-Ph^**C**H, 2C); 134.7 (d, ^4^*J*_C–F_ = 2.0 Hz, ^*ortho*-Ph^**C**H, 4C); 135.6 (d, ^3^*J*_C–F_ = 17.6 Hz, ^*ipso*-Ph^**C**Si, 2C); 165.04 (^*para*-Ar^**C**OCH_3_, 1C); 167.25 (^*ortho*-Ar^**C**OCH_3_, 2C) ppm. ^19^F NMR (376.17 MHz, CDCl_3_): *δ* −162.74 (s, Si**F**, 1F) ppm. HRMS (EI): calcd for C_21_H_21_FO_3_Si 368.1239, found 368.1244.

#### (Fluoro)(phenyl)bis(2,4,6-trimethoxyphenyl)silane (6h)

A flask was charged with a solution of 2-bromo-1,3,5-dimethoxybenzene (2.53 g; 10.02 mmol) in an anhydrous THF (30 mL) and cooled to −78 °C. Another flask was charged with anhydrous THF (10 mL) and a solution of BuLi (2.45 M in hexanes; 4.4 mL, 11 mmol). The content of this flask was transferred to the first flask and the mixture was left to stir for 30 min at −78 °C. In the third flask, a solution of fluorotriphenylsilane (0.864 g, 5.33 mmol) in an anhydrous THF (15 mL) was cooled to −78 °C and the content of the first flask was added to it. The mixture was then stirred at −78 °C for 3 h and left to warm overnight while stirred. The solvents were evaporated to a half volume of the mixture, which was then filtered through a syringe filter, and remaining solvents were evaporated to dryness. To a crude product hexane/dichloromethane 7 : 1 mixture (5 mL) was added to it and the mixture was left for 3 min in an ultrasound bath. After separation of the supernatant, hexane (5 mL) was added to the mixture and it was again left for 5 min in an ultrasound bath. After separation of the supernatant, silane 6h was obtained after final drying *in vacuo* (1.10 g, 47%). ^1^H NMR (399.78 MHz, CDCl_3_): *δ* 3.50 (s, ^*ortho*^OC**H**_3_, 12H); 3.81 (s, ^*para*^OC**H**_3_, 6H); 6.06 (s, ^*meta*-Ar^C**H**, 4H); 7.26–7.32 (m, ^*meta*+*para*-Ph^C**H**, 3H); 7.62–7.66 (m, ^*ortho*-^**Ph**C**H**, 2H) ppm. ^13^C NMR (100.53 MHz, CDCl_3_): *δ* 55.3 (^*para*^O**C**H_3_, 2C); 55.7 (^*ortho*^O**C**H_3_, 4C); 91.1 (^*meta*-Ar^**C**H, 4C); 104.1 (d, ^3^*J*_C–F_ = 16.0 Hz, ^*ipso*-Ar^**C**Si, 2C); 126.9 (^*meta*-Ph^**C**H, 2C); 128.6 (^*para*-Ph^**C**H, 1C); 133.9 (d, ^4^*J*_C–F_ = 2.4 Hz, ^*ortho*-Ph^**C**H, 2C); 139.1 (d, ^3^*J*_C–F_ = 18.3 Hz, ^*ipso*-Ph^**C**Si, 1C); 163.9 (^*para*-Ar^**C**OCH_3_, 2C); 166.78 (^*ortho*-Ar^**C**OCH_3_, 4C) ppm. ^19^F NMR (376.17 MHz, CDCl_3_): *δ* −161.18 (s, Si**F**, 1F) ppm. HRMS (EI): calcd for C_24_H_27_FO_6_Si 458.1555, found 458.1556.

### Preparation of difluorosilicates 4

#### Tetrabutylammonium 4-methoxyphenyldiphenyldifluorosilicate (4b)

A solution of TBAF (1 M solution in THF; 1.1 mL; 1.1 mmol) was added to a solution of 6a (0.35 g; 1.1 mmol) in dry MeCN (2 mL). Reaction mixture was stirred for 1 h at r.t. After evaporation of the solvent, solid product was formed. Silicate 4b was obtained after drying *in vacuo* in a 95% yield (660 mg). ^1^H NMR (400 MHz, DMSO-*d*_6_): *δ* 0.91 (t, ^3^*J*_H–H_ = 7.3 Hz, C**H**_3_, 12H); 1.28 (m, C**H**_2_, 8H); 1.54 (m, C**H**_2_, 8H); 3.15 (m, NC**H**_2_, 8H); 3.67 (s, C**H**_3_O, 3H); 6.57–6.79 (m, ^Ar^C**H**, 2H); 6.97–7.23 (m, ^Ar^C**H**, 6H); 7.69–7.95 (m, ^Ar^C**H**, 6H) ppm. ^13^C NMR (101 MHz, DMSO-*d*_6_): *δ* 13.5 (CH_2_**C**H_3_, 4C); 19.2 (**C**H_2_CH_3_, 4C); 23.1 (**C**H_2_CH_2_, 4C); 54.6 (**C**H_3_O, 1C); 57.5 (t, ^3^*J*_H–H_ = 3.0 Hz, N**C**H_2_, 4C); 111.9 (^4-MeO-*meta*^**C**H, 2C); 126.1 (^*ortho*+*para*^**C**H, 6C); 136.8 (^*meta*^**C**H, 4C); 138.8 (^4-MeO-*ortho*^**C**H, 2C); 140.8 (^4-MeO-*ipso*^**C**–Si, 1C); 159.2 (^4-MeO-*para*^**C**, 1C) ppm. ^19^F NMR (376 MHz, DMSO-*d*_6_): *δ* −97.86 ppm (s, 2F). HRMS (ESI+): calcd for C_16_H_36_N [M]^+^ 242.2842, found 242.2844. HRMS (ESI−): calcd for C_19_H_17_F_2_OSi [M]^−^ 327.1022, found 327.1020.

#### Tetrabutylammonium bis(4-methoxyphenyl)phenyldifluorosilicate (4c)

A solution of TBAF (1 M solution in THF; 1.3 mL; 1.3 mmol) was added to a solution of 6b (0.49 g, 1.3 mmol) in dry MeCN (3 mL). Reaction mixture was stirred for 1 h at r.t. After evaporation of the solvent, solid product was formed. Silicate 4c was obtained after drying in a quantitative yield (0.95 g). ^1^H NMR (400 MHz, DMSO-*d*_6_): *δ* 0.93 (t, *J* = 7.3, C**H**_3_, 12H); 1.21–1.38 (m, C**H**_2_, 8H); 1.49–1.62 (m, C**H**_2_, 8H); 3.10–3.19 (m, C**H**_2_, 8H); 3.69 (s, C**H**_3_O, 6H); 6.72 (d, ^3^*J*_H–H_ = 8.4 Hz, ^Ar^C**H**, 4H); 7.06–7.15 (m, ^Ar^C**H**, 3H); 7.74–7.84 (m, ^Ar^C**H**, 4H) ppm. ^13^C NMR (101 MHz, DMSO-*d*_6_) *δ* 13.5 (CH_2_**C**H_3_, 4C); 19.2 (H_2_CH_3_, 4C); 23.1 (**C**H_2_CH_2_, 4C); 54.6 (**C**H_3_O, 2C); 57.5 (t, ^3^*J*_H–H_ = 3.0 Hz, N**C**H_2_, 4C); 111.9 (^4-MeO-*meta*^**C**H, 4C); 125.1 (^*ipso*-Ar^**C**Si, 1C); 126.0 (^*ortho*+*para*^**C**H, 3C); 136.5 (^*meta*^**C**H, 4C); 138.6 (^4-MeO-*ortho*^**C**H, 4C); 140.8 (^4-MeO-^*ipso***C**–Si, 2C); 158.6 (^4-MeO-*para*^**C**, 2C) ppm. ^19^F NMR (376 MHz, DMSO-*d*_6_): *δ* −98.98 ppm. HRMS (ESI+): calcd for C_16_H_36_N [M]^+^ 242.2842, found 242.2844. HRMS (ESI−): calcd for C_20_H_19_F_2_O_2_Si [M]^−^ 357.1128, found 357.1127.

#### Tetrabutylammonium difluorodiphenyl[4-(trifluoromethyl)-phenyl]silicate (4d)

A solution of TBAF (1 M in THF; 3.8 mL; 3.8 mmol) was added to a solution of 6c (1.31 g; 3.78 mmol) in dry MeCN (12 mL). Reaction mixture was stirred for 1 h at r.t. After evaporation of the solvent, solid product was formed, which was purified by trituration with cold toluene. Silicate 4d was obtained after drying in a 72% yield (1.66 g). ^1^H NMR (399.78 MHz, DMSO-*d*_6_): *δ* 0.92 (t, ^3^*J*_H–H_ = 7.3 Hz C**H**_3_, 12H); 1.23–1.34 (q, ^3^*J*_H–H_ = 7.3 Hz, C**H**_2_, 8H); 1.48–1.59 (m, C**H**_2_, 8H); 3.08–3.16 (m, C, ^Ar^C**H**_2_, 8H); 7.11–7.19 (m, C**H**, ^Ar^C**H**, 6H); 7.46 (d, ^3^*J*_H–H_ = 8.1 Hz, ^4-CF_3_^C**H**, 2H); 7.88–7.93 (m, C**H**, ^Ar^C**H**, 4H); 8.04 (d, ^3^*J*_H–H_ = 8.1 Hz, ^4-CF_3_^C**H**, 2H) ppm. ^13^C NMR (100.53 MHz, DMSO-*d*_6_): *δ* 13.5 (CH_2_**C**H_3_, 4C); 19.2 (**C**H_2_CH_3_, 4C); 23.0 (**C**H_2_CH_2_, 4C); 57.5 (t, ^1^*J*_C–N_ = 3.0 Hz, ^Ar^C**H**, Si, 2C); 157.0 (^4-CF_3_-*ipso*^**C**–Si, 1C) ppm. ^19^F NMR (376 MHz, DMSO-*d*_6_): *δ* −60.56 (C**F**_3_, 3F); −97.22 (Si**F**_2_^−^, 2F) ppm. HRMS (ESI+): calcd for C_16_H_36_N [M]^+^ 242.2842, found 242.2843. HRMS (ESI−): calcd for C_19_H_14_F_5_Si [M]^−^ 365.0790, found 365.0788.

### Fluorinations

#### General procedure

5 mL Schlenk flask was charged with fluorinating reagent (2.0 equiv.), substrate (1.0 equiv., 20 mg) and CD_3_CN (0.7 mL). The flask was sealed and heated on metallic block to 85 °C for 24 h. After cooling, the samples were measured by ^1^H NMR (16 scans, 20 s relaxation delay) and the conversions were determined from characteristic peaks of the products given below.

#### 1-Fluorooctane (14)

^1^H NMR (399.94 MHz, CD_3_CN): *δ* 4.44 (dt, ^2^*J*_H–F_ = 47.5 Hz, ^3^*J*_H–H_ = 6.2 Hz, C**H**_2_F, 2H) ppm.

#### 2-Fluorooctane (5)

^1^H NMR (399.94 MHz, CD_3_CN): *δ* 4.64 (ddqd, ^2^*J*_H–F_ = 49.3 Hz, ^3^*J*_H–H_ = 7.5 Hz, ^3^*J*_H–H_ = 6.2 Hz, ^3^*J*_H–H_ = 4.7 Hz, C**H**F, 1H) ppm.

#### Oct-1-ene (7a)

^1^H NMR (399.94 MHz, CD_3_CN): *δ* 5.85 (ddt, ^3^*J*_H–H_ = 17.0 Hz, ^3^*J*_H–H_ = 10.2 Hz, ^3^*J*_H–H_ = 6.7 Hz, C**H**=CH_2_, 1H) ppm.

#### Oct-2-ene (7b)

^1^H NMR (399.94 MHz, CD_3_CN): *δ* 5.33–5.53 (m, C**H**=C**H**, 2H) ppm.

#### 1-(Fluoroethyl)benzene (8)

^1^H NMR (399.78 MHz, CD_3_CN): *δ* 5.62 (dq, ^2^*J*_H–F_ = 47.8 Hz, ^3^*J*_H–H_ = 6.4 Hz, C**H**F, 1H) ppm.

#### Styrene

^1^H NMR (399.78 MHz, CD_3_CN): *δ* 6.72 (dd, ^3^*J*_H–H_ = 17.7 Hz, ^3^*J*_H–H_ = 11.0 Hz, C**H**=CH_2_, 1H) ppm.

#### Fluorocyclohexane (9)

^1^H NMR (399.78 MHz, CD_3_CN): *δ* 4.2–4.4 (dm, ^2^*J*_H–F_ = 48.8 Hz, C**H**F, 1H) ppm.

#### Cyclohexene

^1^H NMR (399.78 MHz, CD_3_CN): *δ* 5.65 (s, C**H**=C**H**, 2H) ppm.

#### Fluorocyclopentane (10)

^1^H NMR (399.78 MHz, CD_3_CN): *δ* 5.02–5.20 (dm, ^2^*J*_H–F_ = 55.0 Hz, C**H**F, 1H) ppm.

#### Cyclopentene

^1^H NMR (399.78 MHz, CD_3_CN): *δ* 5.72 (m, C**H**=C**H**, 2H) ppm.

#### Benzyl 2-fluoropropanoate (11)

^1^H NMR (399.78 MHz, CD_3_CN): *δ* 5.03–5.20 (dq, ^2^*J*_H–F_ = 48.2 Hz, ^3^*J*_H–H_ = 6.8 Hz, CHF, 1H) ppm.

#### Benzyl acrylate

^1^H NMR (399.78 MHz, CD_3_CN): *δ* 6.17 (dd, ^3^*J*_H–H_ = 17.4 Hz, ^3^*J*_H–H_ = 10.4 Hz, C**H**=CH_2_, 1H) ppm.

#### 2-Fluoro-1-phenylpropan-1-one (12)

^1^H NMR (399.78 MHz, CD_3_CN): *δ* 5.80–6.02 (dq, ^2^*J*_H–F_ = 48.0 Hz, ^3^*J*_H–H_ = 6.7 Hz, C**H**F, 1H) ppm.

#### 1-Phenylprop-2-en-1-one

^1^H NMR (399.78 MHz, CD_3_CN): *δ* 6.33 (dd, ^3^*J*_H–H_ = 17.1 Hz, ^2^*J*_H–H_ = 1.9 Hz, CH=C**H**_2_, 1H) ppm.

Results of the preliminary computations, copies of NMR spectra and xyz files of all computed structures are given in the ESI.†

## Conclusions

Starting from fluorosilanes 6a–6c and commercial solution of TBAF (1) in THF, we synthesized three new difluorosilicates 4b–4d containing one or two electron donating methoxy groups or one electron withdrawing trifluoromethyl group in the aryl rings. We found that TBAT analogues 4b, 4c bearing one or two electron donating groups gave in most cases (2-bromooctane (2a), 2-iodooctane (2c), octan-2-mesylate (2b), (1-bromoethyl)benzene (8) and bromocyclopentane (10)) better results in nucleophilic fluorination of secondary substrates than TBAF (1) and TBAT (4a), while the presence of the electron withdrawing group led to inferior results. On the other hand, simple TBAF (1) gave better yields of fluorination of 2-chlorooctane (2d) and bromocyclohexane (9), indicating probably different mechanism of the fluorination/elimination complex. Attempts to improve further fluorination selectivity by adding more electron-donating groups failed due to low stability of the corresponding triaryldifluorosilicates, as was confirmed by ^19^F NMR experiments. DFT study of the decomposition of difluorosilicates to fluorosilane–fluoride complexes disclosed that the activation energy decreases with increased electron density on the modified phenyl group in an order 4-CF_3_C_6_H_4_ > C_6_H_5_ > 4-MeOC_6_H_4_.

## Data availability

The data supporting this article have been included as part of the ESI.†

## Conflicts of interest

There are no conflicts to declare.

## Supplementary Material

RA-014-D4RA04332D-s001

RA-014-D4RA04332D-s002
