# Supplementary material for: Modified aryldifluorophenylsilicates with improved activity and selectivity in nucleophilic fluorination of secondary substrates
Source: RSC Adv. 2024 Jul 15;14(31):22326–34. doi: 10.1039/d4ra04332d (PMC11247957; doi:10.1039/d4ra04332d)
Supplement: RA-014-D4RA04332D-s001 [file RA-014-D4RA04332D-s001.pdf]

Electronic Supplementary Material (ESI) for RSC Advances.
This journal is © The Royal Society of Chemistry 2024

Modified aryldifluorophenylsilicates with improved activity and selectivity in nucleophilic fluorination of secondary halides

Adam Hroch,[a] Michal Trojan,[a] Evelin Gruden,[b] Josef Cvačka,[c] Jan Čejka,[d] Gašper Tavčar,[b] Markéta Rybáčková[a] and Jaroslav Kvíčala*[a]

[a] *Department of Organic Chemistry, University of Chemistry and Technology, Prague, Technická 5, 166 28 Prague 6, Czech Republic.*
[b] *Department of Inorganic Chemistry and Technology, "Jožef Stefan" Institute, Jamova cesta 39, Ljubljana, Slovenia.*
[c] *Institute of Organic Chemistry and Biochemistry of the Czech Academy of Sciences, Flemingovo náměstí 542/2, 160 00 Prague 6, Czech Republic.*
[d] *Department of Solid State Chemistry, University of Chemistry and Technology, Prague, Technická 5, 166 28 Prague 6, Czech Republic.*
* Corresponding author. E-mail address: kvicalaj@vscht.cz (Jaroslav Kvíčala)

SUPPORTING INFORMATION

Table of Contents

1. **Additional computations** 1
2. **Example of the ^{1}H NMR analysis of the fluorination results** 5
3. **NMR spectra of the synthesized compounds** 6
4. **Crystallographic data of difluorosilicates 4b-4d** 21
5. **XYZ files of computed structures (energies in kJ/mol)** 22
6. **References** 51

1. Additional computations

Preliminary computations were performed using Gaussian16[1] program suite using pure M-06L functional,[2] which enabled the use of the RI (resolution of identity) approach,[3] together with the double-ζ def2-SVP basis set,[4] which greatly accelerated the initial calculations. To better describe the anionic structures, we also used double-ζ def2-SVPD basis set with additional diffuse functions.[5] Solvent (MeCN) was simulated using the SMD variant of the IEF-PCM method.[6]

Higher level productive calculations were accomplished using ORCA computational program, [7] which uses the efficient RIJCOSX approximation[8] to accelerate hybrid functional computations. We employed

the M06-2X hybrid functional[2] together with the minimally augmented ma-def2-TZVP basis set.[9] Weigend's universal auxiliary basis set was used for the RI approximation calculations.[10] The MeCN solvent was simulated with the CPCM method[11] and the description of the non-covalent interactions was improved by the dispersion correction.[12]

The equilibrium among difluorosilicates **18** and fluorosilane-fluoride complexes **19** was studied for three difluorosilicates, viz. tetramethylammonium difluorotriphenylsilicate (**18a**), tetramethylammonium difluoro(4-methoxyphenyl)diphenylsilicate (**18b**) and tetramethylammonium difluorodiphenyl[4-(trifluoromethyl)phenyl]silicate (**18c**), with the aim to find how substitution with electron donating or electron withdrawing group will influence transition state energy of the equilibrium.

First, conformational space of all three starting difluorosilicates **18** was studied at both levels. In all cases, the tetramethylammonium cation is oriented in a skewed position relatively to the F-Si-F linear bonds. For triphenylsilicate **18a**, two main conformers were found, viz. more stable conformation **18aA** with butterfly wings-like arrangement of two phenyl rings oriented towards the ammonium cation, and a less stable conformation **18aB** with propeller-like arrangement of the three phenyl rings. Both structures and their relative free Gibbs energies are shown on Figure S1 (structures computed by the productive ORCA calculations).

top view

side view

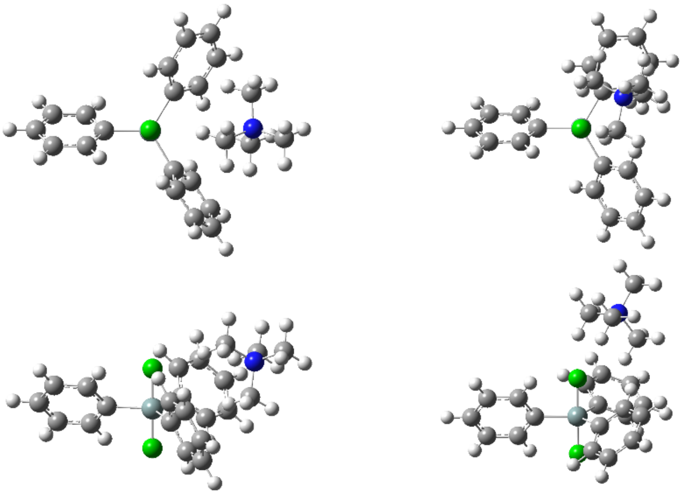

Relative energies	Structure:	**18aA**	**18aB**
(kJ/mol): M06L/def2-SVPD		0.0	2.4
M06-2X/ma-def2-TZVP		0.0	4.7

Figure S1: Butterfly wing **18aA** and propeller **18aB** structures and their relative free Gibbs energies

Twelve possible minimal geometries were found for the difluorosilicate **18b** modified with 4-methoxyphenyl group, six butterfly wing-like and six propeller like, at the lower level calculations. 4-Methoxyphenyl group can be coordinated to the ammonium cation from one or the other side, or can be arranged against the ammonium cation. Furthermore, the methyl of the methoxy group can be arranged towards the ammonium cation or against it. In an analogy to the structures **18a**, the butterfly wing-like structures were more stable than the propeller-like structures. In an analogy to the crystal structure of TBAT analogue **4b**, the preferred structure **18bA** contains the methoxy group adjacent to the ammonium cation with the methyl group oriented towards it. This structure, the respective propeller-like structure **18bB** and their relative free Gibbs energies are shown on Figure S2.

top view

side view

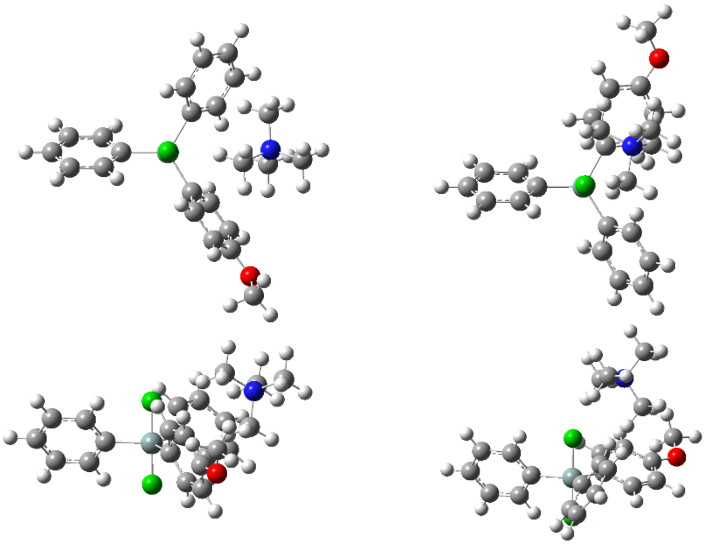

Relative energies	Structure:	**18bA**	**18bB**
(kJ/mol): M06L/def2-SVPD		0.0	4.9
M06-2X/ma-def2-TZVP		0.0	8.2

Figure S2: Butterfly wing **18bA** and propeller **18bB** structures and their relative free Gibbs energies

Similarly, three possible butterfly wing-like structures and three propeller-like structures can be found for the difluorosilicate **18c** modified with the electron withdrawing 4-(trifluoromethyl)phenyl group. The trifluoromethyl group could be positioned on one or the other phenyl group adjacent to the ammonium cation, or on the phenyl group positioned against it. The most stable computed butterfly wing-like structure **18cA** contains the CF_3 positioned against the ammonium cation and does not correspond to the crystal structure of **4d**, which has the CF_3 group oriented towards the ammonium cation. Again, the butterfly wing-like structure **18cA** is more stable than the respective propeller-like structure **18cB** (see Figure S3 for the structures and their Gibbs free energies).

top view

side view

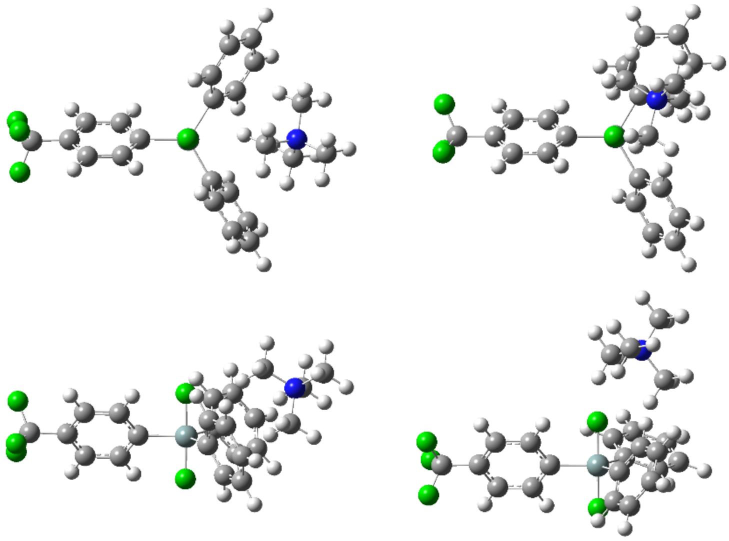

Relative energies	Structure:	**18cA**	**18cB**
(kJ/mol): M06L/def2-SVPD		0.0	5.2
M06-2X/ma-def2-TZVP		0.0	9.3

Figure S3: Butterfly wing **18cA** and propeller **18cB** structures and their relative free Gibbs energies

Most stable butterfly wing-like structures **18aA**, **18bA** and **18cA** were used as the starting geometries for the search of transition states. The PES of the decomposition of difluorosilicates **18** to fluorosilane-fluoride complexes **19**, computed at the higher level, is shown in Figure 6 of the article, while the corresponding PES computed at the lower level is shown in Figure S4.

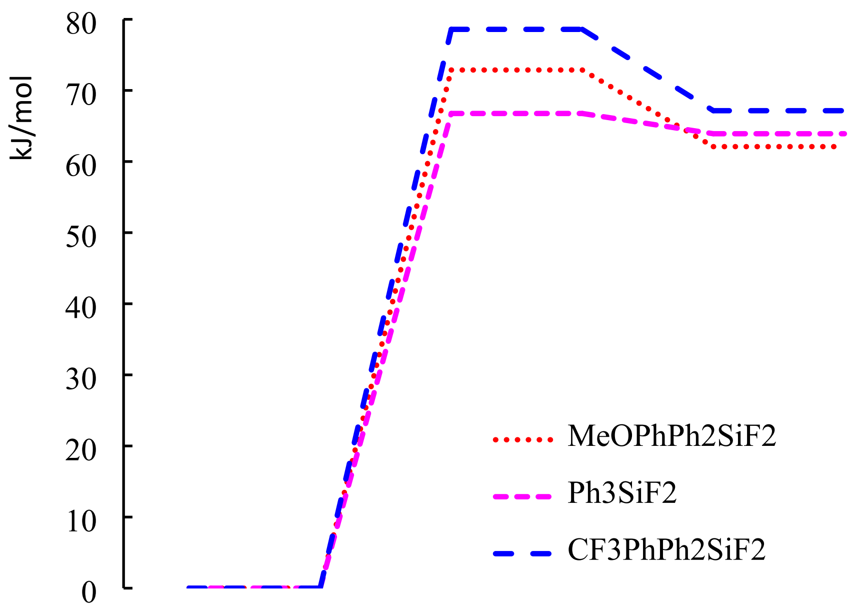

Figure S4. PES of decomposition of difluorosilicates **18a**-**18c** to fluorosilane-fluoride complexes **19a**-**19c**, computed at the lower M06L/def2-SVPD level.

The results of the computations at the lower level differ from those at the higher level. The presence of the electron donating methoxy group resulted in a little higher transition state energy, while substitution with the electron-withdrawing trifluoromethyl group gave higher transition state energy. As expected, the use of pure functional led to somewhat lower transition state energies compared to the results using hybrid functional.

2. Example of the ^{1}H NMR analysis of the fluorination results

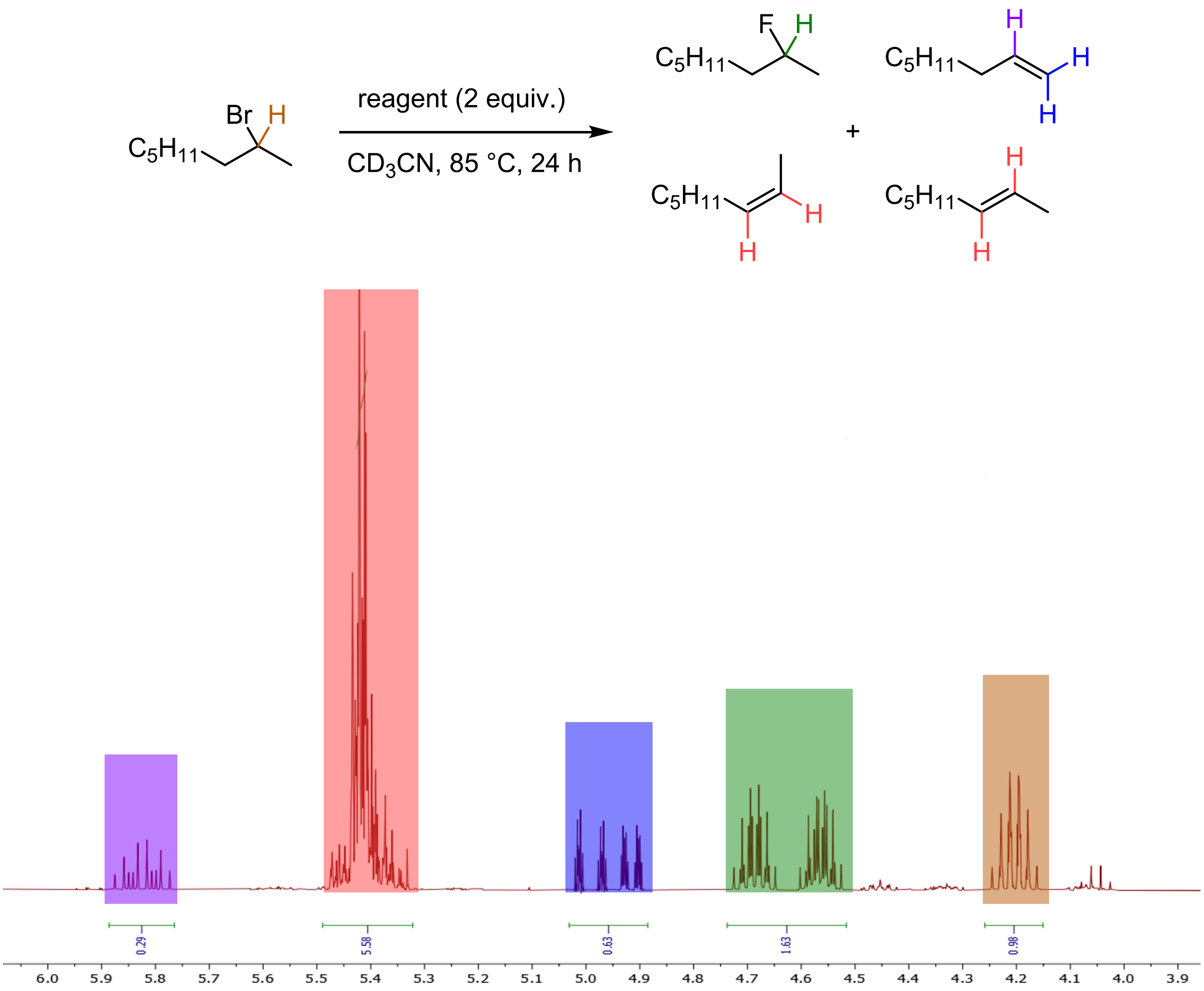

Figure S5. Example of the analysis of the reaction mixture after fluorination of 2-bromoctane (**2a**) with TBAT

3. NMR spectra of the synthesized compounds

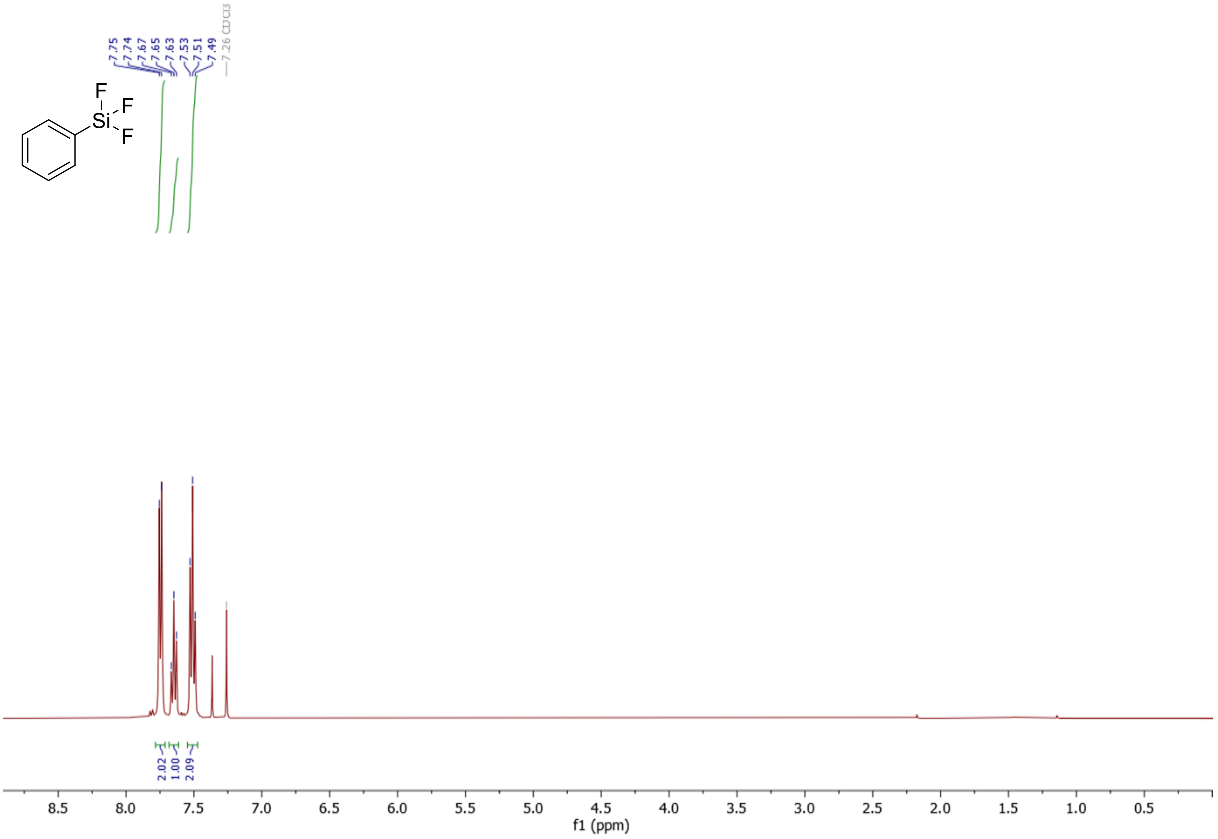

Figure S6: ^{1}H NMR spectrum of $PhSiF_3$ in $CDCl_3$

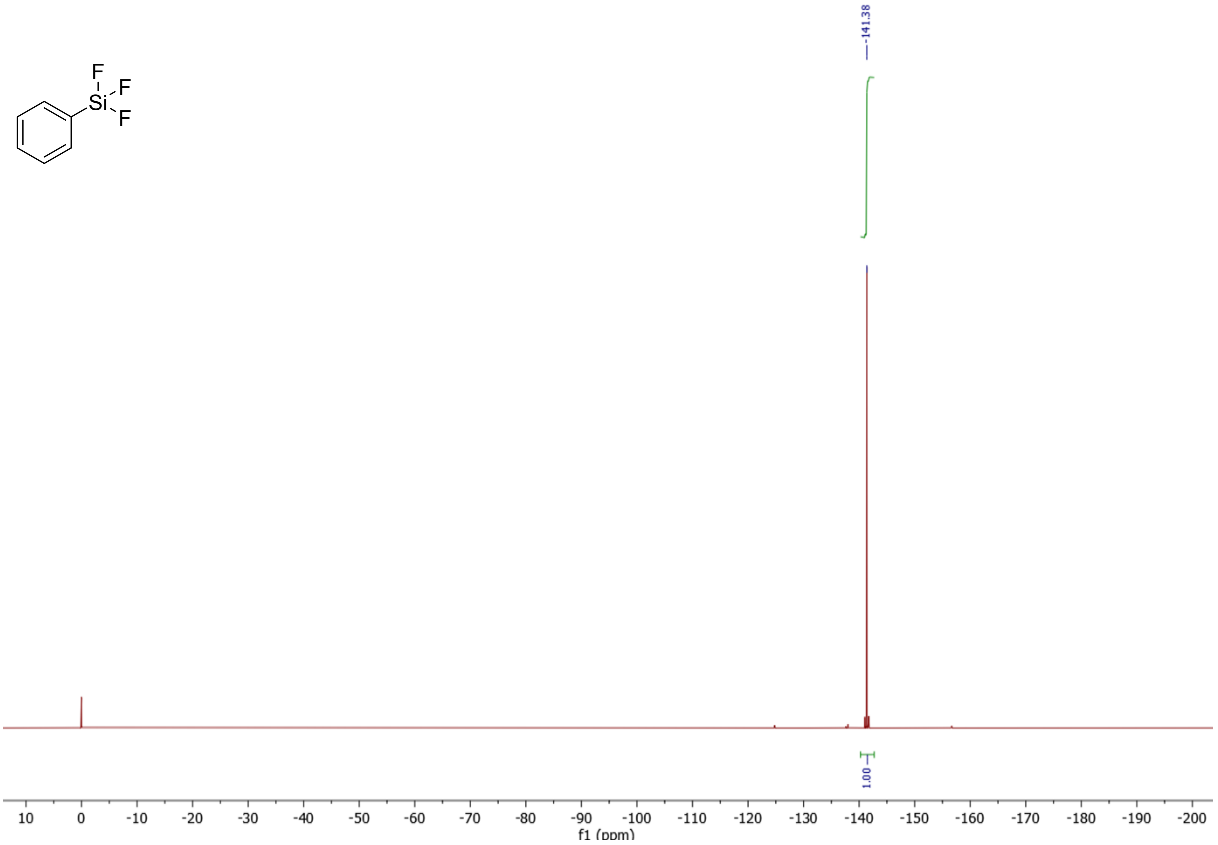

Figure S7: ^{19}F NMR spectrum of $PhSiF_3$ in $CDCl_3$

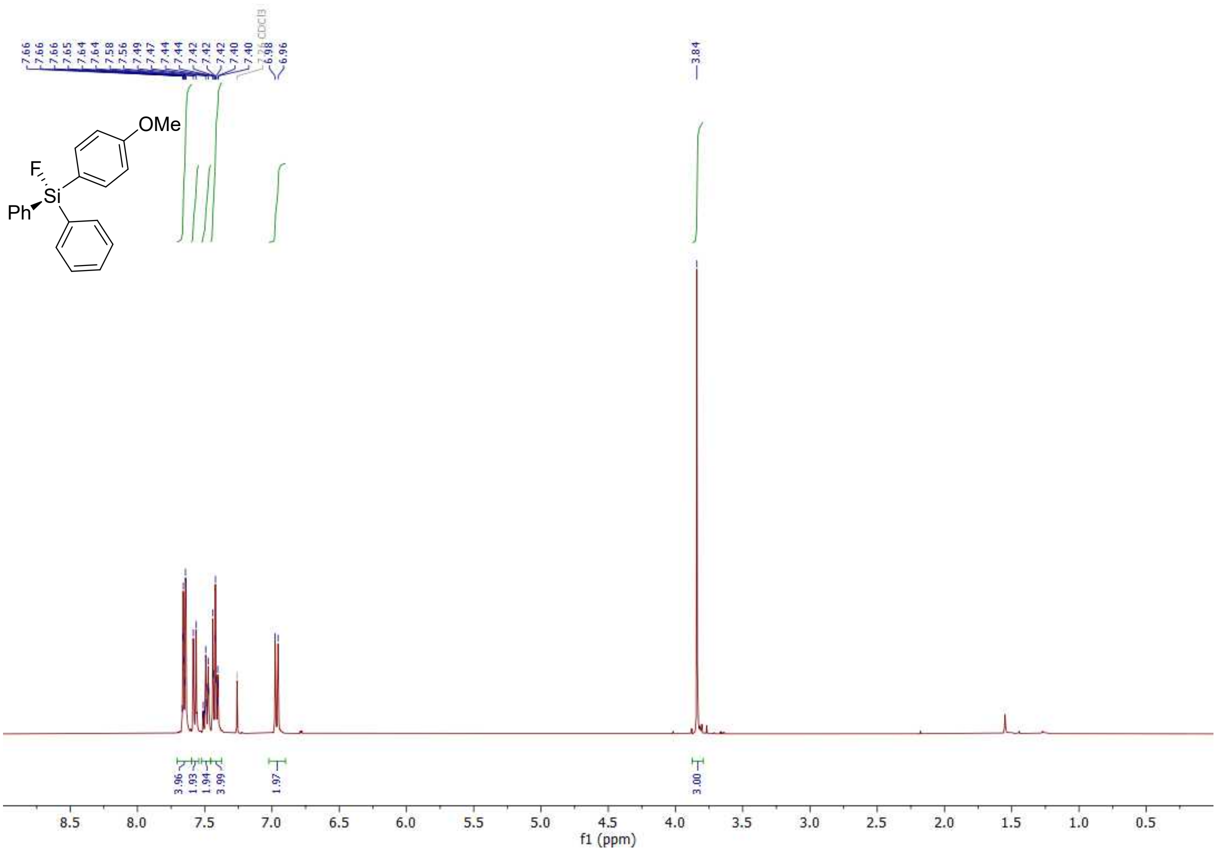

Figure S8: ^{1}H NMR spectrum of **6a** in $CDCl_3$

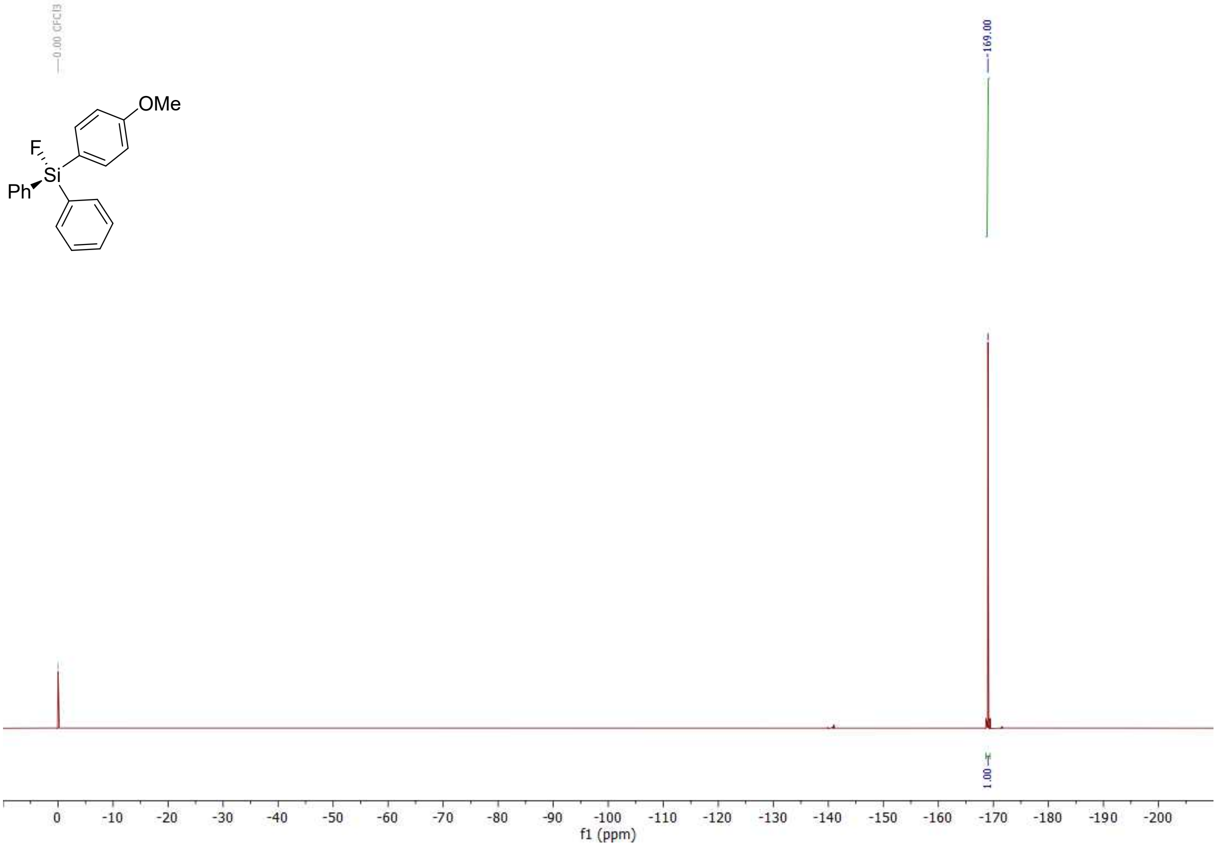

Figure S9: ^{19}F NMR spectrum of **6a** in $CDCl_3$

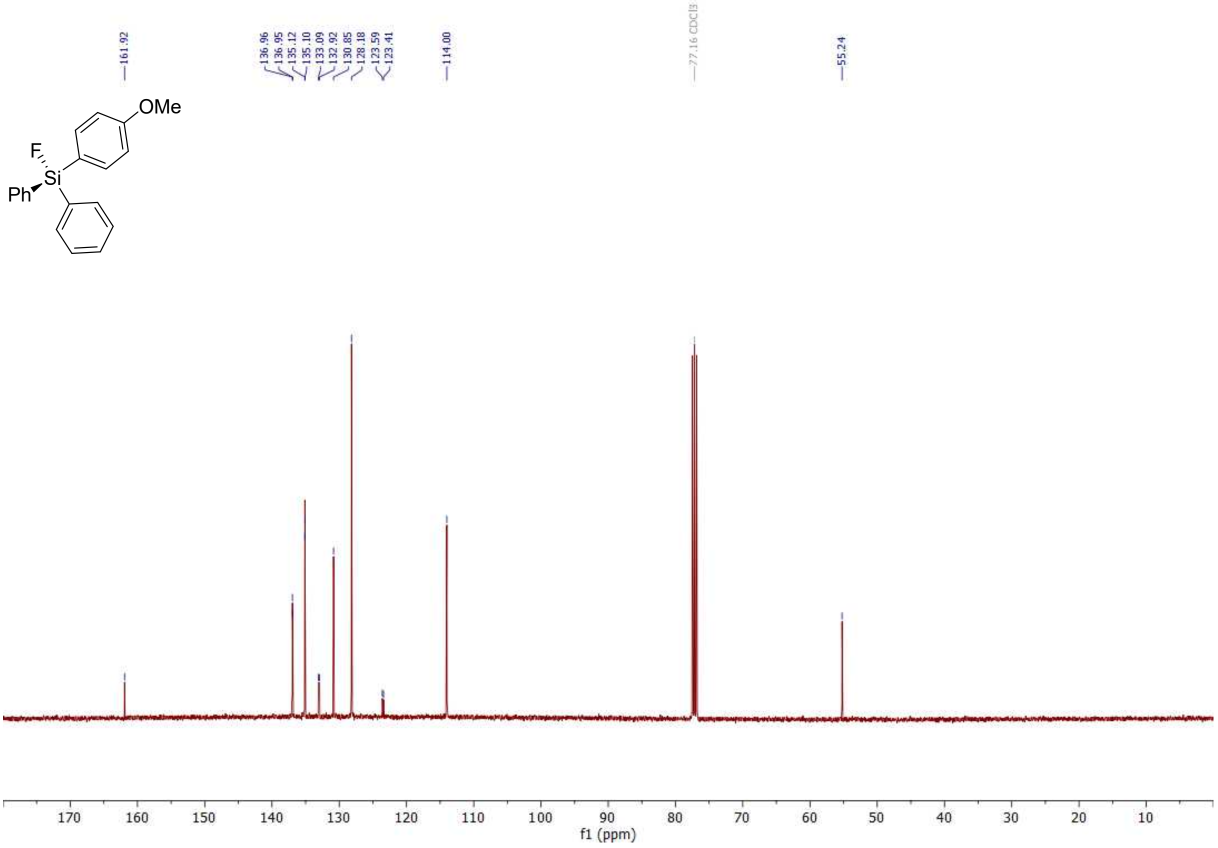

Figure S10: ^{13}C NMR spectrum of **6a** in $CDCl_3$

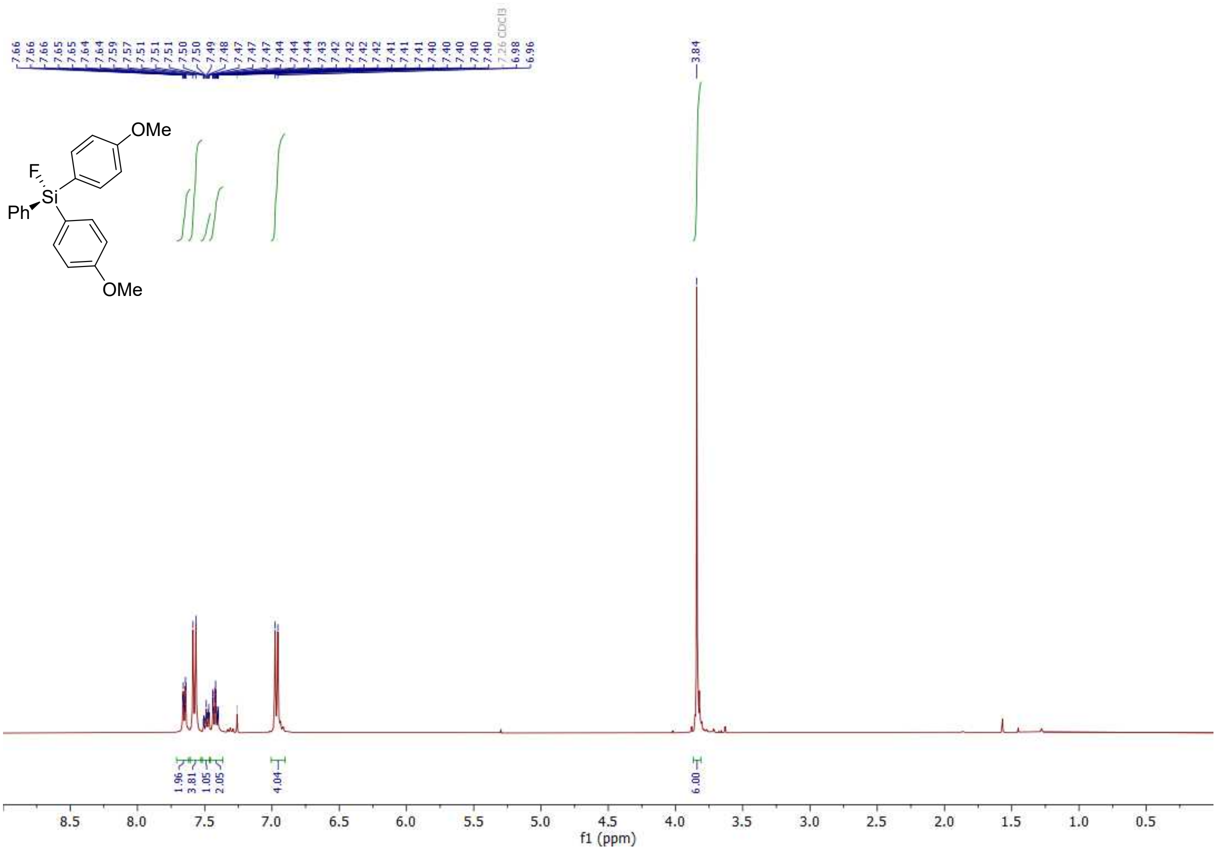

Figure S11: ^{1}H NMR spectrum of **6b** in $CDCl_3$

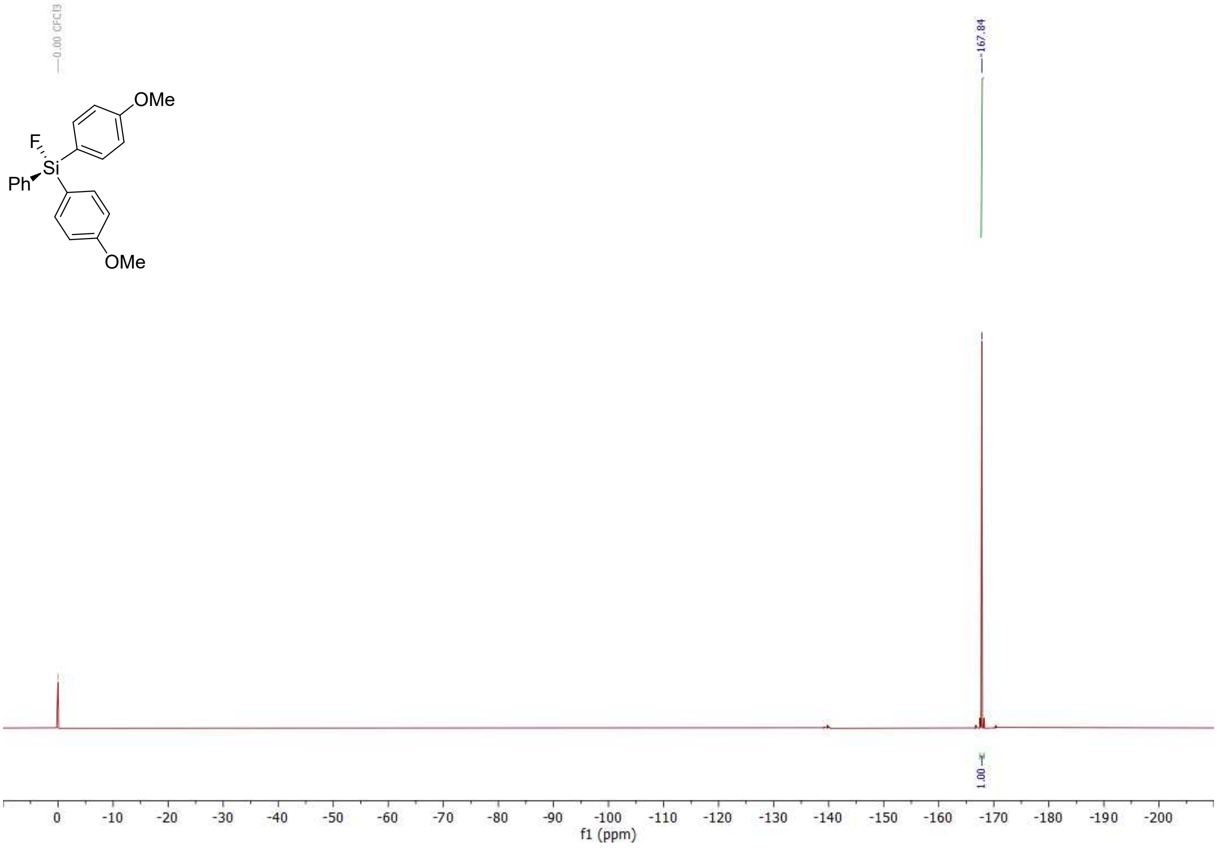

Figure S12: ^{19}F NMR spectrum of **6b** in $CDCl_3$

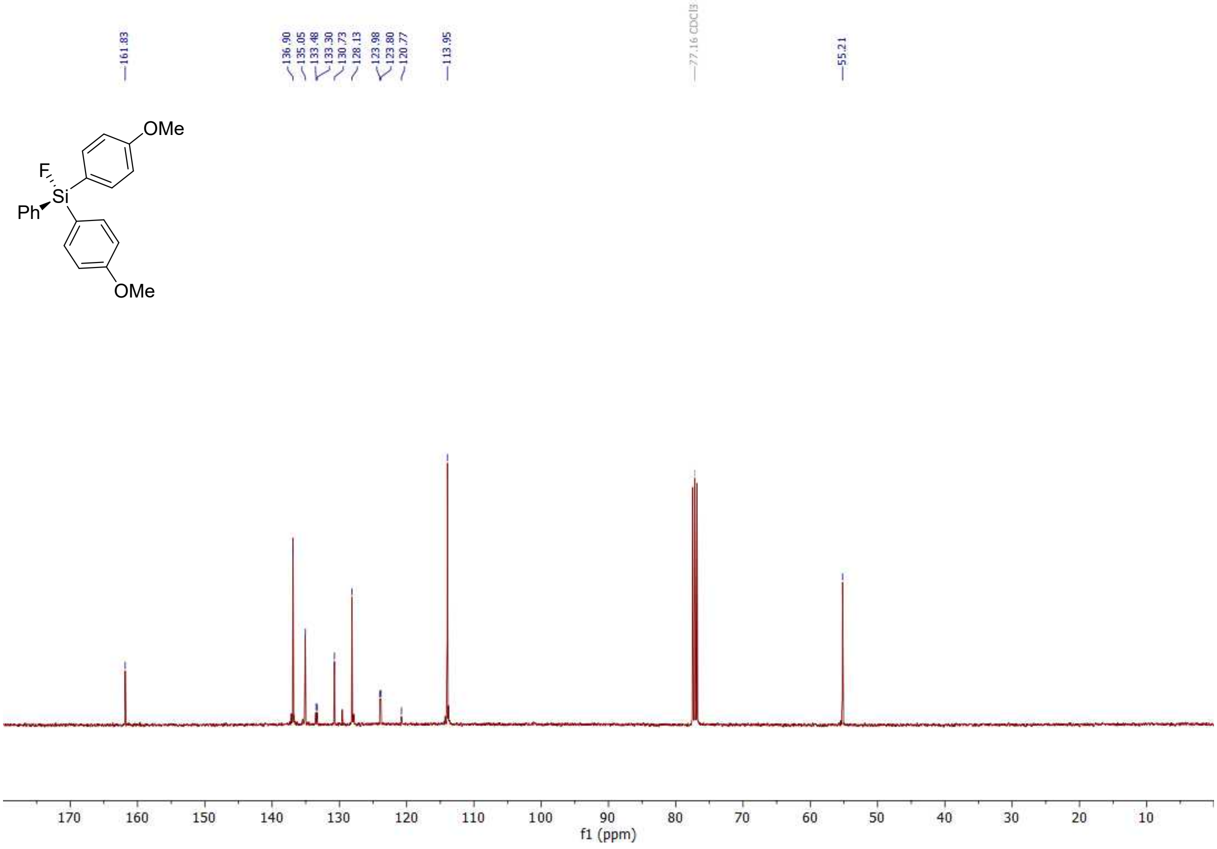

Figure S13: ^{13}C NMR spectrum of **6b** in $CDCl_3$

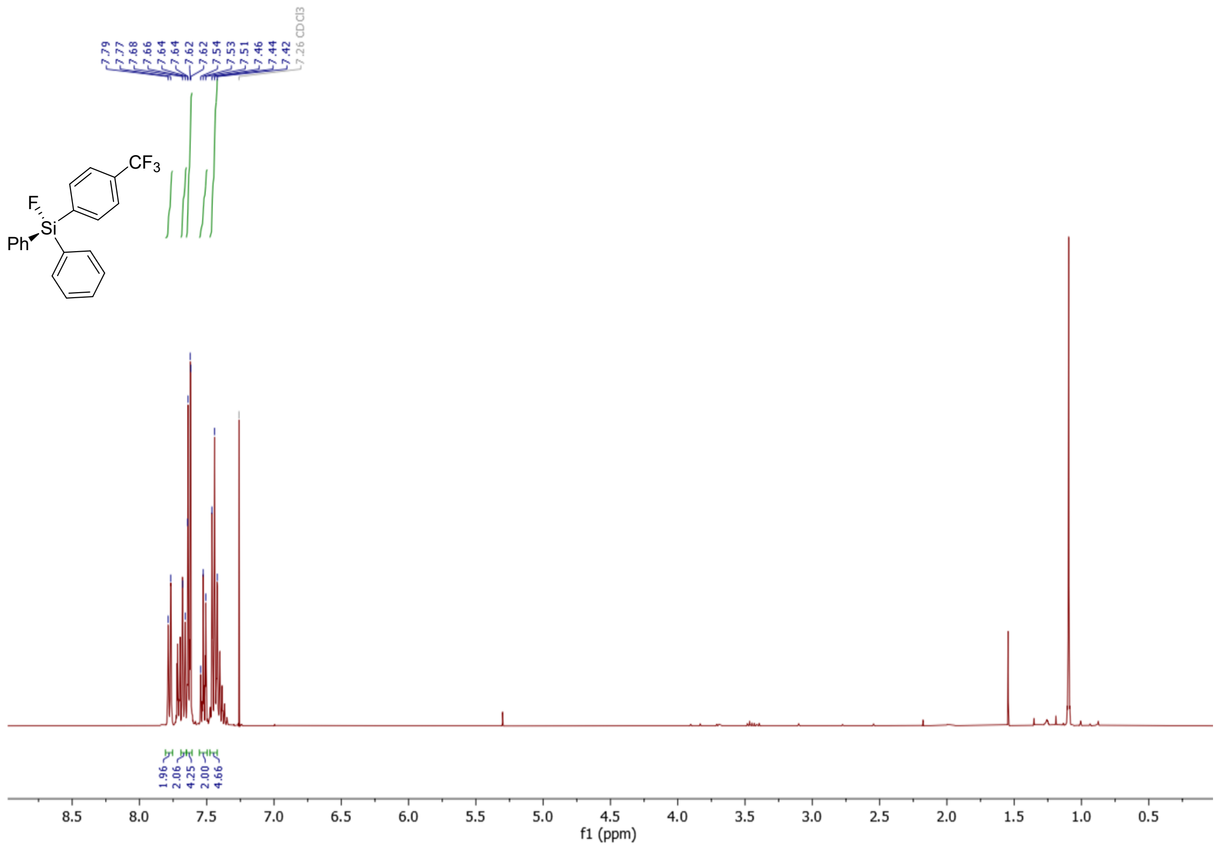

Figure S14: ^{1}H NMR spectrum of **6c** in $CDCl_3$

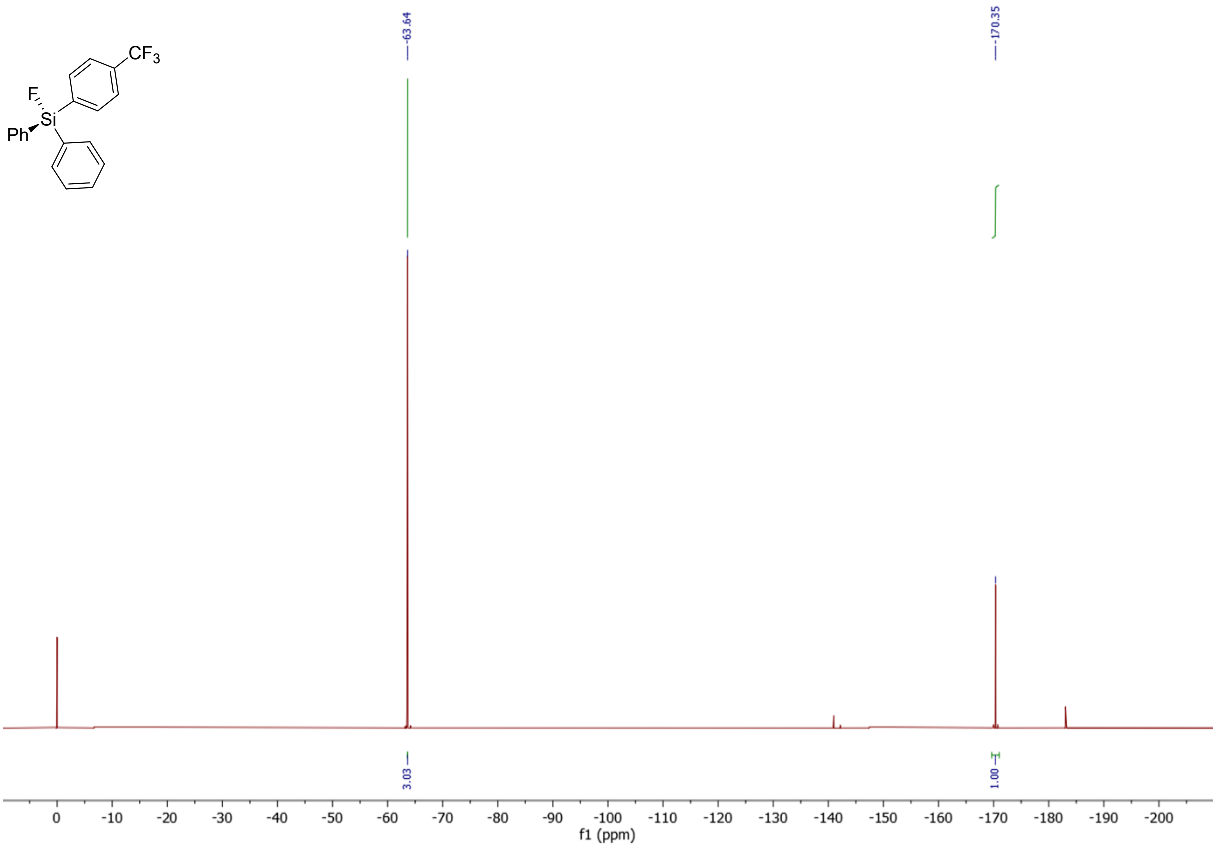

Figure S15: ^{19}F NMR spectrum of **6c** in $CDCl_3$

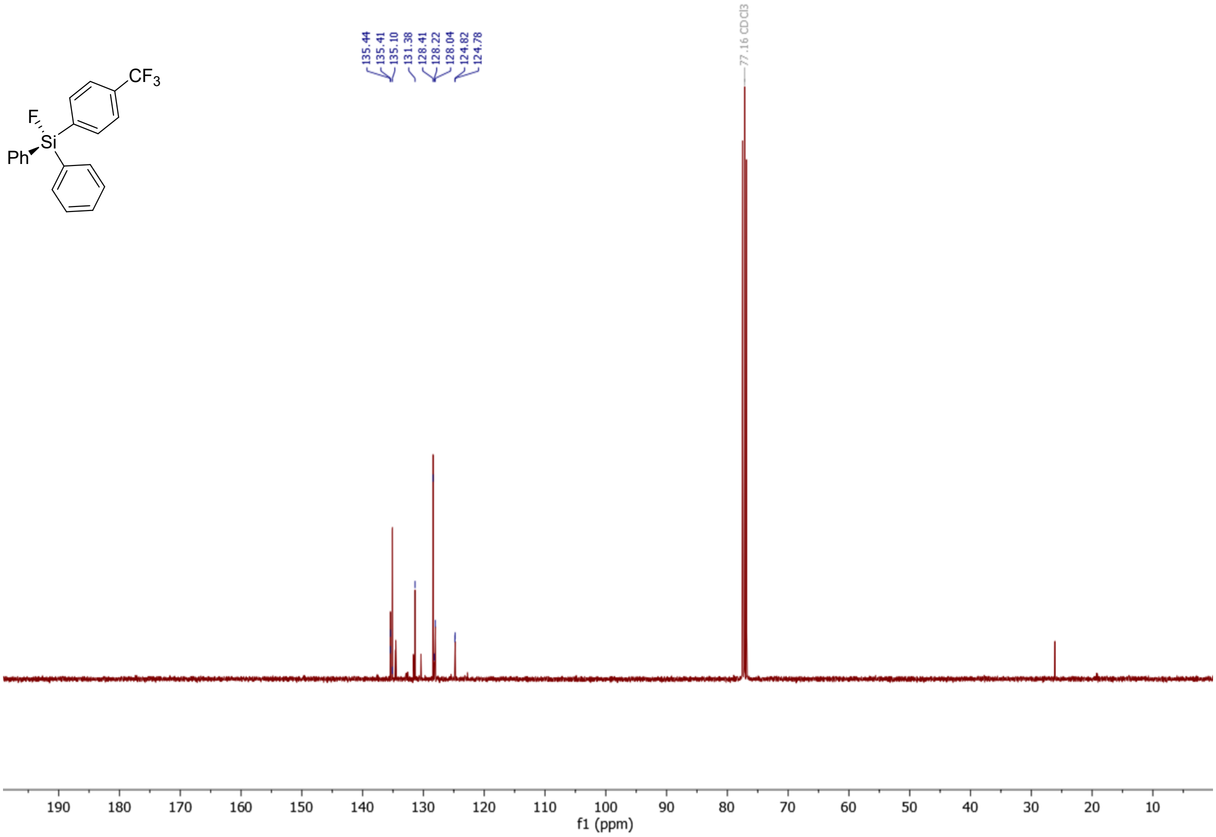

Figure S16: ^{13}C NMR spectrum of **6c** in $CDCl_3$

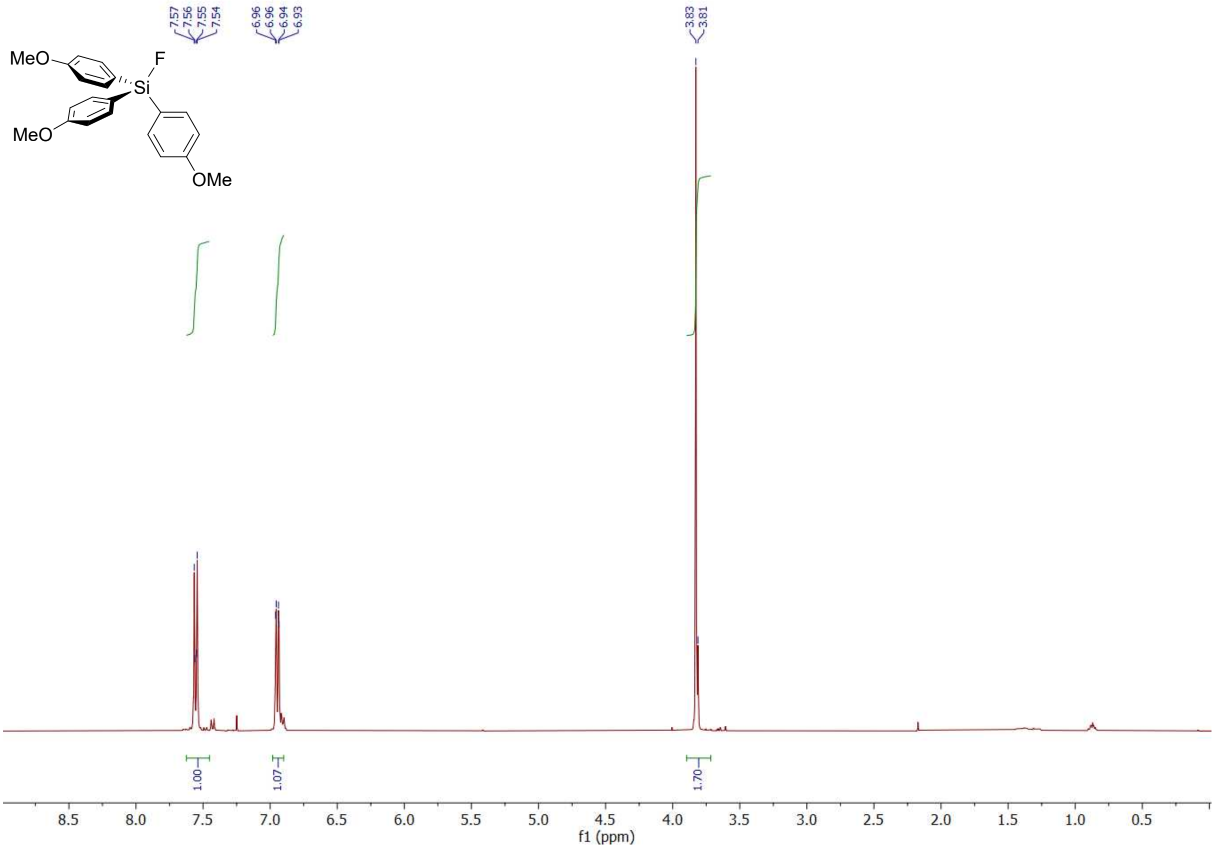

Figure S17: ^{1}H NMR spectrum of **6d** in $CDCl_3$

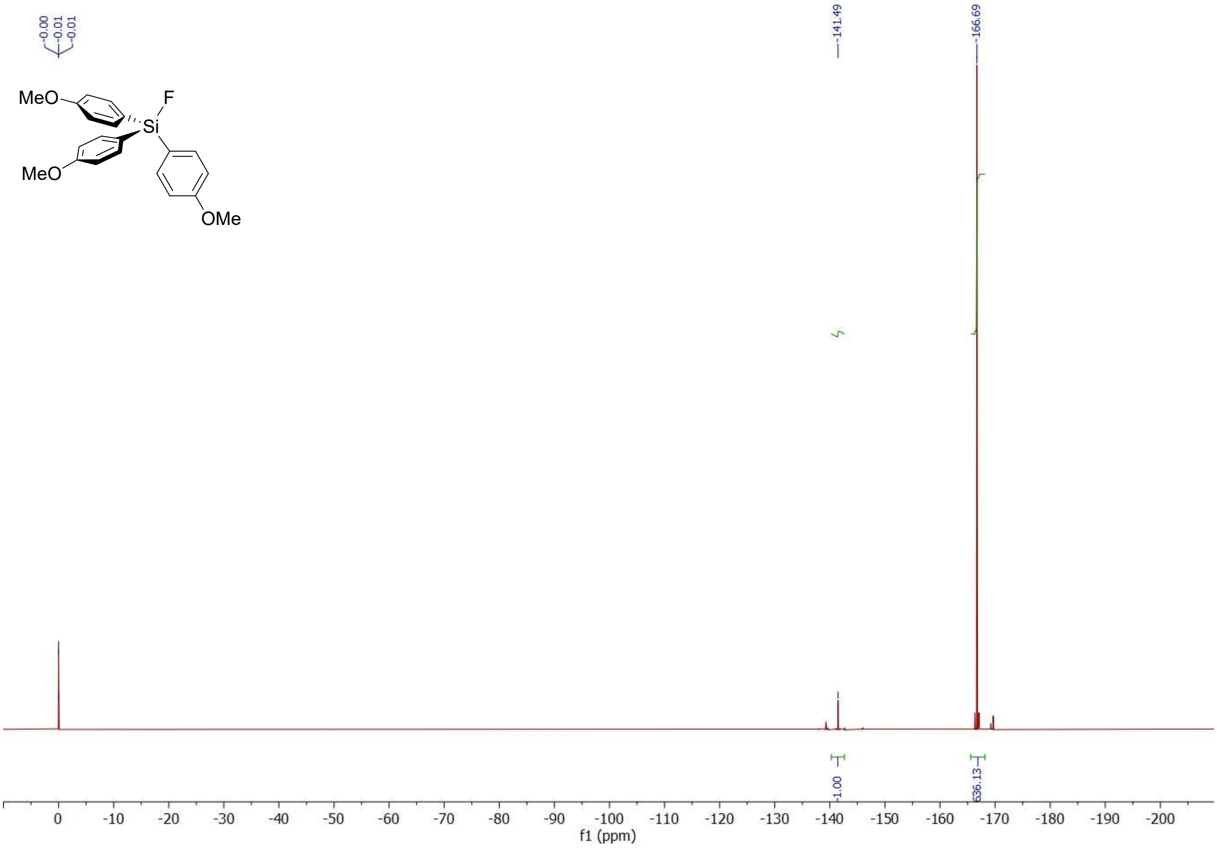

Figure S18: ^{19}F NMR spectrum of **6d** in $CDCl_3$

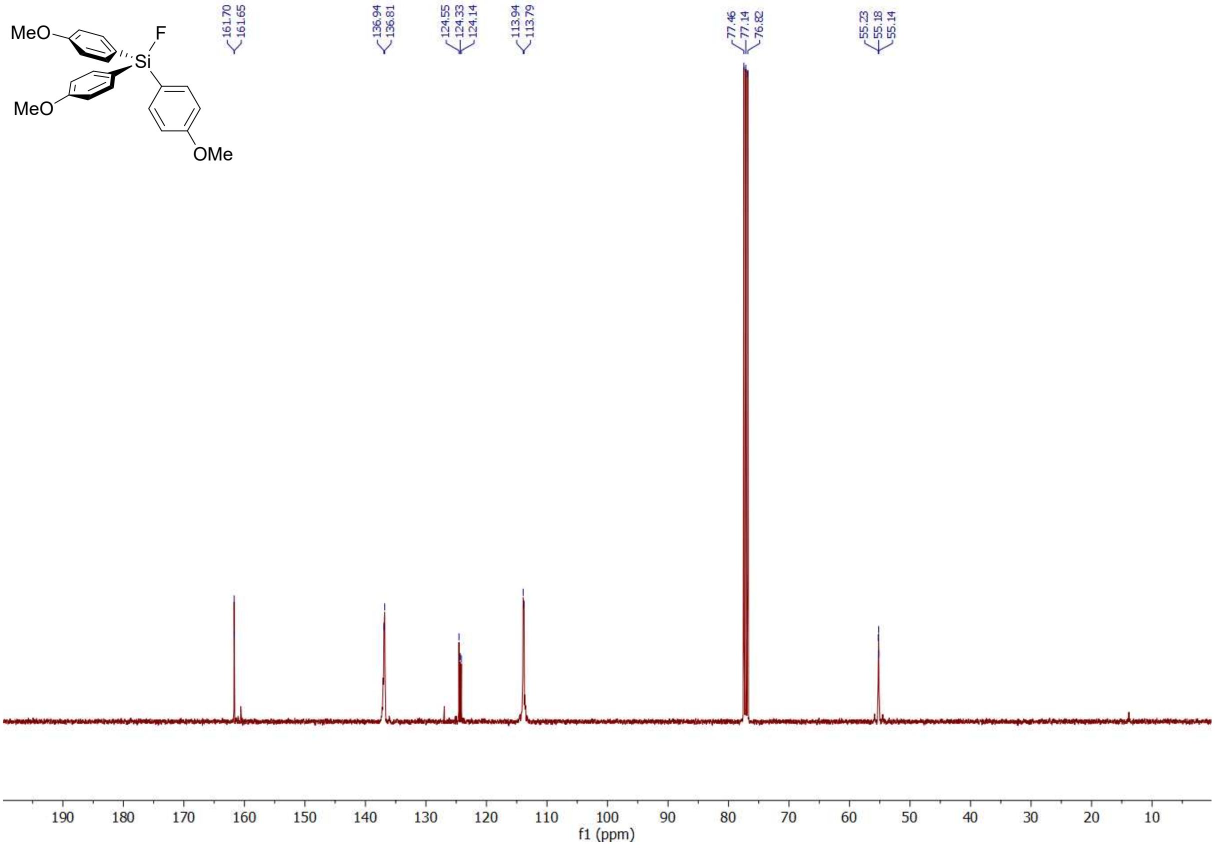

Figure S19: ^{13}C NMR spectrum of **6d** in $CDCl_3$

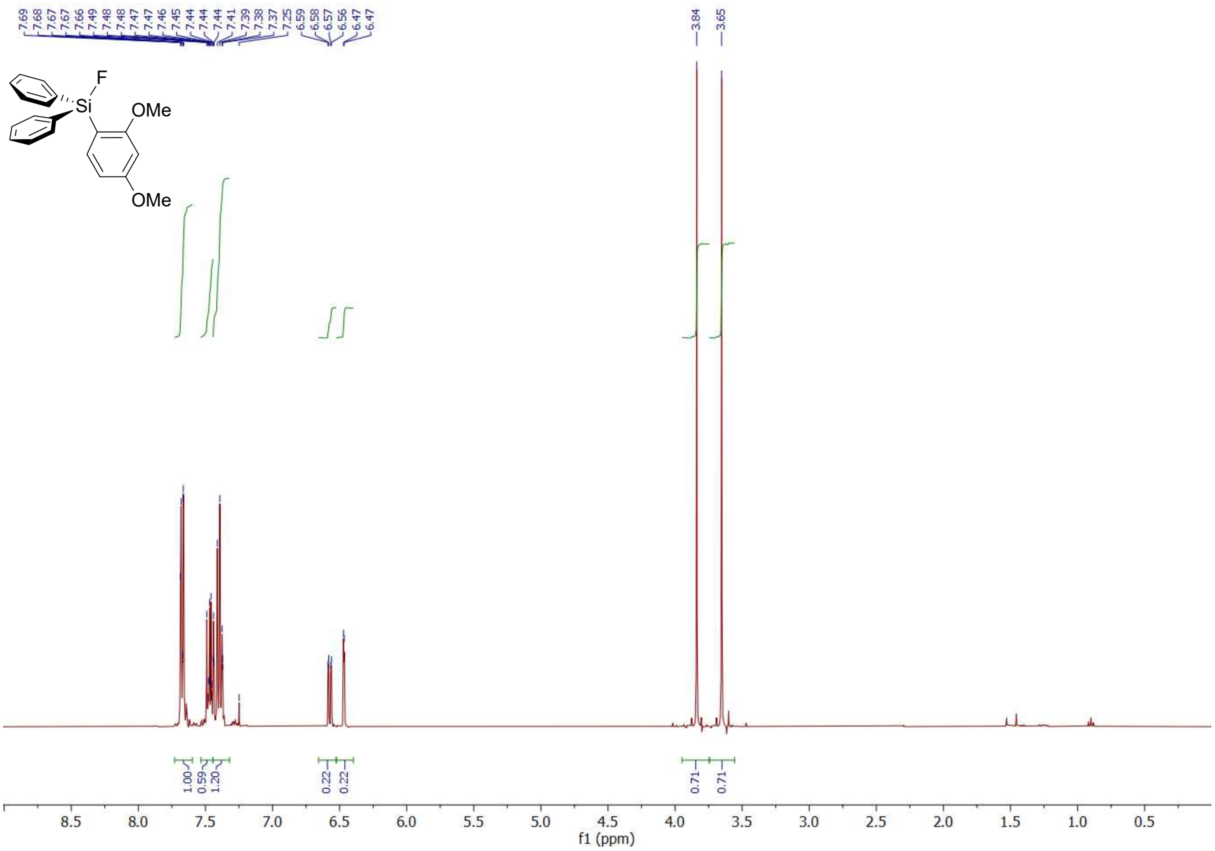

Figure S20: ^{1}H NMR spectrum of **6e** in $CDCl_3$

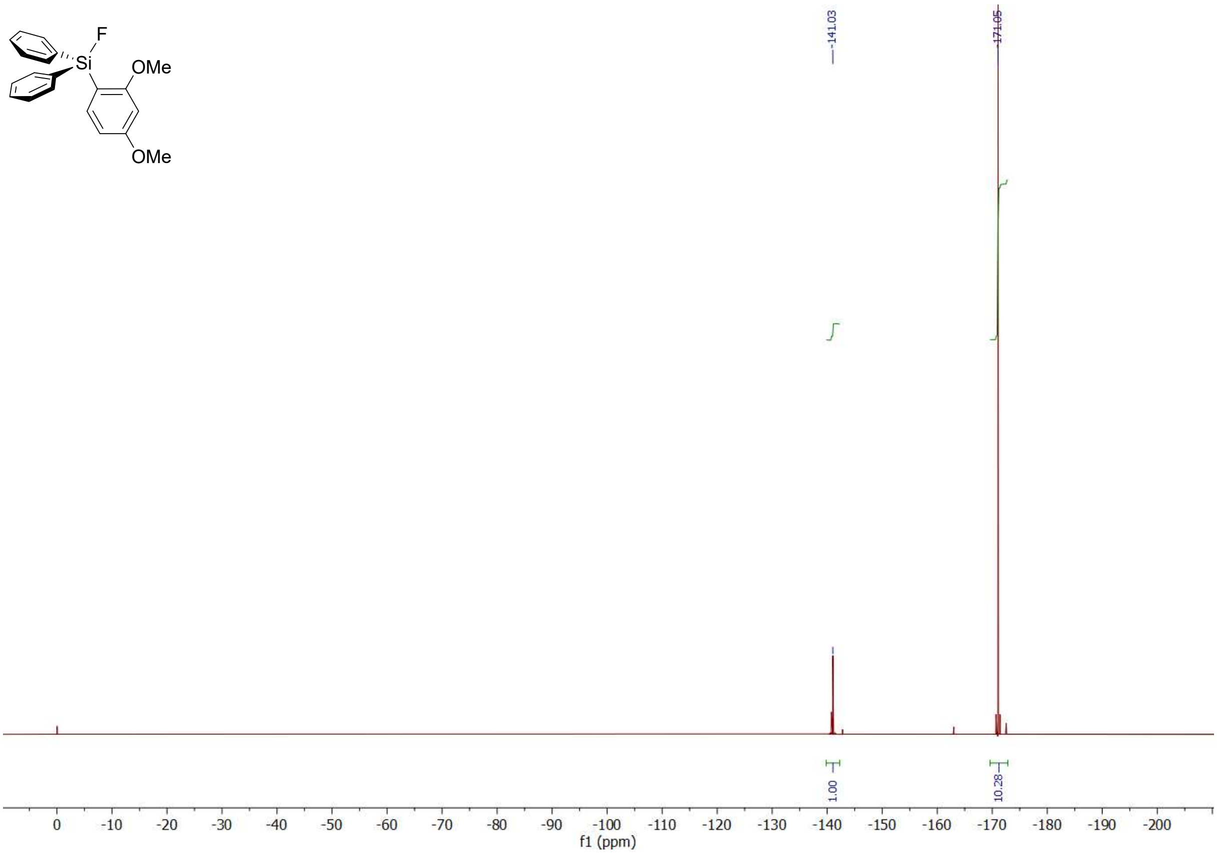

Figure S21: ^{19}F NMR spectrum of **6e** in $CDCl_3$

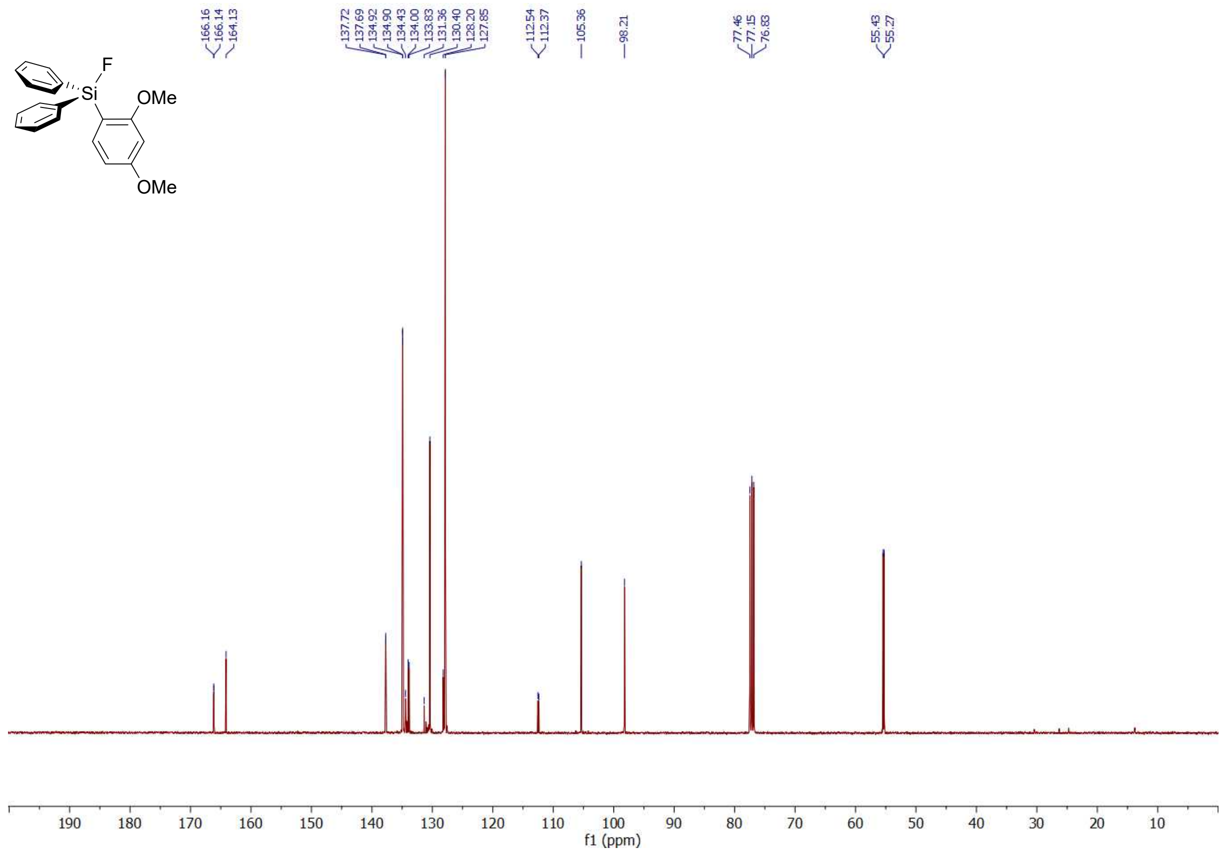

Figure S22: ^{13}C NMR spectrum of **6e** in $CDCl_3$

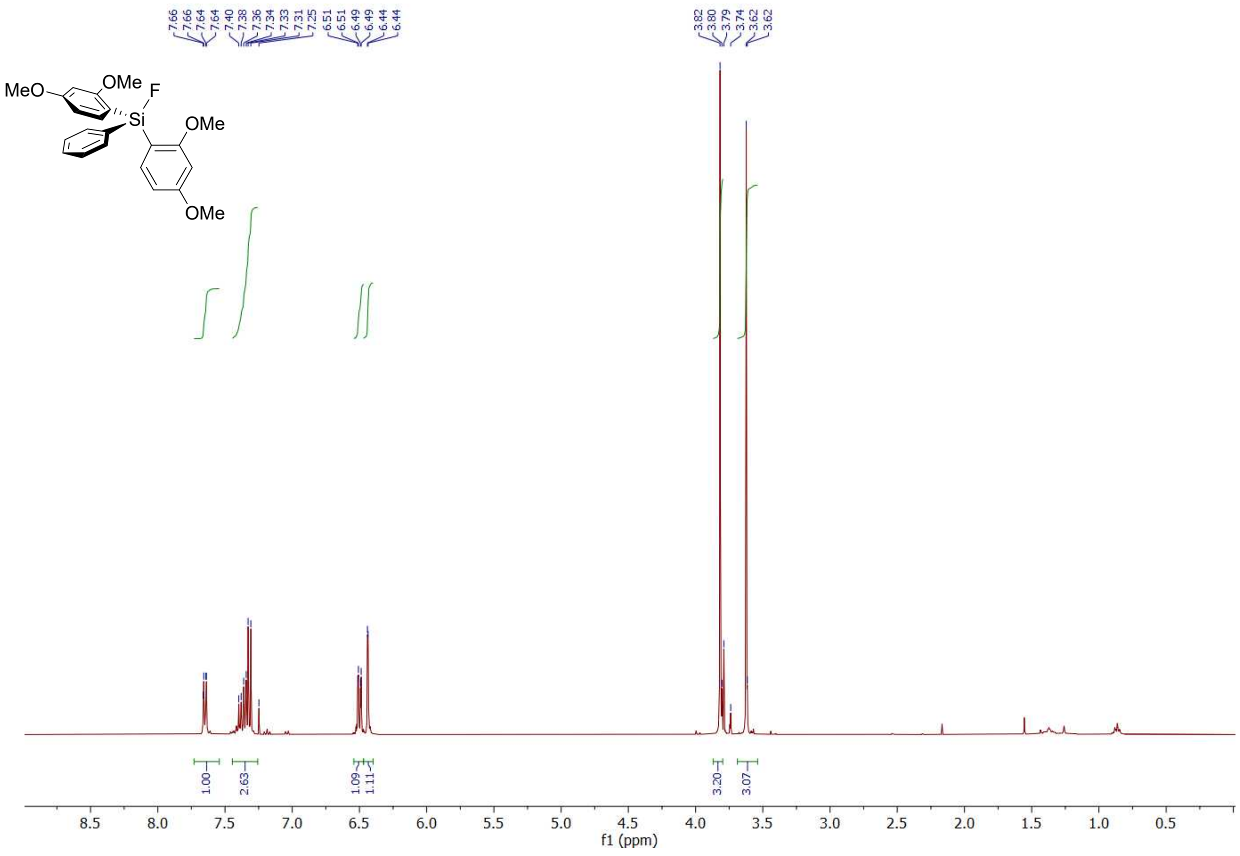

Figure S23: ^{1}H NMR spectrum of **6f** in $CDCl_3$

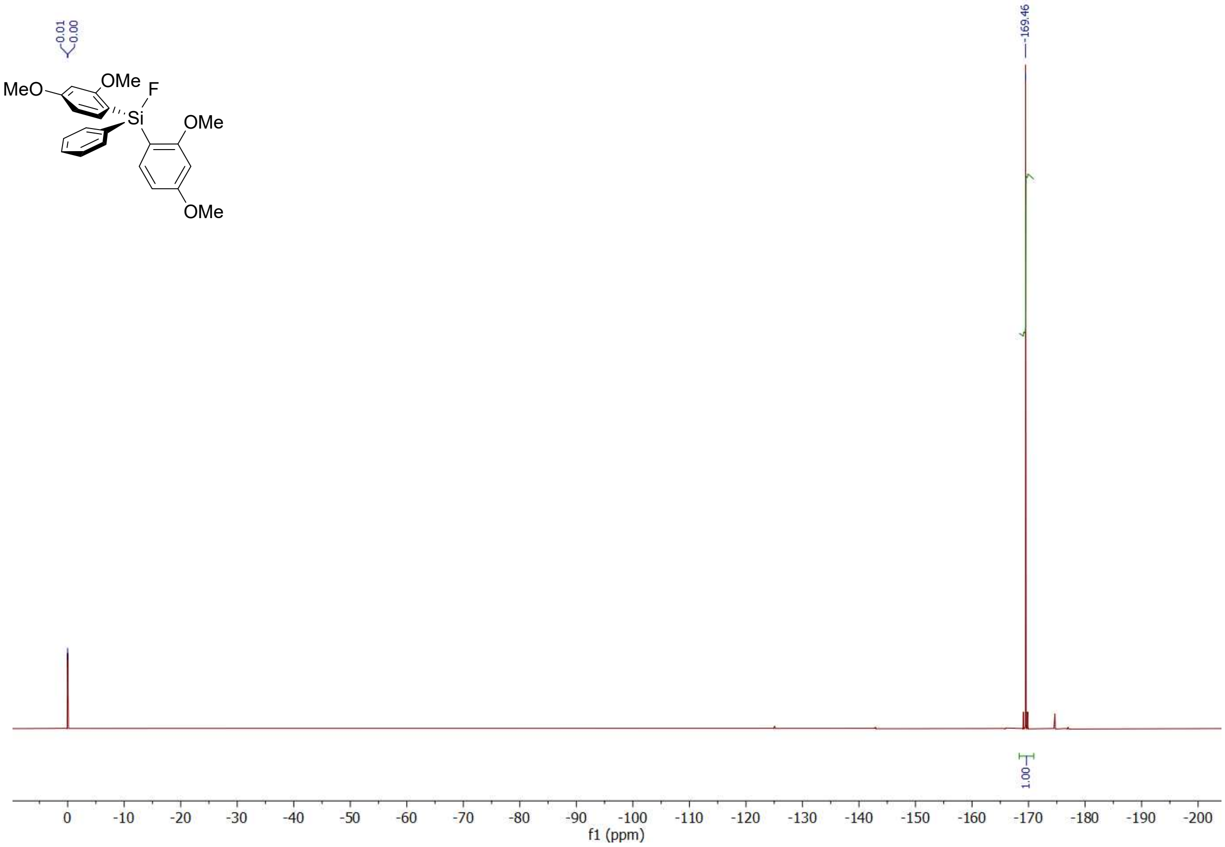

Figure S24: ^{19}F NMR spectrum of **6f** in $CDCl_3$

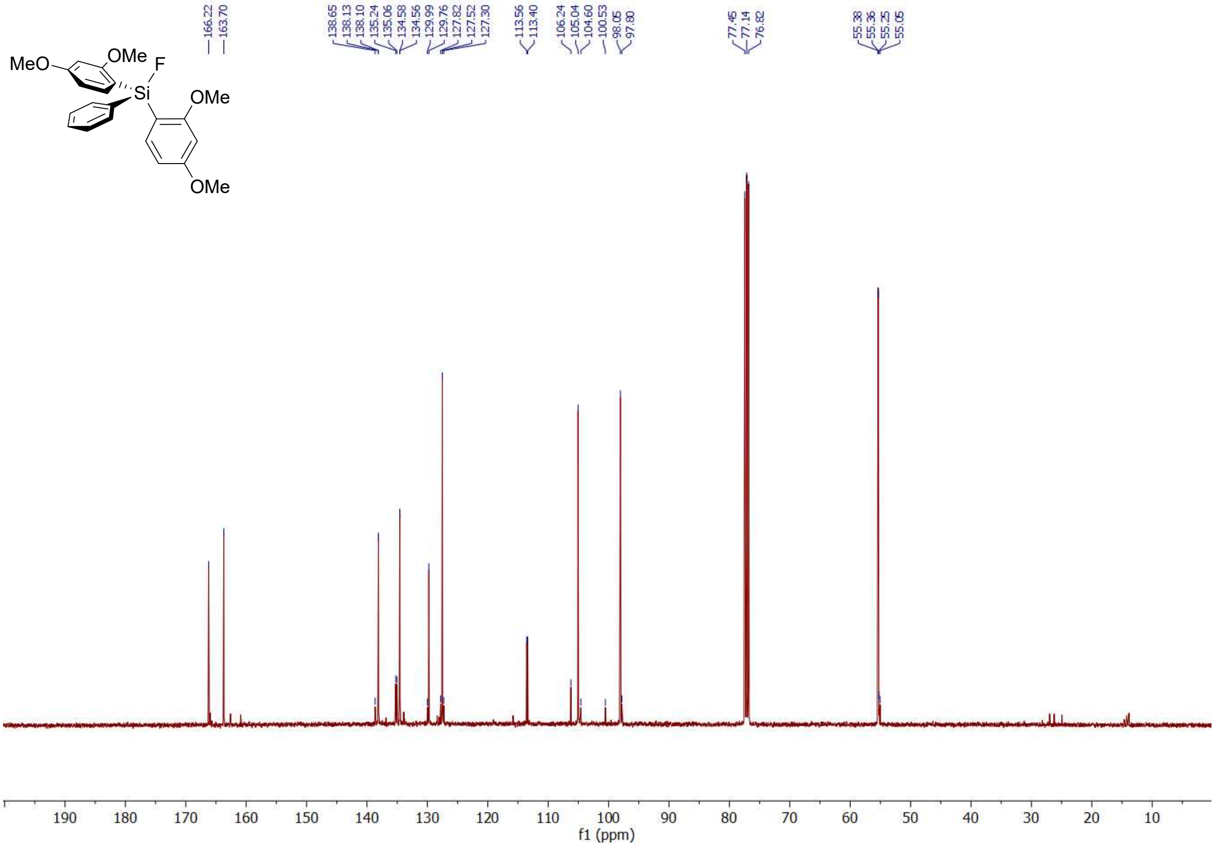

Figure S25: ^{13}C NMR spectrum of **6f** in $CDCl_3$

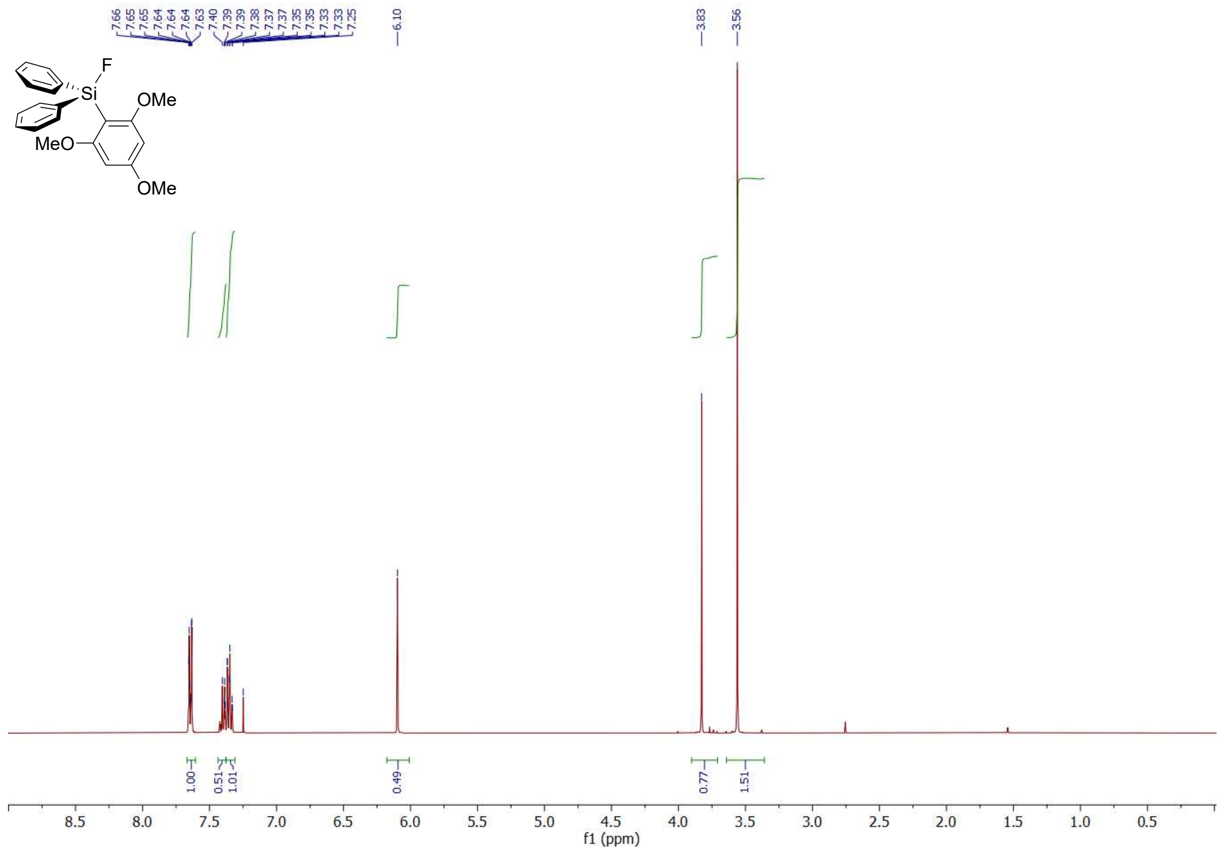

Figure S26: ^{1}H NMR spectrum of **6g** in $CDCl_3$

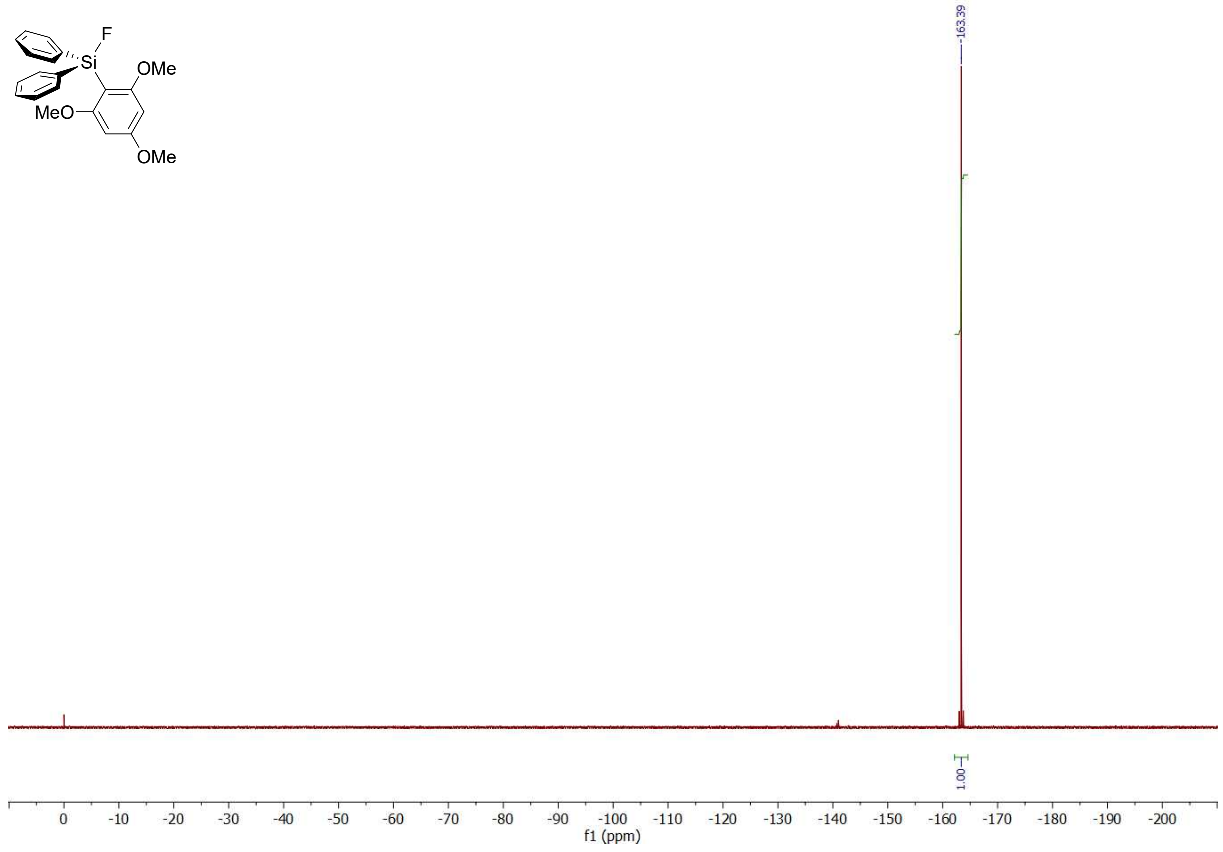

Figure S27: ^{19}F NMR spectrum of **6g** in $CDCl_3$

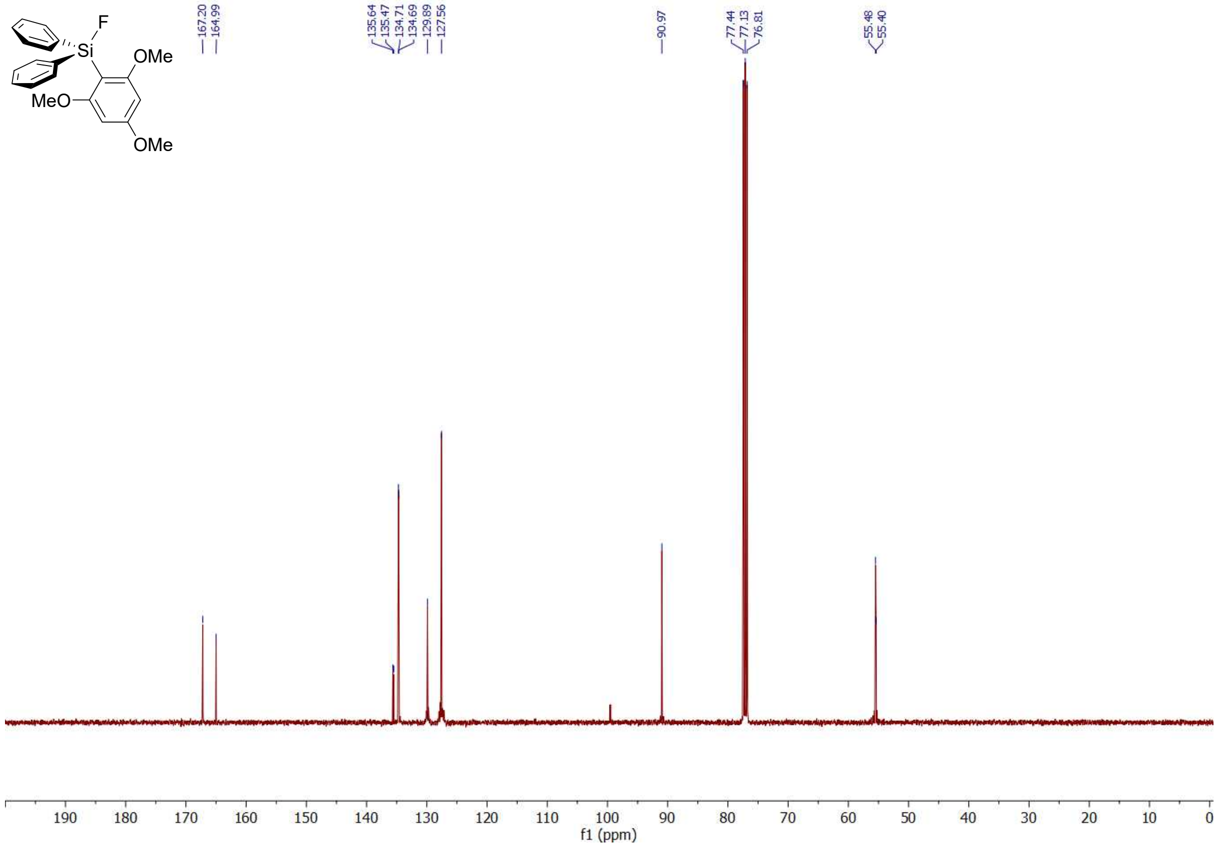

Figure S28: ^{13}C NMR spectrum of **6g** in $CDCl_3$

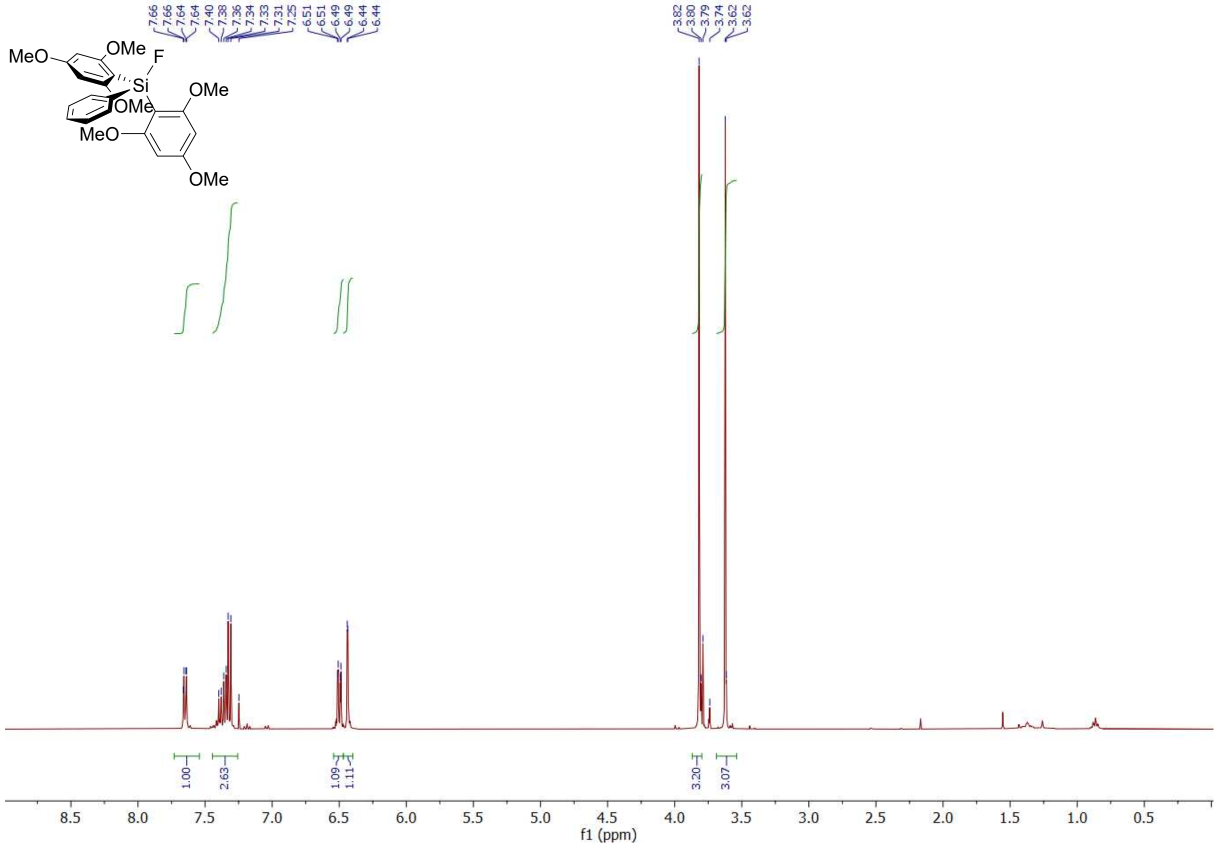

Figure S29: ^{1}H NMR spectrum of **6h** in $CDCl_3$

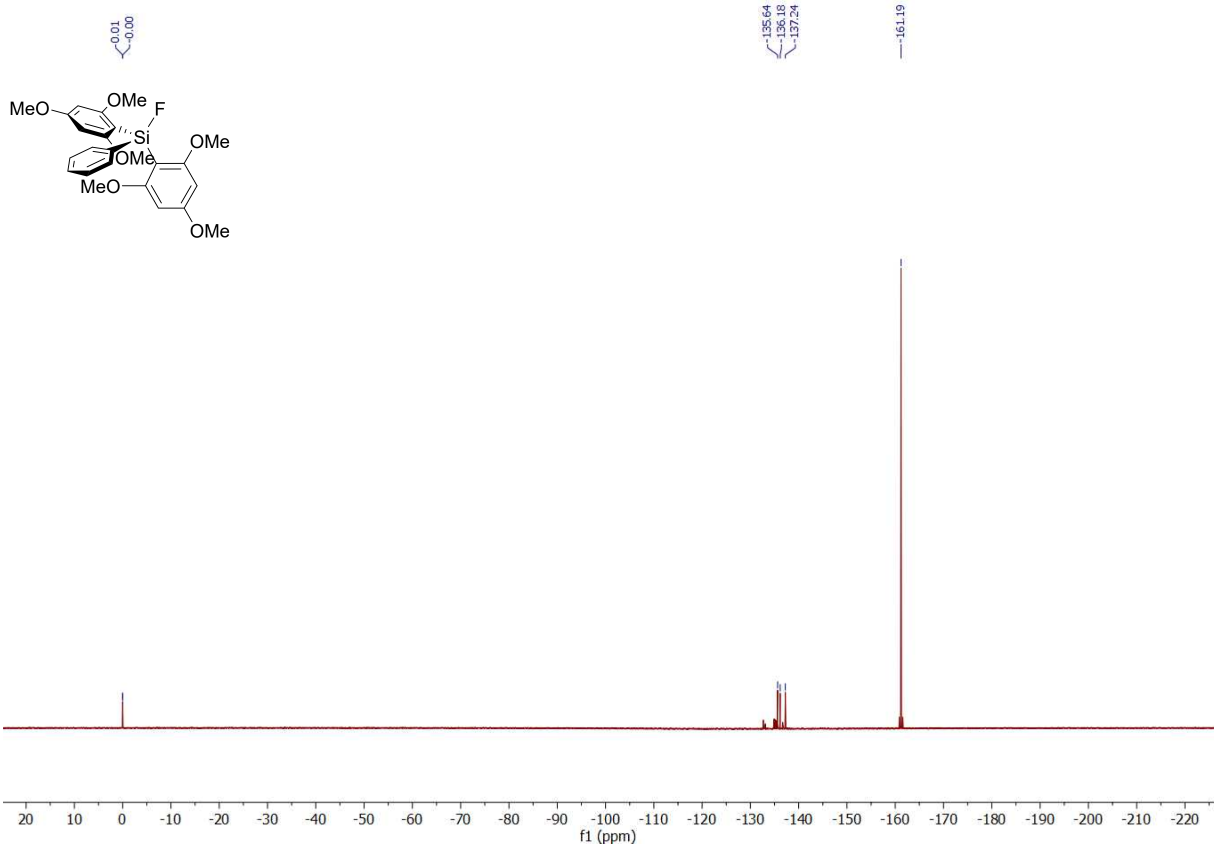

Figure S30: ^{19}F NMR spectrum of **6h** in $CDCl_3$

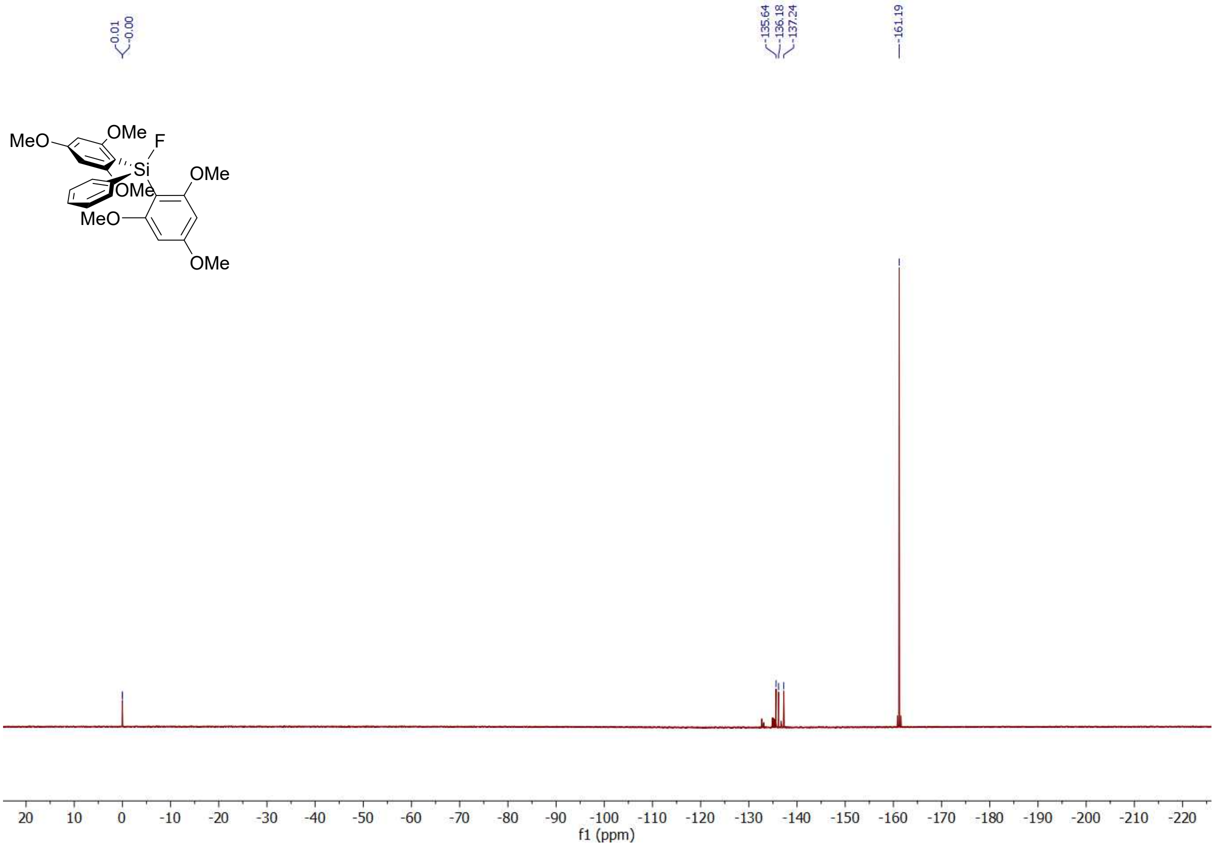

Figure S31: ^{13}C NMR spectrum of **6h** in $CDCl_3$

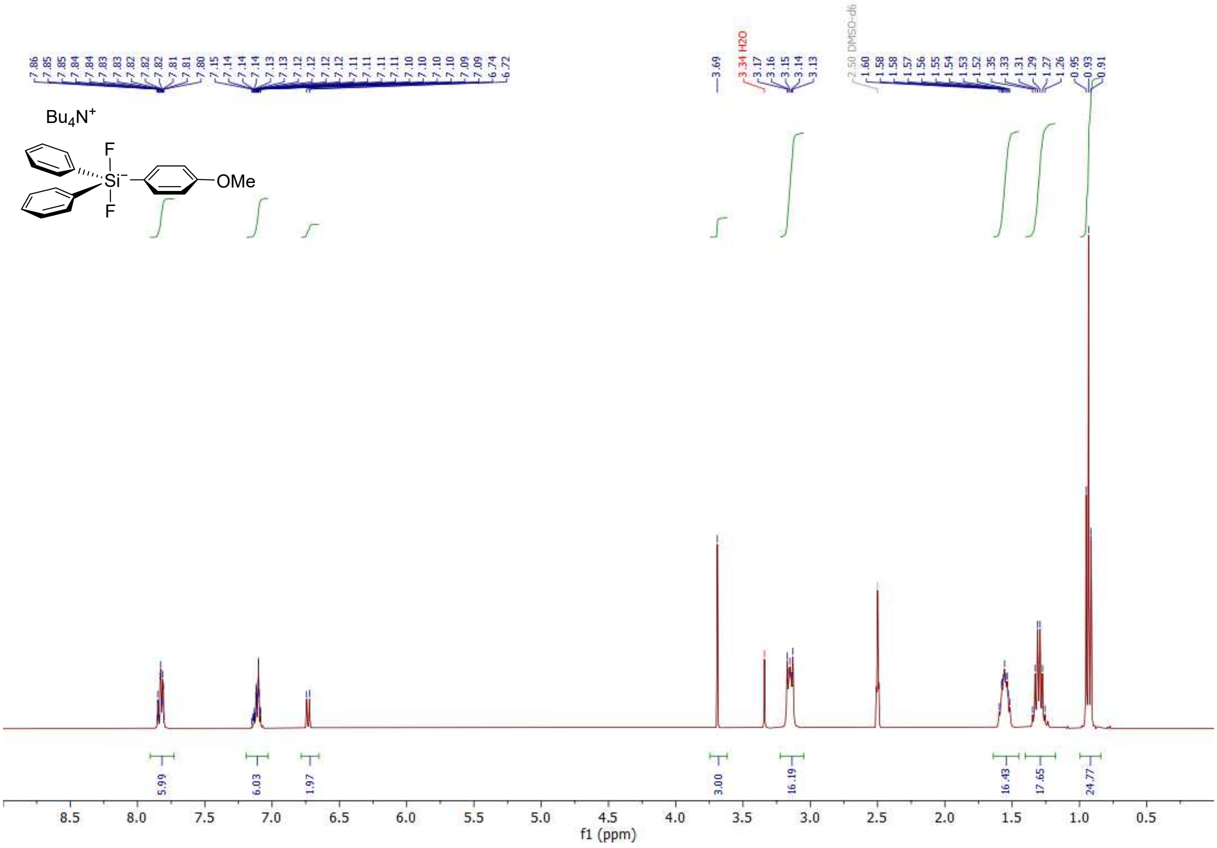

Figure S32: ^{1}H NMR spectrum of **4b** in DMSO-d_6

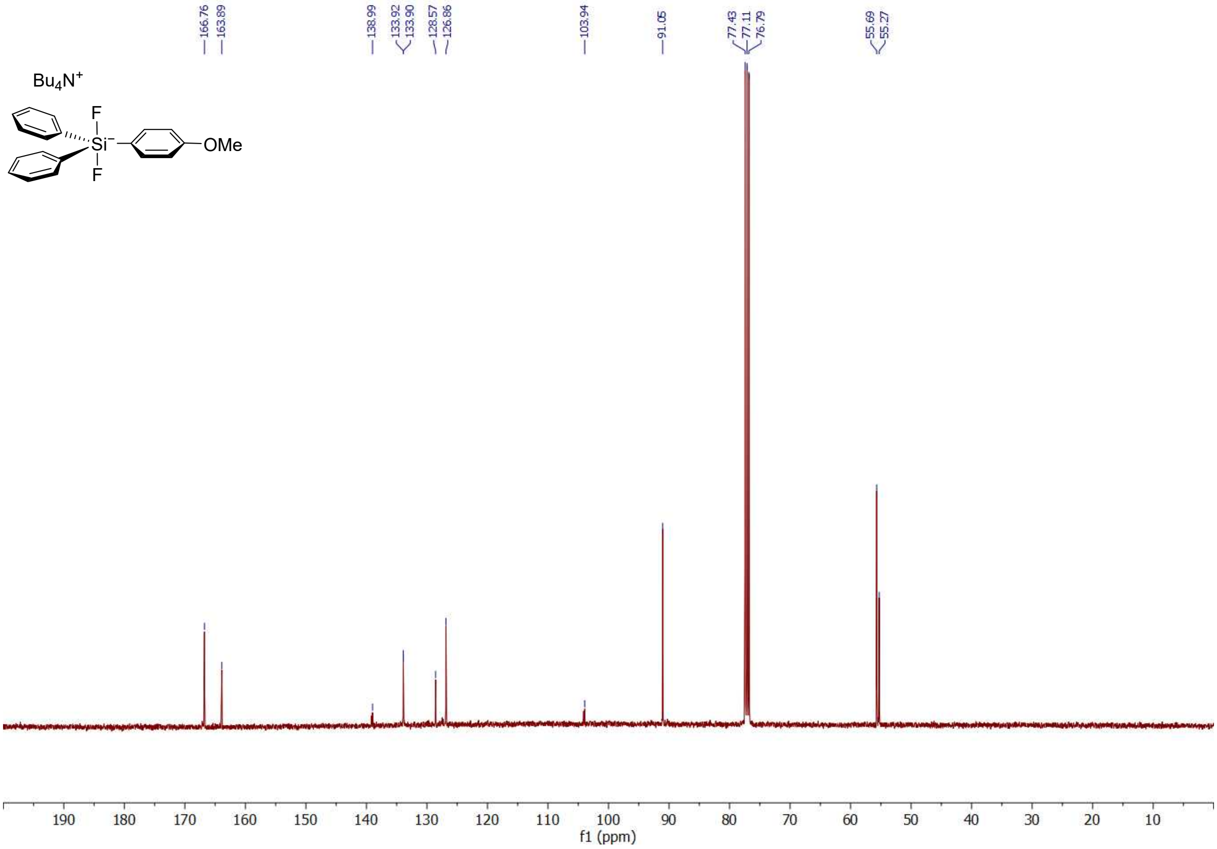

Figure S33: ^{13}C NMR spectrum of **4b** in DMSO-d_6

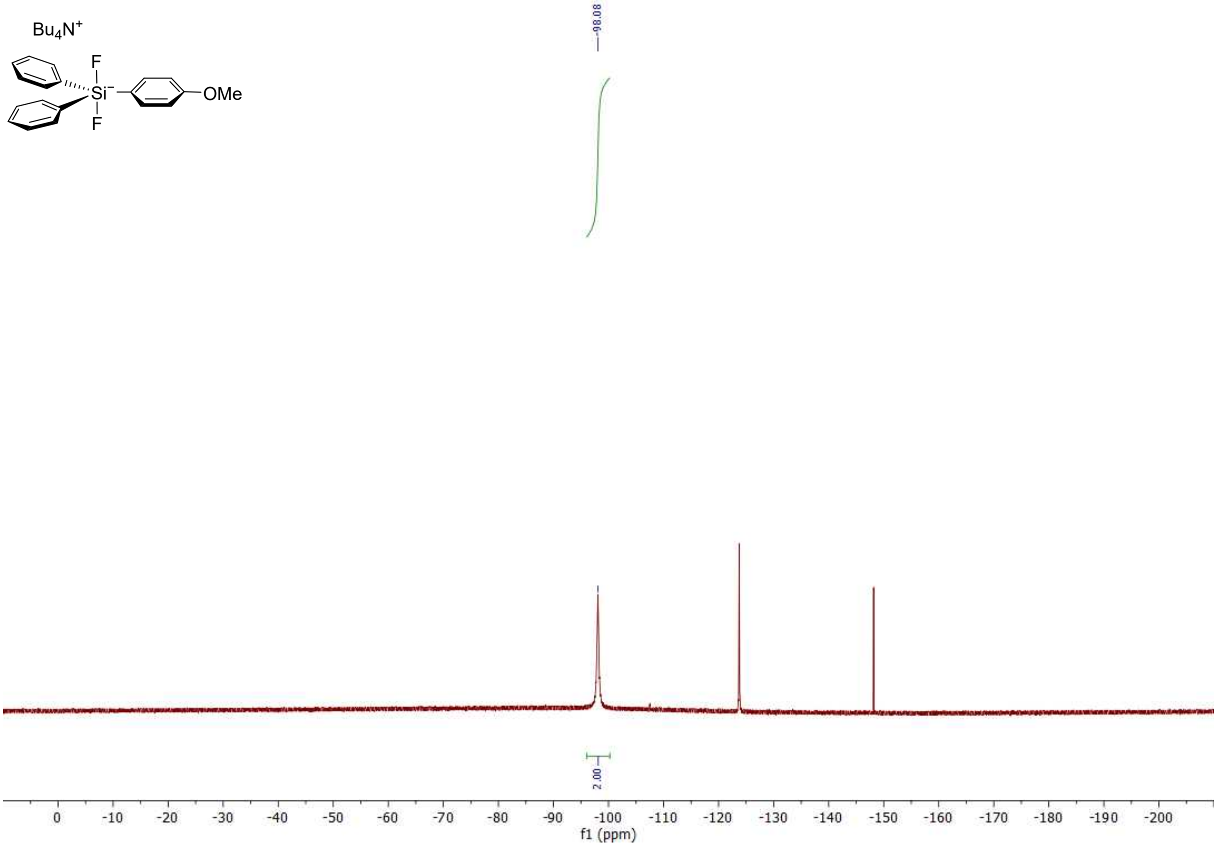

Figure S34: ^{19}F NMR spectrum of **4b** in DMSO-d_6

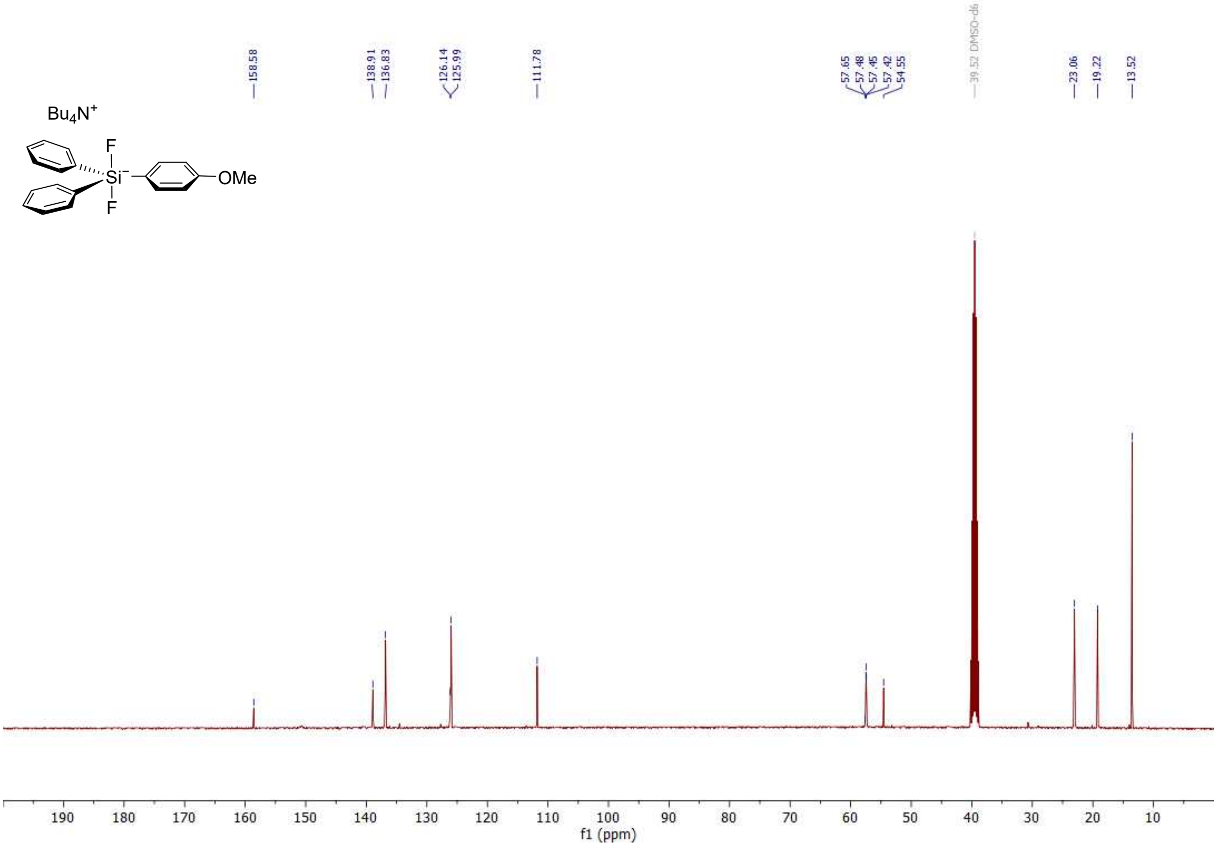

Figure S35: ^{13}C NMR spectrum of **4b** in DMSO-d_6

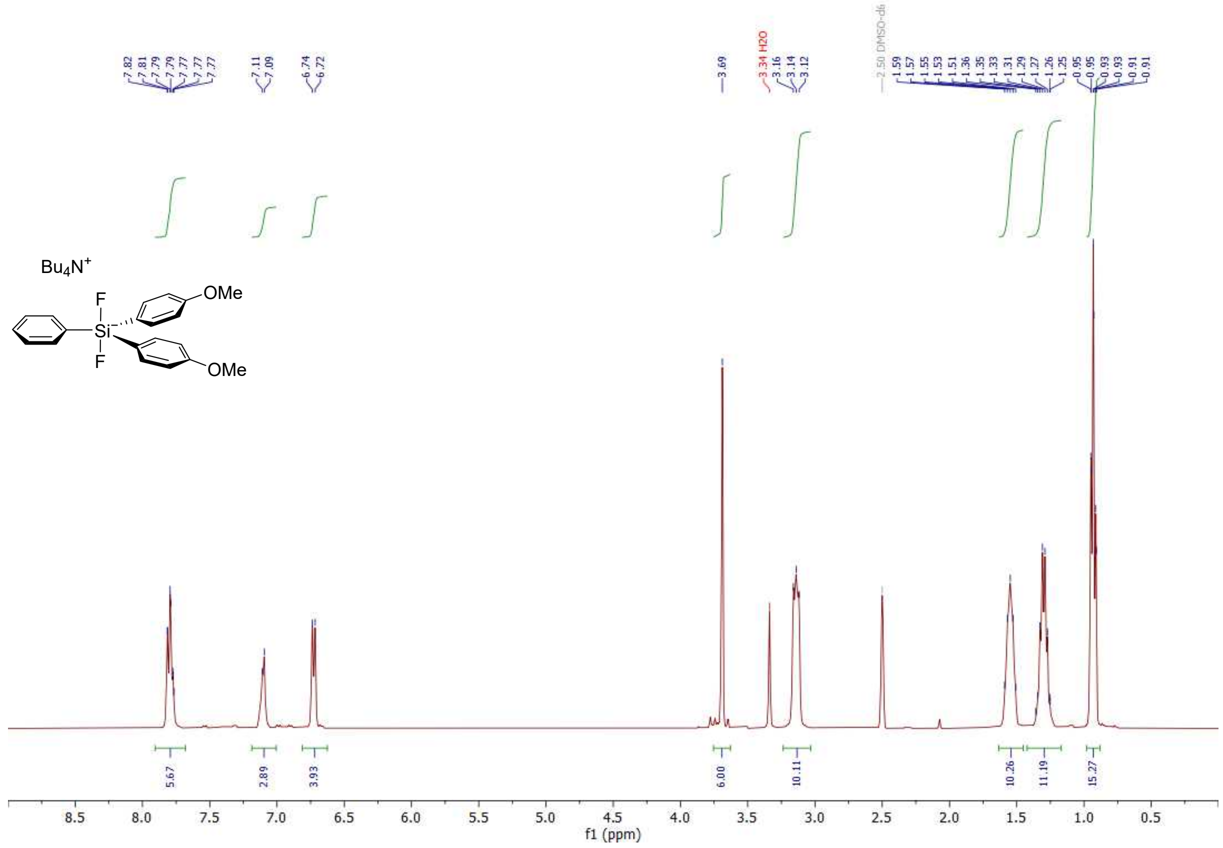

Figure S36: ^{1}H NMR spectrum of **4c** in DMSO-d_6

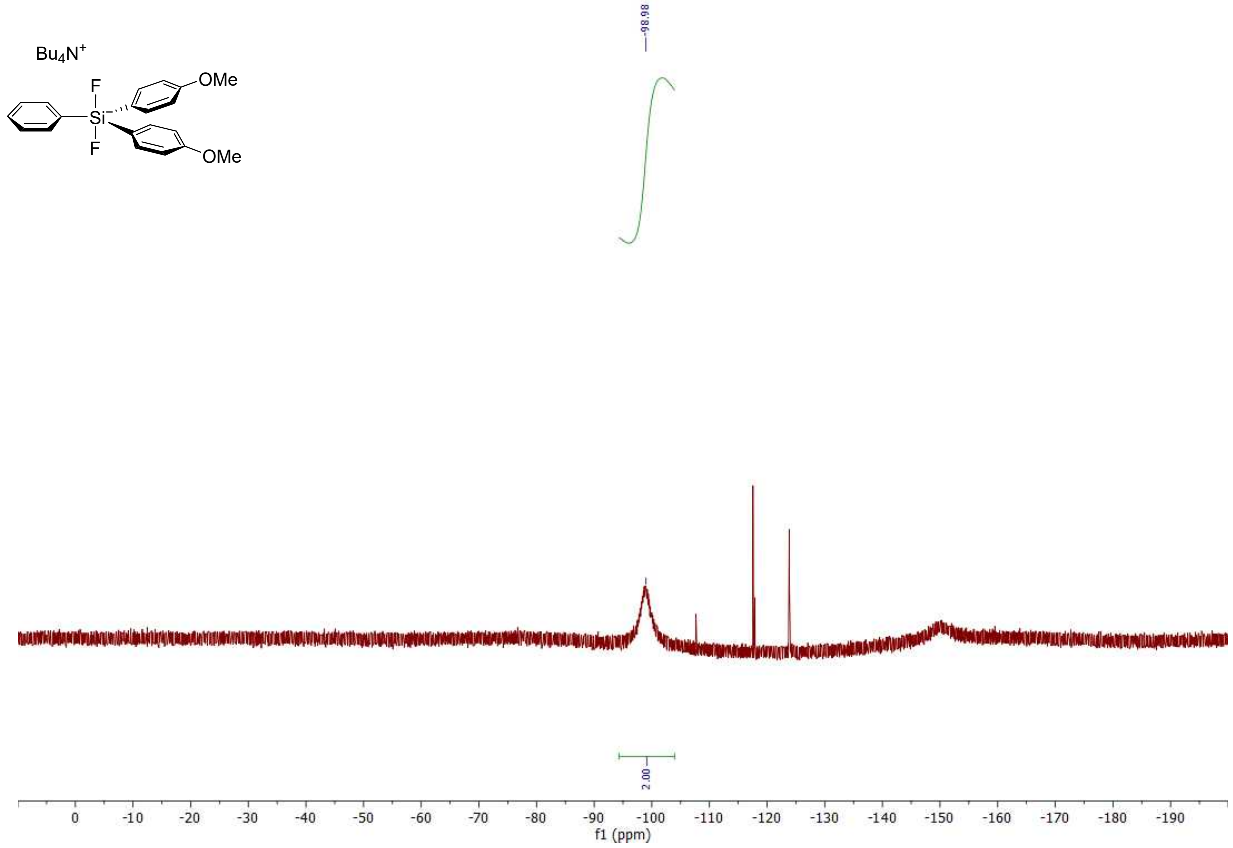

Figure S37 ^{19}F NMR spectrum of **4c** in DMSO-d_6

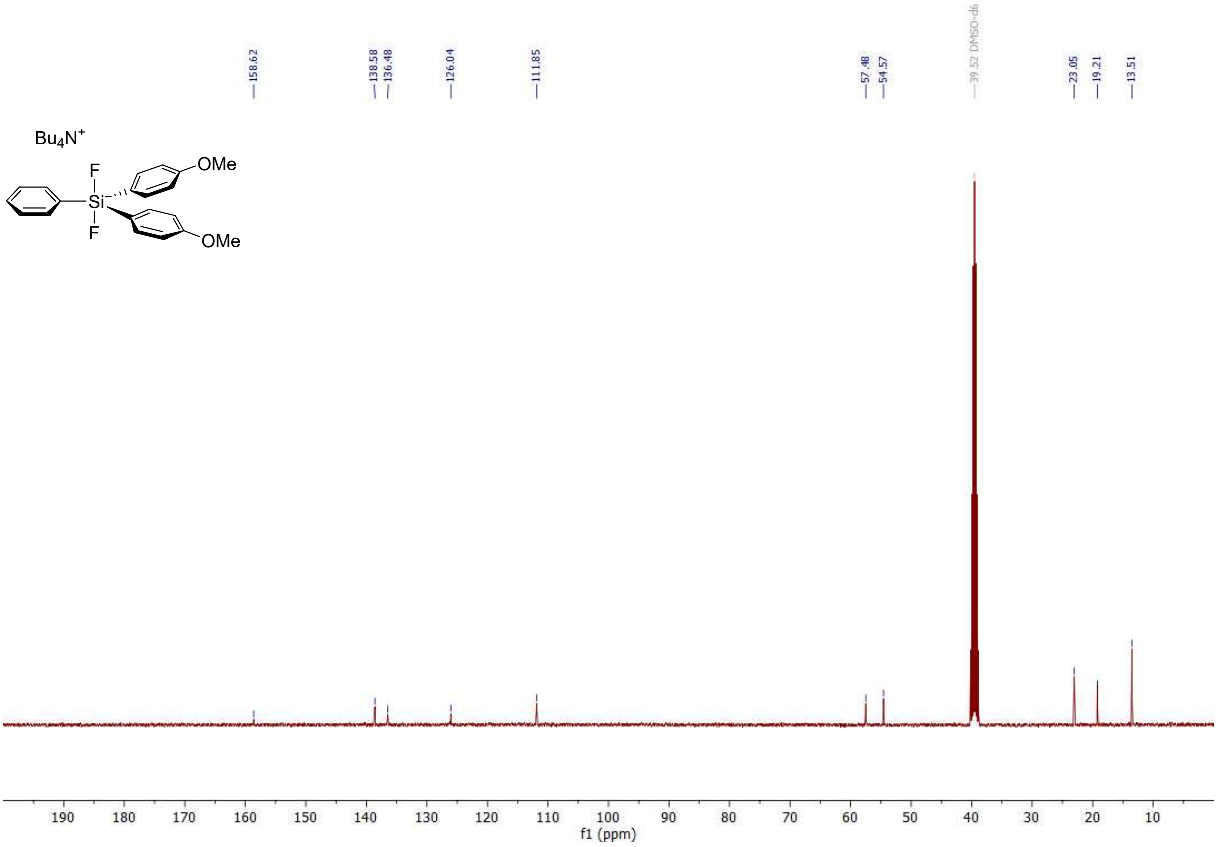

Figure S38: ^{13}C NMR spectrum of **4c** in DMSO-d_6

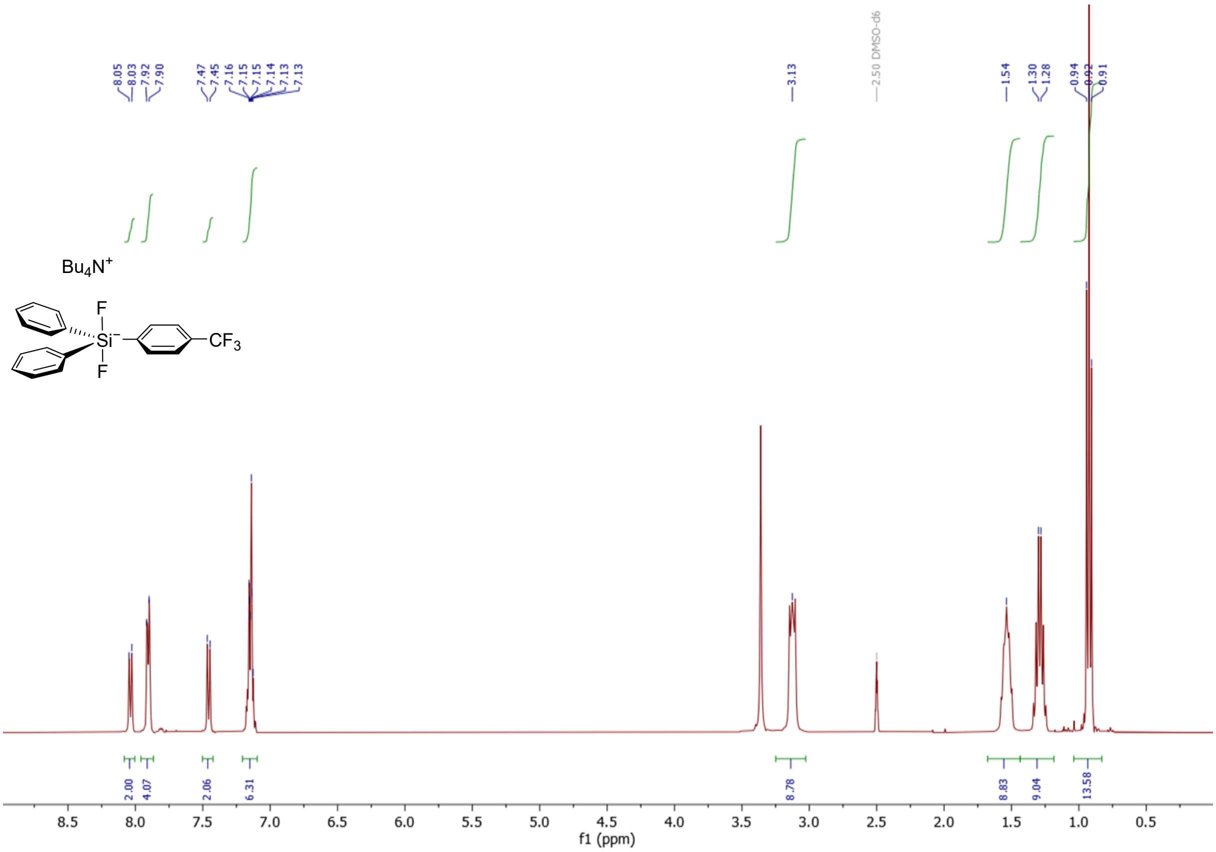

Figure S39: ^{1}H NMR spectrum of **4d** in DMSO-d_6

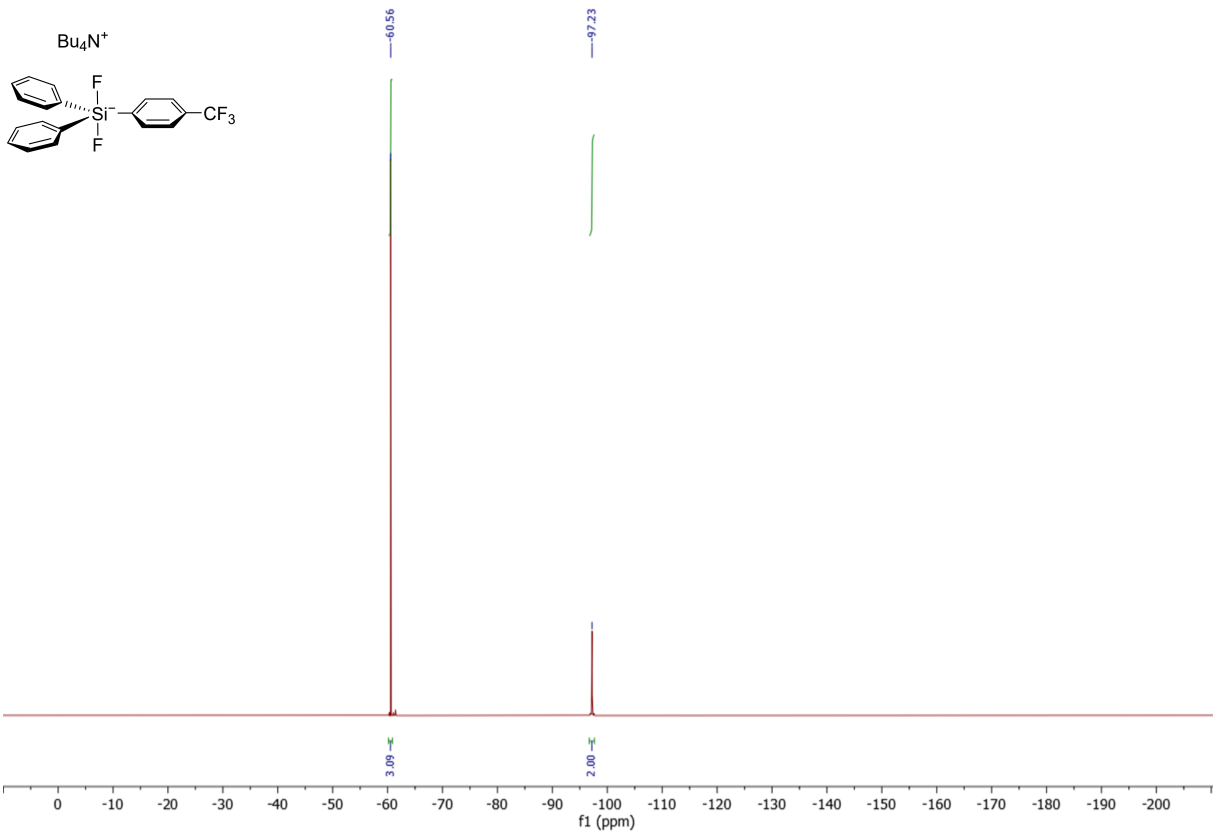

Figure S40: ^{19}F NMR spectrum of **4d** in DMSO-d_6

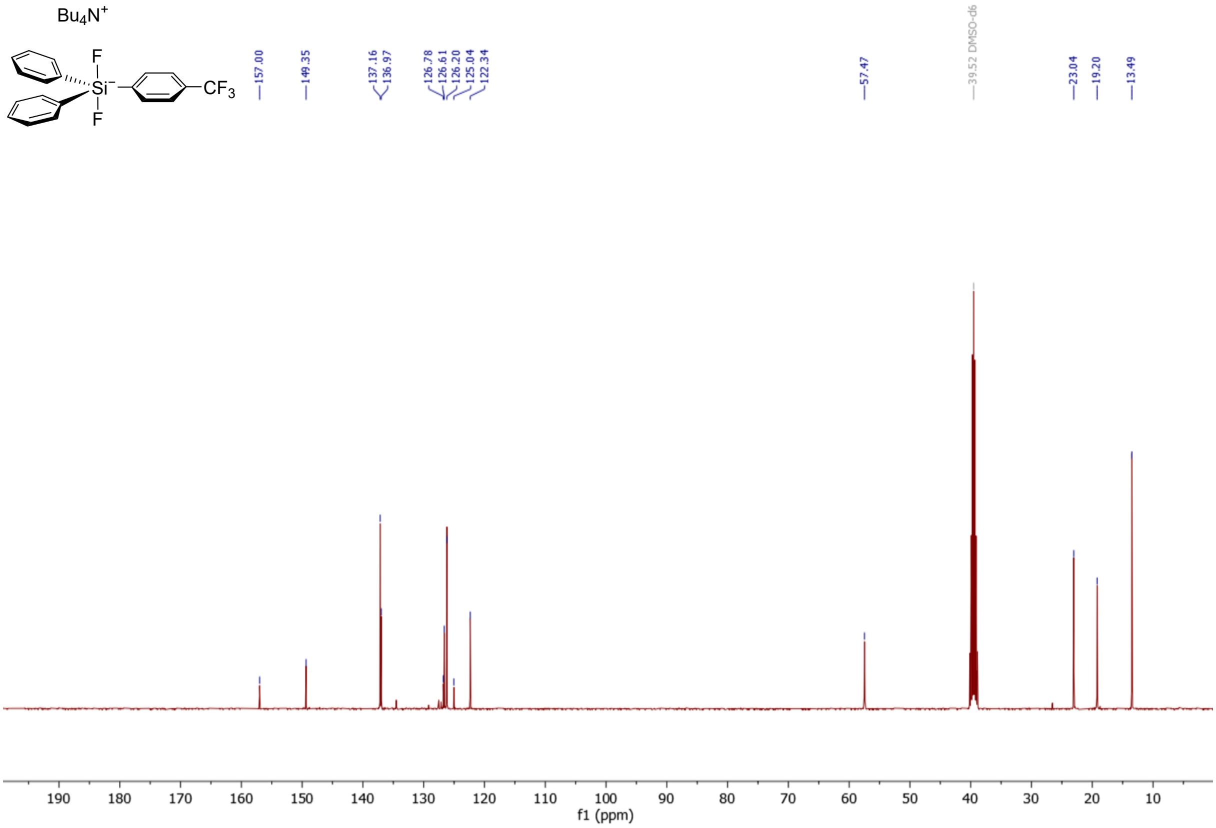

Figure S41: ^{13}C NMR spectrum of **4d** in DMSO-d_6

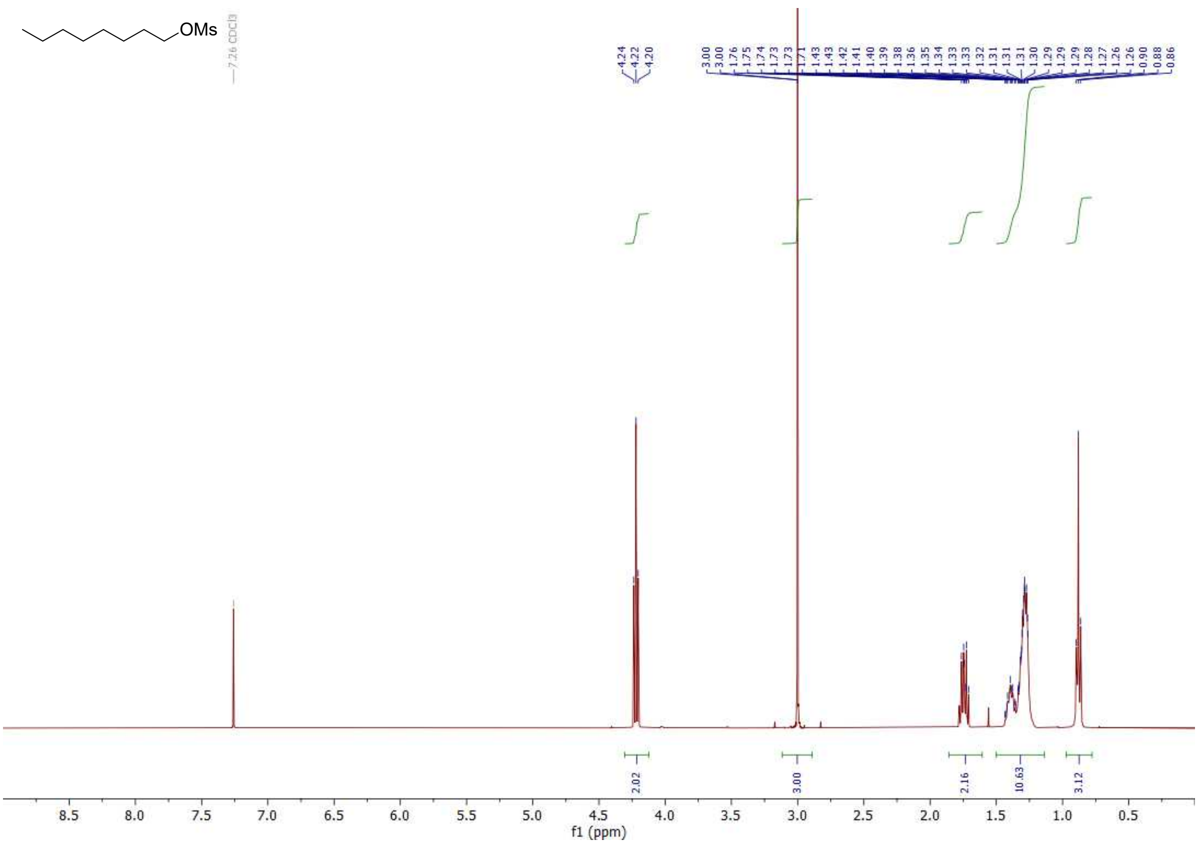

Figure S42: ^{1}H NMR spectrum of **13b** in $CDCl_3$

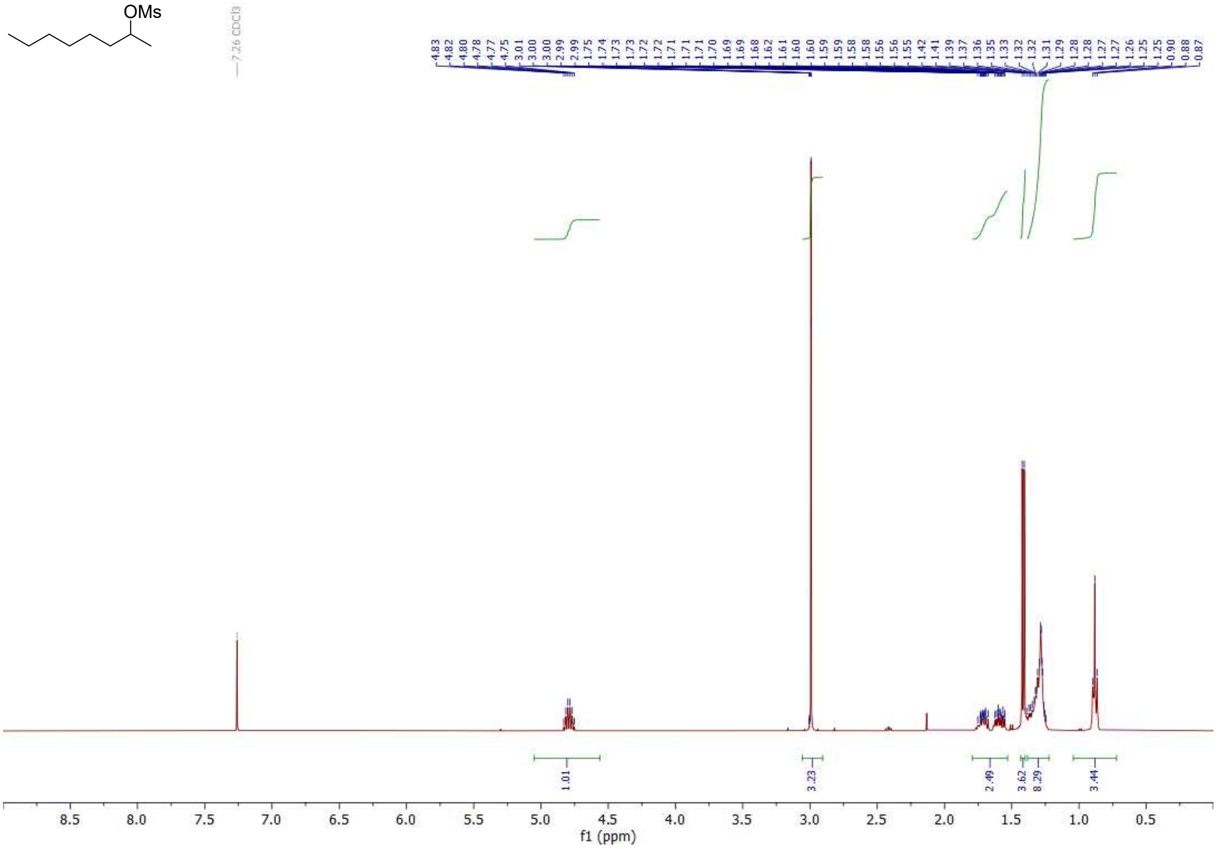

Figure S43: ^{1}H NMR spectrum of **2b** in $CDCl_3$

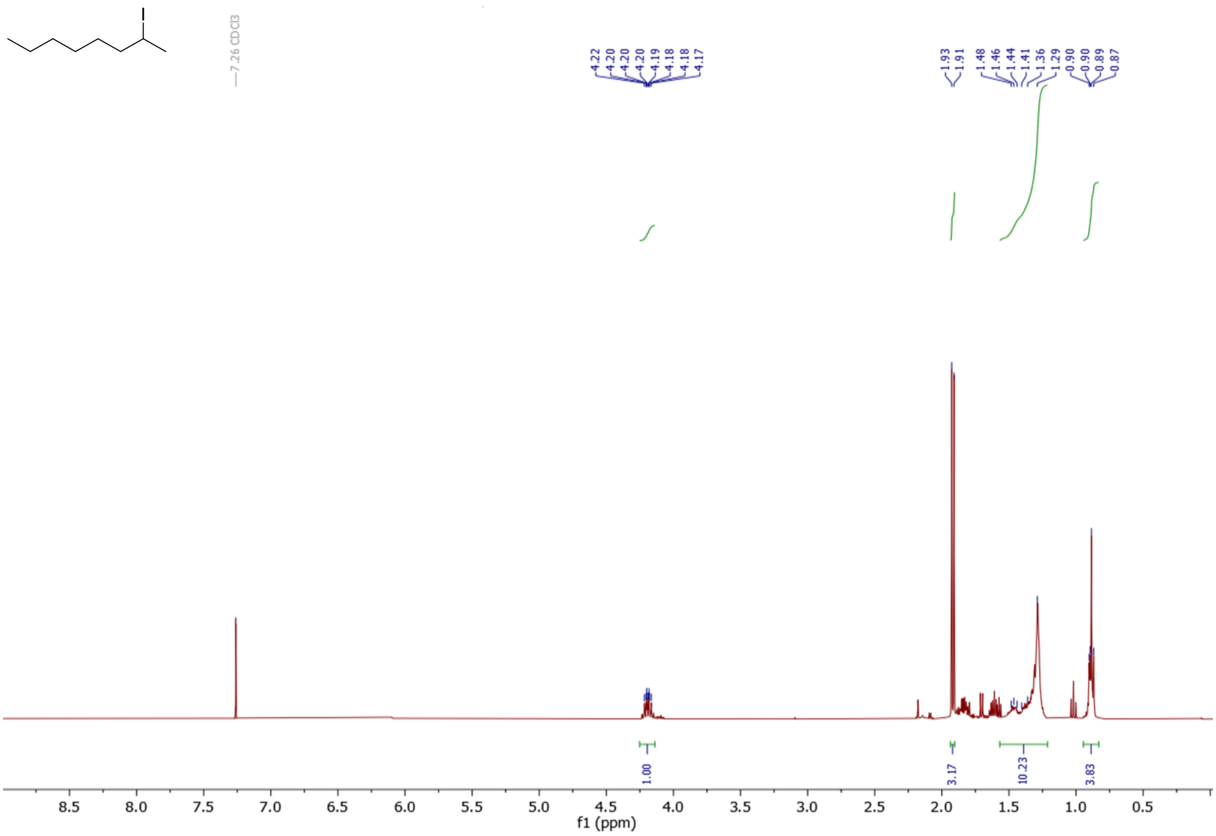

Figure S44: ^{1}H NMR spectrum of **2c** in $CDCl_3$

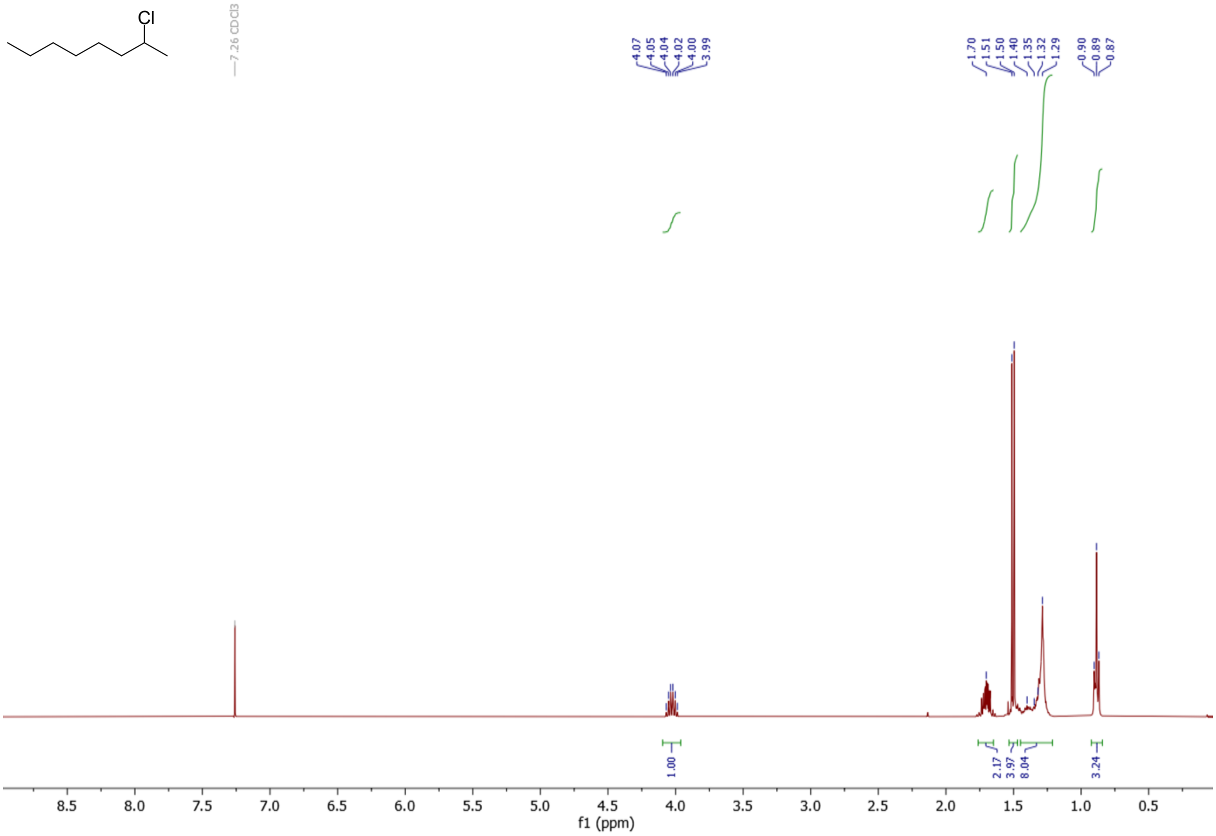

Figure S45: ^{1}H NMR spectrum of **2d** in $CDCl_3$

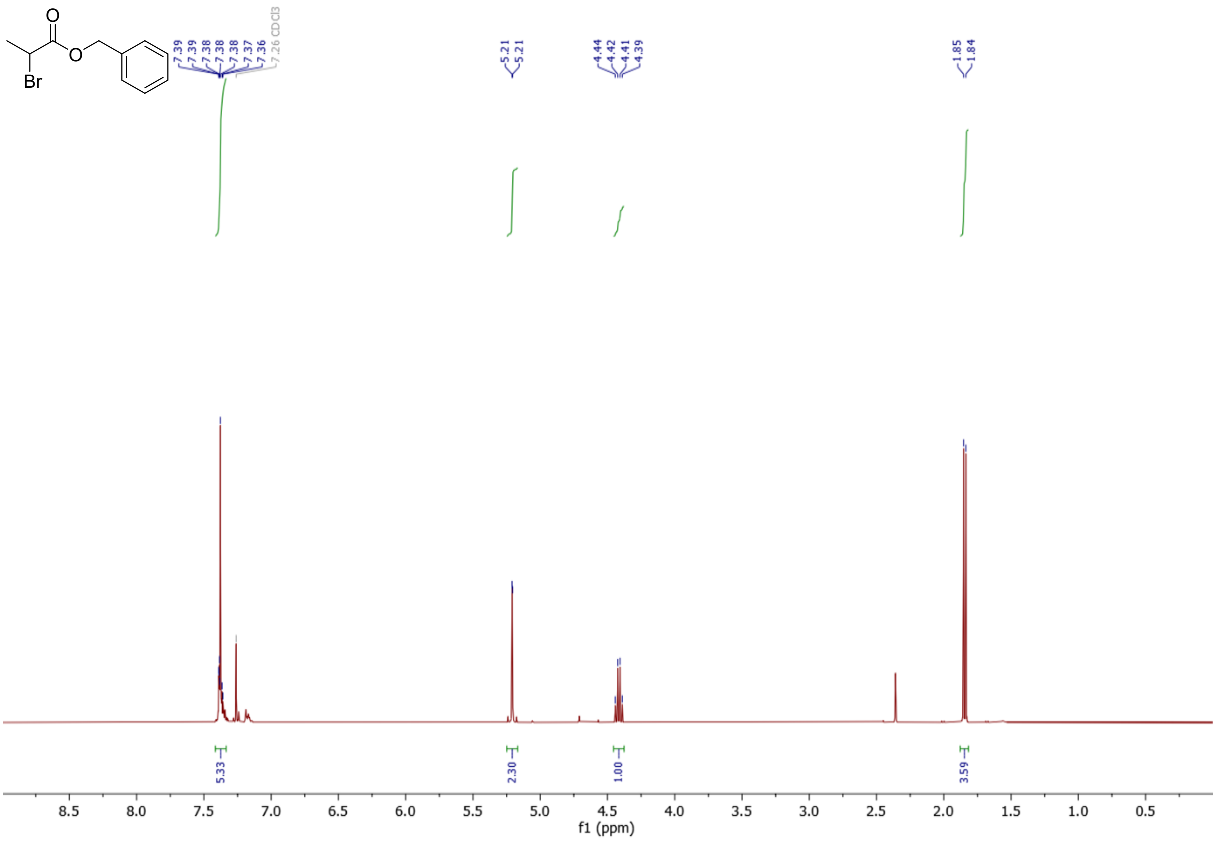

Figure S46: ^{1}H NMR spectrum of benzyl 2-bromopropionate in $CDCl_3$

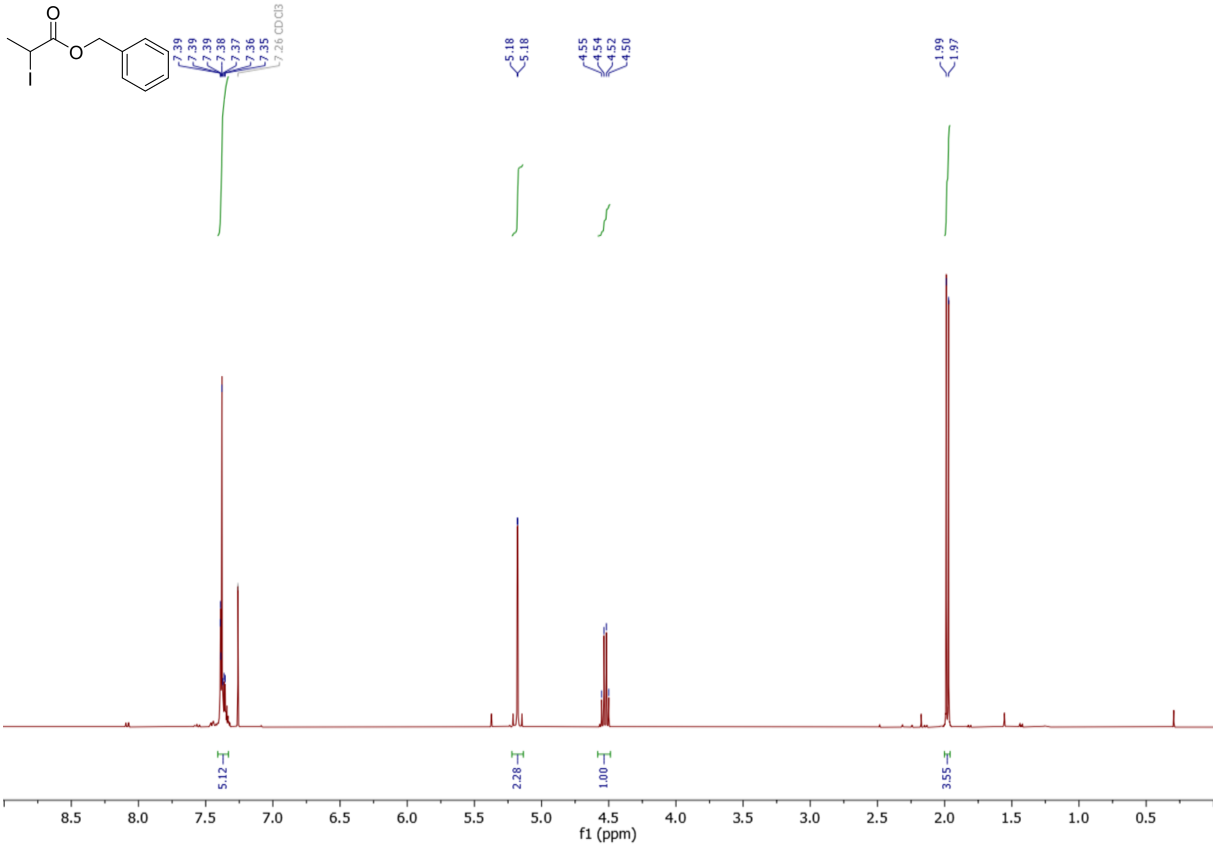

Figure S47: ^{1}H NMR spectrum of **15** in $CDCl_3$

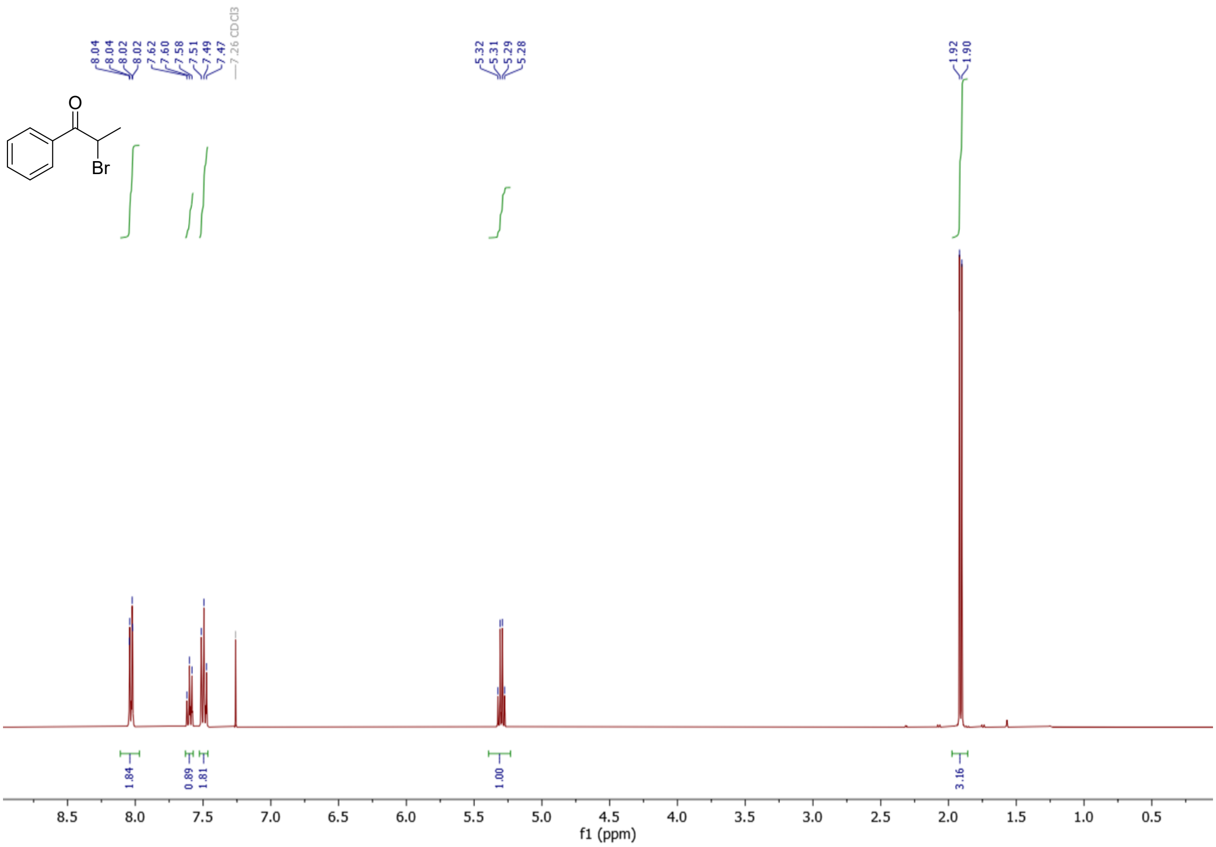

Figure S48: ^{1}H NMR spectrum of 2-bromo-1-phenylpropan-1-one in $CDCl_3$

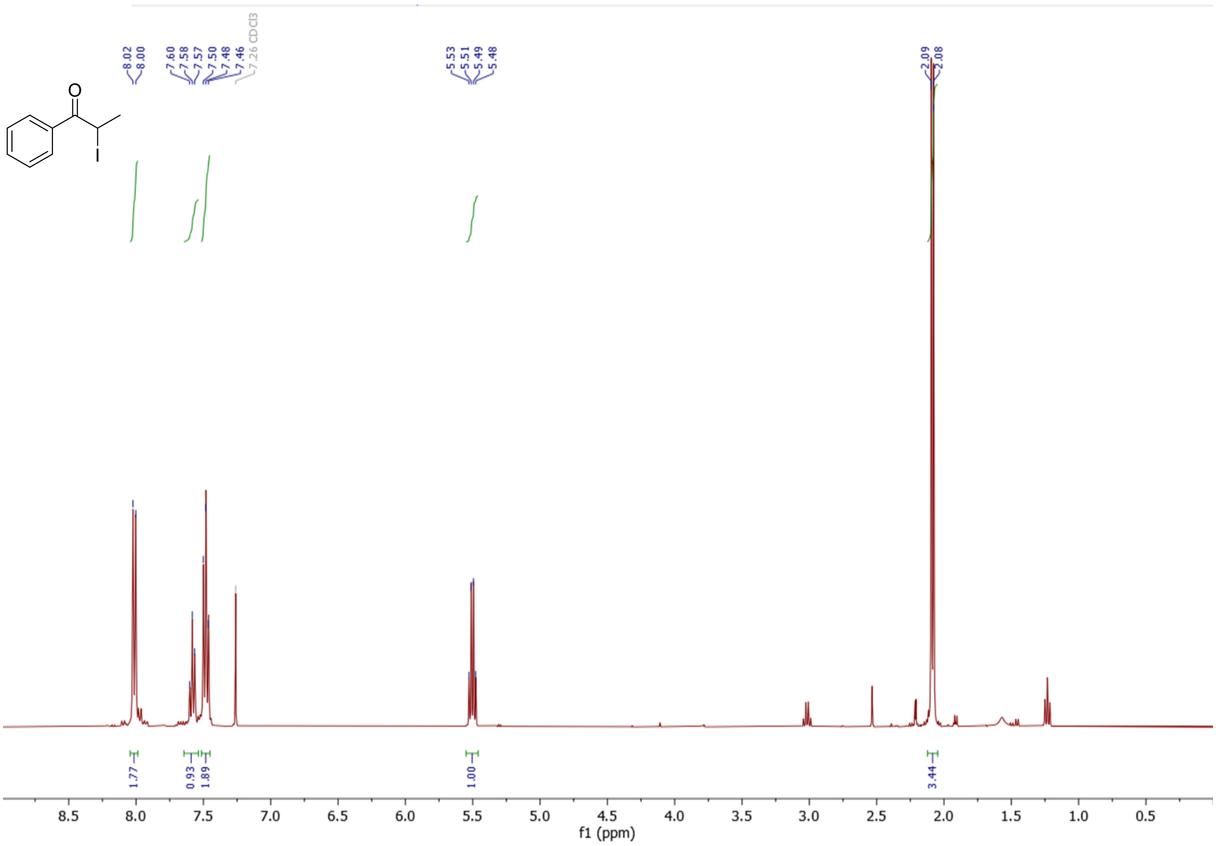

Figure S49: ^{1}H NMR spectrum of **16** in $CDCl_3$

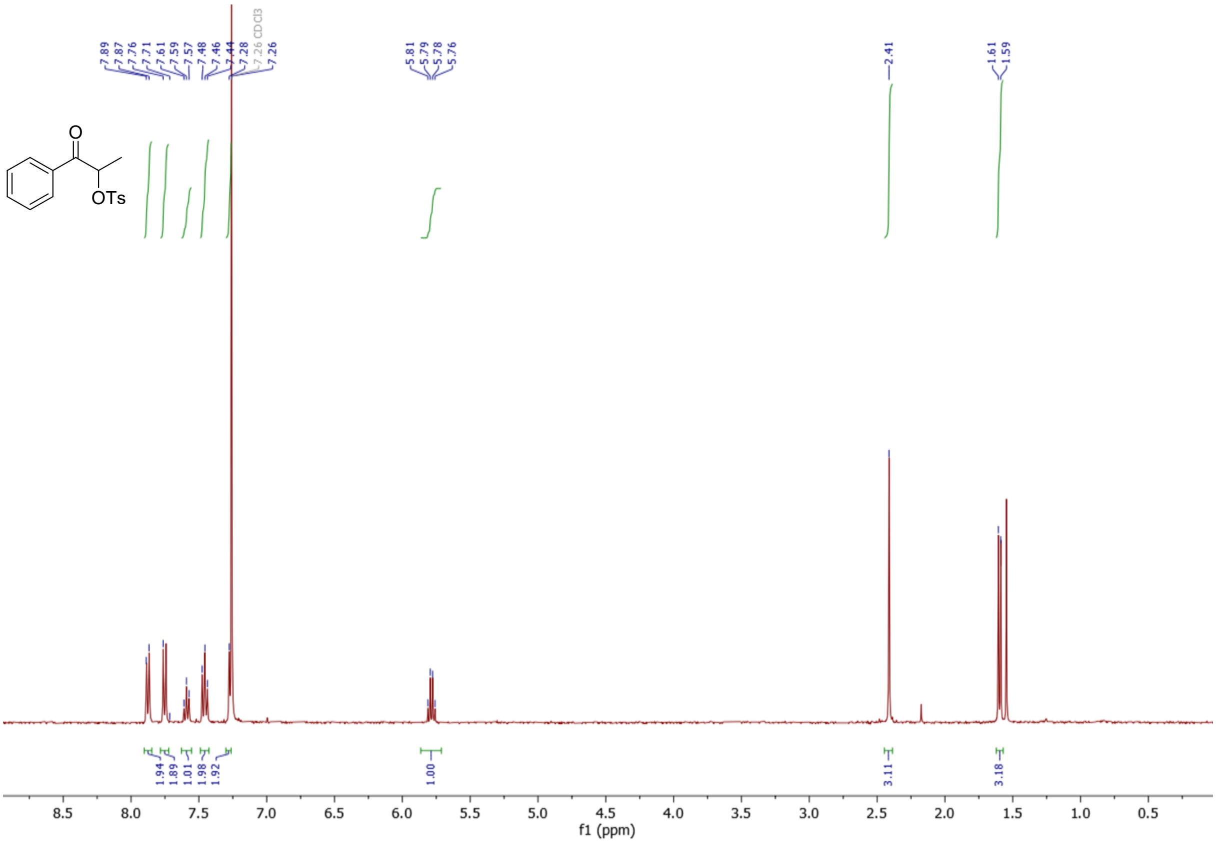

Figure S50: ^{1}H NMR spectrum of **17** in $CDCl_3$

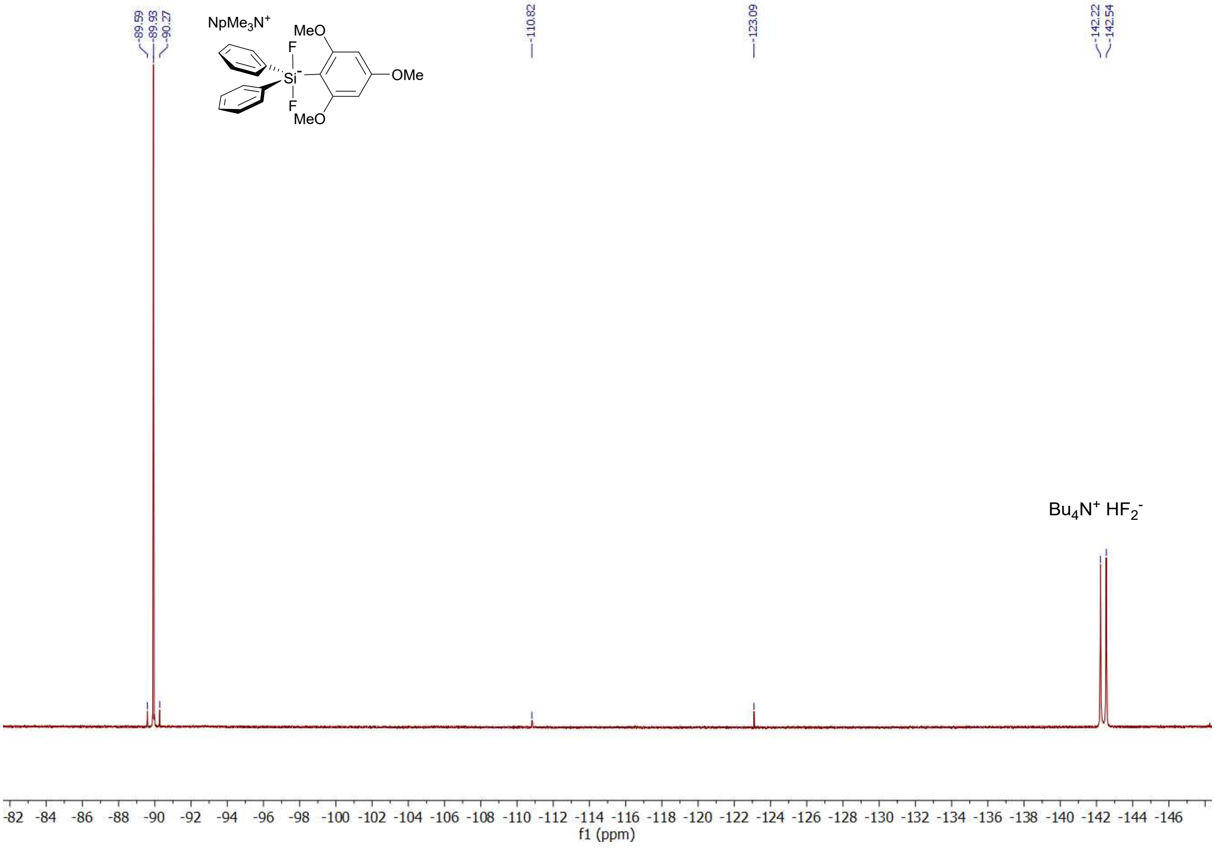

Figure S51: ^{19}F NMR spectrum of partially decomposed $NpMe_3N^+(2,4,6\text{-triMeOC}_6H_2)Ph_2Si^-F_2$ (**4h**) in toluene

4. Crystallographic data of difluorosilicates 4b-4d

The X-ray data were collected on a Bruker D8 VENTURE system equipped with charge-integrating pixel array detector Photon II 7, a multilayer monochromator and an Incoatec microfocus sealed tube using combined φ and ω scans at 180 K. The positional and anisotropic thermal parameters of all non-hydrogen atoms were refined. All hydrogen atoms were located in a difference map, but repositioned geometrically, then they were initially refined with soft restraints on the bond lengths and angles to regularise their geometry (C-H in the range of 0.93-0.98 Å) and U_{iso}(H) (in the range 1.2-1.5 times U_{eq} of the parent atom), after which the positions of the hydrogen atoms were refined with riding constraints.

Tetrabutylammonium difluoro(4-methoxyphenyl)diphenylsilicate (4b): CCDC 2312704
CuK_{α} radiation (λ = 1.54178 Å) was used for the data collection. The refinement of 361 parameters on all 6188 reflections (5558 observed reflections, $I > 2\ \sigma(I)$) converged to the final R_1 of 0.0393 and R_{w1} of 0.1090. Plate-like colorless crystal, dimensions 0.140 mm x 0.221 mm x 0.392 mm.

Tetrabutylammonium difluorobis(4-methoxyphenyl)phenylsilicate (4c): CCDC 2312705
CuK_{α} radiation (λ = 1.54178 Å) was used for the data collection. The refinement of 379 parameters on all 6424 reflections (5119 observed reflections, $I > 2\ \sigma(I)$) converged to the final R_1 of 0.0800 and R_{w1} of 0.2223. Needle-like colorless crystal, dimensions 0.074 mm x 0.184 mm x 0.544 mm.

Tetrabutylammonium difluorodiphenyl[(4-trifluoromethyl)phenyl]silicate (4d): CCDC 2312706
MoK_{α} radiation (λ = 0.71073 Å) was used for the data collection. The refinement of 380 parameters on all 7320 reflections (6034 observed reflections, $I > 2\ \sigma(I)$) converged to the final R_1 of 0.0458 and R_{w1} of 0.0973. Needle-like colorless crystal, dimensions 0.197 mm x 0.199 mm x 0.255 mm.

Data collection: APEX4[1]; Unit Cell refinement and data reduction: SAINT[23]; program used to solve structure: SIR92[24] ; program used to refine structure: CRYSTALS[25]. CCDC 2312704-2312706 contain the supplementary crystallographic data for this paper. These data can be obtained free of charge from The Cambridge Crystallographic Data Centre via www.ccdc.cam.ac.uk/structures.

5. XYZ files of computed structures (energies in kJ/mol)

Preliminary computations using Gaussian 16, method SMD(MeCN)-M06L/def2-SVPD

18aA: ΔG = -3669683.40 kJ/mol

Si	0.99200	-0.05400	0.40500
C	0.15100	1.63500	0.67400
C	-0.76800	1.83200	1.71700
C	0.39400	2.71600	-0.18900
C	-1.43900	3.04600	1.87700
H	-0.97500	1.01700	2.41700
C	-0.24900	3.94300	-0.01800
H	1.09500	2.59700	-1.02100
C	-1.17700	4.10900	1.01200
H	-2.16500	3.16600	2.68400
H	-0.03300	4.77200	-0.69600
H	-1.69400	5.06300	1.13900
F	1.60400	-0.01500	2.08100
F	0.36100	-0.05800	-1.28400
C	2.77200	-0.12100	-0.27200
C	3.77600	0.71900	0.23900
C	3.13400	-1.01100	-1.29700
C	5.08000	0.68200	-0.25800
H	3.53600	1.42000	1.04300
C	4.44200	-1.07600	-1.77900
H	2.37700	-1.67000	-1.73100
C	5.42000	-0.22300	-1.26500
H	5.83700	1.35700	0.14700
H	4.70000	-1.78900	-2.56500
H	6.44200	-0.26200	-1.64800
C	0.01200	-1.65600	0.77000
C	-0.28500	-2.58900	-0.23900
C	-0.50000	-1.91900	2.05400
C	-1.05700	-3.72400	0.01600
H	0.08500	-2.42000	-1.25100
C	-1.29800	-3.03500	2.31100
H	-0.27700	-1.22900	2.87000
C	-1.57800	-3.94500	1.29100
H	-1.26400	-4.43400	-0.78900
H	-1.69900	-3.19800	3.31400
H	-2.19700	-4.82200	1.49000
N	-3.40800	0.12900	-1.35200
C	-2.75100	-0.87600	-2.23900
H	-3.22300	-1.84900	-2.07500
H	-2.88500	-0.56300	-3.27800
H	-1.68700	-0.91200	-1.98700
C	-2.75500	1.45600	-1.55000

H	-2.88700	1.75900	-2.59200
H	-3.22900	2.18200	-0.88300
H	-1.69100	1.35300	-1.31600
C	-3.24800	-0.29100	0.07000
H	-3.73300	0.45000	0.71100
H	-3.71500	-1.27100	0.20200
H	-2.17900	-0.34700	0.29400
C	-4.85600	0.22600	-1.68500
H	-4.95600	0.53500	-2.72900
H	-5.31800	-0.75400	-1.53600
H	-5.31900	0.96600	-1.02700

18aB: ΔG = -3669681.031 kJ/mol

Si	-1.00000	0.01100	0.49900
C	0.30900	-1.07500	1.35100
C	0.88300	-0.71600	2.58100
C	0.77200	-2.25000	0.73700
C	1.89800	-1.48100	3.16000
H	0.54400	0.18800	3.09500
C	1.76200	-3.03900	1.32500
H	0.35400	-2.55500	-0.22700
C	2.33800	-2.65000	2.53500
H	2.34500	-1.16800	4.10600
H	2.09600	-3.95400	0.83100
H	3.12500	-3.25400	2.99000
F	-1.98900	0.02600	1.98600
F	0.02800	0.00200	-0.99200
C	-2.41100	-0.83000	-0.46400
C	-3.10300	-1.92400	0.08300
C	-2.79700	-0.39100	-1.74200
C	-4.12500	-2.56300	-0.62000
H	-2.83500	-2.29000	1.07800
C	-3.83900	-1.00600	-2.43800
H	-2.27400	0.45100	-2.20400
C	-4.50300	-2.10000	-1.88200
H	-4.63600	-3.42200	-0.17900
H	-4.13100	-0.63500	-3.42300
H	-5.31100	-2.59100	-2.42900
C	-0.88000	1.91200	0.54100
C	0.35700	2.56800	0.43500
C	-2.02900	2.71100	0.66800
C	0.44900	3.96000	0.47100
H	1.27000	1.97900	0.32700
C	-1.94800	4.10400	0.67400
H	-3.01000	2.23600	0.76300
C	-0.70700	4.73500	0.58200
H	1.42600	4.44500	0.40300
H	-2.85900	4.70200	0.75700

H	-0.64000	5.82500	0.59700
N	3.53000	-0.00300	-1.63800
C	2.78700	1.13800	-2.25200
H	3.22400	2.07300	-1.89200
H	2.88500	1.07000	-3.33800
H	1.73700	1.06000	-1.95600
C	2.92500	-1.28800	-2.09800
H	3.01600	-1.34500	-3.18500
H	3.46500	-2.11500	-1.63000
H	1.87200	-1.29500	-1.80200
C	3.42800	0.08200	-0.15200
H	3.99100	-0.74800	0.28400
H	3.85000	1.03600	0.17500
H	2.37200	0.01200	0.12200
C	4.96100	0.05500	-2.04700
H	5.01700	-0.00600	-3.13600
H	5.38800	1.00000	-1.69900
H	5.49100	-0.78700	-1.59400

18bA: ΔG = -3970168.19 kJ/mol

Si	1.25000	-0.26900	0.32400
C	1.22300	1.60700	0.67100
C	0.42900	2.13200	1.70400
C	1.94800	2.51800	-0.11500
C	0.33600	3.50700	1.92500
H	-0.14400	1.45300	2.34300
C	1.88500	3.89200	0.12000
H	2.57000	2.14800	-0.93500
C	1.06900	4.39400	1.13500
H	-0.30800	3.89000	2.72000
H	2.46700	4.57900	-0.50000
H	1.00300	5.47000	1.30900
F	1.68400	-0.57300	2.03100
F	0.85200	0.07200	-1.40000
C	2.86300	-1.11400	-0.24500
C	4.09500	-0.82500	0.36700
C	2.86600	-2.05300	-1.29100
C	5.27800	-1.43400	-0.05300
H	4.13400	-0.10500	1.18900
C	4.04000	-2.68900	-1.69700
H	1.93000	-2.29500	-1.80100
C	5.25400	-2.37600	-1.08300
H	6.22500	-1.17900	0.43000
H	4.01000	-3.42700	-2.50200
H	6.17600	-2.86300	-1.40700
C	-0.37900	-1.23800	0.52900
C	-1.05900	-1.80700	-0.55900
C	-1.00900	-1.36100	1.78500

C	-2.29000	-2.45500	-0.42700
H	-0.62500	-1.73900	-1.55800
C	-2.24400	-1.97700	1.94000
H	-0.51900	-0.95300	2.67000
C	-2.89900	-2.53200	0.83100
H	-2.76800	-2.88800	-1.30500
H	-2.72100	-2.04400	2.92000
N	-2.64700	2.08300	-1.26200
C	-2.25700	1.05200	-2.26900
H	-3.01600	0.26500	-2.27100
H	-2.20700	1.52900	-3.25100
H	-1.27900	0.64700	-1.99100
C	-1.61400	3.16100	-1.23500
H	-1.54900	3.60400	-2.23200
H	-1.91600	3.91600	-0.50500
H	-0.65400	2.71700	-0.95500
C	-2.73700	1.44700	0.08400
H	-3.00000	2.21300	0.81900
H	-3.50600	0.67100	0.05300
H	-1.76500	1.00800	0.32400
C	-3.96800	2.66800	-1.62700
H	-3.88500	3.13500	-2.61200
H	-4.71100	1.86700	-1.65100
H	-4.24100	3.41400	-0.87700
O	-4.10000	-3.11700	1.06200
C	-4.78700	-3.67300	-0.04500
H	-5.72500	-4.08000	0.34200
H	-5.01200	-2.91000	-0.80500
H	-4.21000	-4.48400	-0.51200

18bB: ΔG = -3970163.31 kJ/mol

Si	-0.32500	0.46900	0.75200
C	0.87800	-0.75200	1.57800
C	1.80400	-0.33100	2.54500
C	0.90100	-2.10700	1.20500
C	2.73400	-1.21400	3.09600
H	1.81100	0.71400	2.86800
C	1.80500	-3.00400	1.77600
H	0.20000	-2.47000	0.44800
C	2.73400	-2.55700	2.71600
H	3.46000	-0.85500	3.82900
H	1.79400	-4.05500	1.47600
H	3.45500	-3.25200	3.15100
F	-0.89200	1.01400	2.35800
F	0.30300	-0.07000	-0.86100
C	-2.07200	-0.11100	0.28900
C	-2.89300	-0.77500	1.22000
C	-2.61000	0.10000	-0.98800

C	-4.16900	-1.21700	0.89200
H	-2.52400	-0.95800	2.23200
C	-3.90200	-0.30800	-1.33300
H	-2.01000	0.60300	-1.75000
C	-4.68800	-0.98000	-0.38900
H	-4.78800	-1.74400	1.62100
H	-4.28300	-0.10700	-2.33400
C	0.22800	2.24700	0.34000
C	1.51500	2.52200	-0.15100
C	-0.64500	3.33400	0.51000
C	1.92100	3.82400	-0.44500
H	2.21900	1.70100	-0.30400
C	-0.25800	4.63600	0.18900
H	-1.65100	3.16000	0.90100
C	1.03100	4.88700	-0.28400
H	2.93400	4.01200	-0.81000
H	-0.96300	5.46200	0.31600
H	1.34000	5.90600	-0.52600
N	3.48100	-1.17000	-2.06100
C	2.94700	0.02000	-2.78700
H	3.68200	0.82600	-2.72300
H	2.78100	-0.25700	-3.83200
H	2.00500	0.31400	-2.31600
C	2.47500	-2.27200	-2.11400
H	2.30200	-2.53500	-3.16000
H	2.87000	-3.13300	-1.56900
H	1.55100	-1.91200	-1.65200
C	3.73400	-0.80600	-0.63600
H	4.14400	-1.67800	-0.12000
H	4.44900	0.02000	-0.60700
H	2.78200	-0.50900	-0.18700
C	4.75200	-1.61900	-2.69500
H	4.55100	-1.88400	-3.73600
H	5.47500	-0.80000	-2.64700
H	5.12900	-2.48800	-2.14900
O	-5.94600	-1.42900	-0.62700
C	-6.49900	-1.19800	-1.90900
H	-5.91000	-1.68700	-2.69900
H	-7.50500	-1.62700	-1.89900
H	-6.57200	-0.12200	-2.13100

18cA: ΔG = -4554222.10 kJ/mol

Si	-1.51000	-0.48200	-0.31000
C	-1.94200	1.31600	-0.77100
C	-1.26000	1.98200	-1.80300
C	-2.91500	2.04100	-0.06400
C	-1.51600	3.32200	-2.09900
H	-0.50000	1.44900	-2.38100

C	-3.20000	3.37100	-0.37500
H	-3.45900	1.56000	0.75400
C	-2.49300	4.02100	-1.38800
H	-0.95500	3.82200	-2.89100
H	-3.97100	3.90900	0.18300
H	-2.70100	5.06700	-1.62200
F	-1.71300	-0.97400	-2.01000
F	-1.32100	0.03000	1.40200
C	-2.86600	-1.71000	0.22700
C	-4.06300	-1.83800	-0.49800
C	-2.70500	-2.52600	1.35900
C	-5.06200	-2.72800	-0.10200
H	-4.22200	-1.22600	-1.39000
C	-3.68500	-3.44100	1.74400
H	-1.79200	-2.44600	1.95500
C	-4.87300	-3.54000	1.01800
H	-5.99100	-2.79500	-0.67300
H	-3.52700	-4.07500	2.61900
H	-5.64800	-4.24600	1.32300
C	0.34000	-0.99000	-0.35900
C	1.05000	-1.32200	0.81000
C	1.06600	-0.97100	-1.56200
C	2.41200	-1.60500	0.78500
H	0.52900	-1.34700	1.76700
C	2.43600	-1.22600	-1.60100
H	0.55100	-0.74000	-2.49500
C	3.11200	-1.54500	-0.42400
H	2.93800	-1.85900	1.70700
H	2.97600	-1.18000	-2.54700
N	1.53600	2.89700	1.33500
C	1.33500	1.84300	2.37300
H	2.26200	1.27200	2.47400
H	1.09000	2.33100	3.32000
H	0.51300	1.19400	2.05400
C	0.27300	3.67500	1.17400
H	0.02300	4.13500	2.13400
H	0.43100	4.44700	0.41600
H	-0.52200	2.99100	0.86500
C	1.88800	2.25000	0.03800
H	2.01800	3.02900	-0.71900
H	2.81600	1.68700	0.16700
H	1.07100	1.58100	-0.24600
C	2.63800	3.81000	1.75100
H	2.36300	4.28400	2.69700
H	3.55200	3.22400	1.87400
H	2.77700	4.56800	0.97600
C	4.58900	-1.78100	-0.41700
F	4.92500	-2.92000	0.23000

F	5.25700	-0.78600	0.22300
F	5.11900	-1.86200	-1.64800

18cB: ΔG = -4554216.86 kJ/mol

Si	-0.77600	1.05400	0.45000
C	-1.96800	-0.12000	1.35200
C	-1.60700	-0.75500	2.55000
C	-3.22900	-0.42400	0.80900
C	-2.45400	-1.67800	3.16800
H	-0.63700	-0.53800	3.00700
C	-4.09500	-1.31800	1.43800
H	-3.53900	0.04500	-0.12900
C	-3.70400	-1.95800	2.61500
H	-2.13900	-2.17800	4.08700
H	-5.07400	-1.52800	1.00100
H	-4.37200	-2.67400	3.10000
F	-0.50000	1.97200	1.95600
F	-1.02000	0.09500	-1.06000
C	-1.44900	2.60900	-0.41500
C	-2.38800	3.44100	0.21700
C	-1.03000	2.96900	-1.70700
C	-2.89900	4.57500	-0.41600
H	-2.73400	3.19700	1.22500
C	-1.51200	4.11900	-2.33500
H	-0.30800	2.34000	-2.23400
C	-2.45500	4.92300	-1.69300
H	-3.64100	5.19600	0.09100
H	-1.15600	4.38600	-3.33300
H	-2.84300	5.81700	-2.18600
C	1.08700	0.65400	0.36300
C	1.54000	-0.66400	0.21000
C	2.05600	1.66900	0.44700
C	2.89800	-0.97200	0.15300
H	0.81600	-1.47700	0.13600
C	3.41500	1.38300	0.36300
H	1.74500	2.70700	0.58000
C	3.83900	0.05700	0.22100
H	3.22500	-2.00700	0.04900
H	4.15300	2.18600	0.41700
N	-1.49100	-3.49600	-1.60100
C	-0.30300	-2.89100	-2.27400
H	0.59300	-3.42900	-1.95200
H	-0.43200	-2.98600	-3.35500
H	-0.24800	-1.83700	-1.98700
C	-2.71700	-2.75400	-2.01600
H	-2.83500	-2.85500	-3.09800
H	-3.58000	-3.18500	-1.50100
H	-2.59100	-1.70200	-1.74300

C	-1.32900	-3.39200	-0.12200
H	-2.19400	-3.85700	0.35800
H	-0.41200	-3.91300	0.16800
H	-1.27200	-2.33300	0.14300
C	-1.61200	-4.92900	-1.98800
H	-1.72500	-4.99200	-3.07400
H	-0.70600	-5.45400	-1.67300
H	-2.48700	-5.35500	-1.49200
C	5.30500	-0.22800	0.15600
F	5.58800	-1.53000	-0.01800
F	5.95200	0.16100	1.28200
F	5.91000	0.43900	-0.85700

TS 18aA - 19a: ΔG = -3669683.40 kJ/mol

Si	-1.32000	0.05200	1.06800
C	-0.23200	-1.46700	1.19400
C	0.48000	-1.69600	2.38500
C	-0.03900	-2.35500	0.12300
C	1.36000	-2.77100	2.49900
H	0.35400	-1.02400	3.23700
C	0.83800	-3.43300	0.23700
H	-0.55200	-2.17500	-0.82300
C	1.54100	-3.64200	1.42400
H	1.90800	-2.93100	3.43000
H	0.97700	-4.11100	-0.60800
H	2.23200	-4.48300	1.51000
F	-2.04800	0.12300	2.56500
F	0.07300	-0.21200	-2.20100
C	-2.67500	-0.08900	-0.20800
C	-3.26500	-1.33300	-0.49400
C	-3.15600	1.04200	-0.88900
C	-4.29300	-1.44300	-1.42900
H	-2.92200	-2.23400	0.02000
C	-4.18400	0.93500	-1.82400
H	-2.72600	2.02600	-0.68800
C	-4.75100	-0.30900	-2.10000
H	-4.73700	-2.41900	-1.63700
H	-4.54200	1.82600	-2.34300
H	-5.55400	-0.39500	-2.83500
C	-0.36100	1.65900	0.98300
C	0.02300	2.23100	-0.24300
C	0.03000	2.30200	2.17100
C	0.77100	3.40700	-0.27700
H	-0.22600	1.71000	-1.17200
C	0.78700	3.47300	2.13500
H	-0.25700	1.88600	3.13900
C	1.15600	4.03000	0.91100
H	1.06000	3.83800	-1.23800

H	1.08500	3.95600	3.06800
H	1.74500	4.94900	0.88300
N	3.36600	-0.08300	-1.43500
C	2.98600	1.09900	-2.26400
H	3.22900	2.00700	-1.70500
H	3.55800	1.06900	-3.19500
H	1.91000	1.02600	-2.46100
C	3.03400	-1.33100	-2.18400
H	3.60300	-1.33700	-3.11800
H	3.31500	-2.19000	-1.56900
H	1.95500	-1.31500	-2.38000
C	2.56900	-0.05800	-0.17300
H	2.83200	-0.93800	0.42200
H	2.81100	0.85600	0.37700
H	1.51500	-0.07800	-0.46900
C	4.82000	-0.04500	-1.12400
H	5.38300	-0.06400	-2.06000
H	5.03900	0.87300	-0.57200
H	5.07200	-0.91800	-0.51500

TS 18bA - 19b: ΔG = -3970095.32 kJ/mol

Si	1.42300	-0.55800	1.06000
C	1.53200	1.31300	1.11200
C	1.44600	1.98700	2.34300
C	1.65000	2.08100	-0.06000
C	1.46900	3.38000	2.40200
H	1.35800	1.41900	3.27300
C	1.68400	3.47300	0.00000
H	1.66300	1.57100	-1.02700
C	1.59100	4.12700	1.22900
H	1.39600	3.88500	3.36700
H	1.77700	4.05400	-0.92100
H	1.61200	5.21800	1.27500
F	1.95900	-1.00100	2.57800
F	0.36800	0.21500	-2.17700
C	2.58200	-1.33400	-0.18400
C	3.68800	-0.62800	-0.68700
C	2.38300	-2.65400	-0.62700
C	4.56000	-1.21600	-1.60300
H	3.87800	0.39700	-0.36200
C	3.25200	-3.24400	-1.54400
H	1.53600	-3.23400	-0.25400
C	4.34100	-2.52400	-2.03600
H	5.41300	-0.65000	-1.98300
H	3.07800	-4.26900	-1.87600
H	5.02100	-2.98300	-2.75700
C	-0.32100	-1.21700	1.01100
C	-0.92100	-1.72000	-0.15300

C	-1.12300	-1.12700	2.16700
C	-2.25800	-2.11600	-0.17800
H	-0.34700	-1.75300	-1.08000
C	-2.45600	-1.51100	2.15900
H	-0.70100	-0.74100	3.09900
C	-3.03700	-2.00700	0.98100
H	-2.68600	-2.49700	-1.10500
H	-3.07100	-1.43300	3.05700
N	-2.36200	2.10400	-1.52100
C	-2.73500	0.92200	-2.35300
H	-3.48100	0.33600	-1.81000
H	-3.15600	1.28000	-3.29600
H	-1.81900	0.34300	-2.52500
C	-1.34200	2.91200	-2.25300
H	-1.78000	3.25200	-3.19600
H	-1.07100	3.77100	-1.63400
H	-0.47800	2.26100	-2.43300
C	-1.76200	1.62100	-0.24200
H	-1.49500	2.48900	0.36800
H	-2.50300	1.00600	0.27900
H	-0.87800	1.03300	-0.51200
C	-3.56600	2.93300	-1.24400
H	-3.98900	3.26900	-2.19400
H	-4.29500	2.32500	-0.70200
H	-3.26900	3.79300	-0.63900
O	-4.34300	-2.35100	1.05100
C	-4.96800	-2.83200	-0.12700
H	-4.49400	-3.75800	-0.48500
H	-6.00800	-3.04100	0.13500
H	-4.94300	-2.08100	-0.93000

TS 18cA - 19c: ΔG = -3970095.32 kJ/mol

Si	-0.09400	0.33100	-1.61200
C	-1.08500	-1.25700	-1.58300
C	-2.13200	-1.41500	-2.50700
C	-0.87100	-2.27400	-0.63800
C	-2.94000	-2.55100	-2.48900
H	-2.32600	-0.64100	-3.25400
C	-1.67600	-3.41300	-0.62100
H	-0.08900	-2.15700	0.11400
C	-2.71200	-3.55400	-1.54500
H	-3.75000	-2.65600	-3.21300
H	-1.49600	-4.19300	0.12200
H	-3.34400	-4.44400	-1.52800
F	0.12700	0.62700	-3.23300
F	-0.24000	-0.35400	1.93500
C	1.60800	0.15800	-0.85000
C	2.32400	-1.04400	-0.95900

C	2.22000	1.23700	-0.18900
C	3.60000	-1.17600	-0.41800
H	1.88700	-1.90200	-1.47400
C	3.49200	1.11500	0.35700
H	1.69700	2.19100	-0.09100
C	4.18200	-0.09600	0.24400
H	4.14000	-2.11800	-0.51200
H	3.95300	1.96000	0.87200
C	-1.04100	1.82900	-1.01300
C	-1.07200	2.18900	0.34600
C	-1.78300	2.59900	-1.92700
C	-1.81600	3.28800	0.77400
H	-0.54500	1.56300	1.07400
C	-2.53500	3.69200	-1.49500
H	-1.77500	2.34800	-2.99000
C	-2.55100	4.04000	-0.14400
H	-1.82800	3.55700	1.83200
H	-3.10800	4.27600	-2.21800
H	-3.13600	4.89800	0.19300
N	-3.61600	-0.52900	2.32000
C	-3.10300	0.65000	3.07800
H	-3.61700	1.54500	2.71800
H	-3.31300	0.49800	4.14000
H	-2.02300	0.71000	2.89900
C	-2.92200	-1.75900	2.80600
H	-3.14700	-1.88700	3.86800
H	-3.29700	-2.61600	2.23800
H	-1.84700	-1.61100	2.64400
C	-3.30200	-0.34300	0.87200
H	-3.67700	-1.21000	0.32100
H	-3.79200	0.57000	0.52100
H	-2.21200	-0.26600	0.79500
C	-5.08500	-0.66100	2.51400
H	-5.28900	-0.79200	3.58000
H	-5.57300	0.24600	2.14600
H	-5.43700	-1.53100	1.95300
C	5.55000	-0.20200	0.84600
F	6.10500	-1.41400	0.68500
F	5.53900	0.04900	2.17500
F	6.41000	0.69400	0.30800

19a: ΔG = -3669619.49 kJ/mol

Si	1.53600	0.00000	1.06800
C	0.50100	1.53700	0.83300
C	-0.14000	2.16400	1.91400
C	0.24700	2.02000	-0.46300
C	-1.00900	3.23500	1.70700
H	0.03400	1.80800	2.93300

C	-0.62700	3.08500	-0.67300
H	0.72600	1.54900	-1.32600
C	-1.25600	3.69400	0.41300
H	-1.49800	3.71200	2.55900
H	-0.81900	3.43800	-1.68800
H	-1.94300	4.52700	0.25000
F	2.00000	0.00000	2.65800
F	-1.45100	0.00000	-2.92700
C	3.03900	-0.00100	-0.04000
C	3.62500	1.20600	-0.46100
C	3.62300	-1.20800	-0.46300
C	4.75800	1.20700	-1.27200
H	3.19200	2.16100	-0.15200
C	4.75600	-1.20900	-1.27500
H	3.18800	-2.16200	-0.15700
C	5.32400	-0.00100	-1.68200
H	5.20000	2.15300	-1.59000
H	5.19500	-2.15500	-1.59500
H	6.20900	-0.00100	-2.32100
C	0.50000	-1.53700	0.83300
C	0.24400	-2.01900	-0.46300
C	-0.14000	-2.16400	1.91500
C	-0.63000	-3.08300	-0.67300
H	0.72200	-1.54700	-1.32600
C	-1.01000	-3.23500	1.70800
H	0.03500	-1.80900	2.93300
C	-1.25900	-3.69300	0.41400
H	-0.82400	-3.43500	-1.68800
H	-1.49800	-3.71100	2.56000
H	-1.94600	-4.52600	0.25100
N	-3.86200	0.00000	-0.59400
C	-3.98100	-1.21500	-1.45200
H	-3.88200	-2.10000	-0.81700
H	-4.96300	-1.20600	-1.93300
H	-3.17700	-1.16600	-2.19600
C	-3.98200	1.21500	-1.45200
H	-4.96400	1.20500	-1.93300
H	-3.88400	2.10000	-0.81800
H	-3.17800	1.16700	-2.19600
C	-2.51600	0.00100	0.05500
H	-2.43200	0.90000	0.67400
H	-2.43100	-0.89600	0.67600
H	-1.77500	0.00000	-0.75200
C	-4.92800	0.00000	0.44300
H	-5.90300	0.00000	-0.05200
H	-4.81900	-0.89600	1.06000
H	-4.82000	0.89600	1.06000

19b: ΔG = -3970106.09 kJ/mol

Si	-1.77800	-0.01800	1.05900
C	-0.36300	-1.21700	0.91500
C	0.43300	-1.58600	2.01500
C	0.03800	-1.66600	-0.35300
C	1.57800	-2.35500	1.85400
H	0.16300	-1.25800	3.02200
H	-0.54600	-1.40100	-1.23900
C	1.96900	-2.77800	0.57400
H	2.19200	-2.63600	2.71100
F	-2.21600	0.01000	2.65500
F	1.40600	0.45000	-2.86500
C	-3.23300	-0.53400	0.00400
C	-3.46500	-1.89000	-0.28700
C	-4.13000	0.41900	-0.50900
C	-4.56000	-2.28100	-1.05600
H	-2.78000	-2.65300	0.09200
C	-5.22500	0.03000	-1.27900
H	-3.97300	1.48200	-0.30800
C	-5.44100	-1.32100	-1.55400
H	-4.72400	-3.33900	-1.27100
H	-5.91200	0.78400	-1.67000
H	-6.29600	-1.62600	-2.15900
C	-1.22200	1.71800	0.64100
C	-1.04500	2.08100	-0.70500
C	-0.85800	2.64300	1.63300
C	-0.51100	3.32100	-1.05100
H	-1.31300	1.38000	-1.50100
C	-0.33100	3.88900	1.29000
H	-0.97900	2.38800	2.68800
C	-0.15300	4.22800	-0.05200
H	-0.37200	3.57900	-2.10200
H	-0.05400	4.59700	2.07300
H	0.26600	5.19900	-0.32000
N	3.50000	1.27900	-0.38200
C	3.44000	2.35200	-1.41700
H	3.09900	3.27500	-0.94000
H	4.44200	2.49200	-1.83100
H	2.73400	2.01900	-2.18800
C	3.96500	0.01100	-1.01600
H	4.96100	0.17500	-1.43500
H	4.00500	-0.76400	-0.24500
H	3.24400	-0.24200	-1.80300
C	2.12700	1.06300	0.16700
H	2.17700	0.27000	0.91900
H	1.78600	1.99700	0.62100
H	1.49100	0.77300	-0.67700
C	4.42900	1.67100	0.71000

H	5.42600	1.82400	0.28900
H	4.06700	2.59700	1.16600
H	4.45400	0.87100	1.45500
O	3.10500	-3.50600	0.49900
C	1.18600	-2.43300	-0.53600
C	3.52200	-3.95800	-0.77900
H	4.45100	-4.51200	-0.62400
H	2.77500	-4.62800	-1.23000
H	3.71200	-3.12000	-1.46500
H	1.46600	-2.75000	-1.54000

19c: ΔG = -3970106.09 kJ/mol

Si	0.24100	0.01200	1.57000
C	-0.70400	1.54800	1.09100
C	-1.55800	2.20300	1.99500
C	-0.66500	2.00200	-0.23900
C	-2.34800	3.27500	1.58100
H	-1.61400	1.86800	3.03400
C	-1.46000	3.06700	-0.65600
H	-0.01700	1.50700	-0.96700
C	-2.30300	3.70600	0.25500
H	-3.00500	3.77400	2.29600
H	-1.42400	3.39800	-1.69600
H	-2.92700	4.54000	-0.07100
F	0.35400	0.02000	3.21900
F	-1.83900	-0.00600	-2.99700
C	1.95700	0.00500	0.81900
C	2.62000	1.20800	0.52600
C	2.61700	-1.20300	0.54500
C	3.90000	1.20800	-0.02000
H	2.13400	2.16600	0.72200
C	3.89700	-1.21500	0.00000
H	2.12800	-2.15600	0.75700
C	4.53800	-0.00700	-0.28300
H	4.40100	2.14900	-0.24500
H	4.39600	-2.16200	-0.20900
C	-0.70700	-1.52700	1.10600
C	-0.67200	-1.98800	-0.22200
C	-1.55900	-2.17400	2.01600
C	-1.47100	-3.05400	-0.63000
H	-0.02500	-1.49900	-0.95500
C	-2.35300	-3.24700	1.61100
H	-1.61200	-1.83300	3.05200
C	-2.31200	-3.68600	0.28700
H	-1.43800	-3.39100	-1.66800
H	-3.00900	-3.74000	2.33000
H	-2.94000	-4.52100	-0.03200
N	-4.67000	-0.01600	-1.21000

C	-4.58400	-1.24700	-2.05000
H	-4.62100	-2.11900	-1.39100
H	-5.43500	-1.25900	-2.73700
H	-3.63500	-1.20000	-2.59700
C	-4.61400	1.18400	-2.09600
H	-5.46600	1.15000	-2.78100
H	-4.67000	2.07900	-1.47100
H	-3.66500	1.13800	-2.64300
C	-3.49800	0.01600	-0.28400
H	-3.56000	0.92600	0.32000
H	-3.53800	-0.86900	0.35700
H	-2.59900	0.01400	-0.91100
C	-5.93500	-0.01800	-0.43000
H	-6.77900	-0.04100	-1.12500
H	-5.95200	-0.90200	0.21300
H	-5.97500	0.88900	0.17900
C	5.89200	-0.01200	-0.92600
F	5.80700	0.00300	-2.27900
F	6.63000	1.06000	-0.58400
F	6.61200	-1.10300	-0.60700

Computations using ORCA, method CPCM(MeCN)-M062X/ma-def2-TZVP

18aA: ΔG = -3671016.13 kJ/mol

Si	1.05998	-0.07188	0.22719
C	0.18928	1.62690	0.32366
C	-0.57234	1.98806	1.44187
C	0.27111	2.55910	-0.71726
C	-1.24015	3.20632	1.51134
H	-0.65070	1.29593	2.27341
C	-0.36247	3.79580	-0.64501
H	0.84039	2.31059	-1.60571
C	-1.12960	4.11909	0.46796
H	-1.83919	3.44798	2.38117
H	-0.26788	4.50241	-1.46106
H	-1.63787	5.07408	0.52184
F	1.43863	0.04488	1.91317
F	0.68409	-0.14976	-1.46952
C	2.91644	-0.19860	-0.19626
C	3.89130	0.45028	0.57021
C	3.36546	-0.94218	-1.29464
C	5.24374	0.37743	0.25144
H	3.58606	1.02656	1.43557
C	4.71677	-1.04740	-1.60649
H	2.64164	-1.45032	-1.92094
C	5.66185	-0.37907	-0.83671
H	5.97214	0.90524	0.85571

H	5.03250	-1.64322	-2.45469
H	6.71446	-0.44727	-1.08309
C	0.01201	-1.64346	0.54221
C	-0.35814	-2.50778	-0.49643
C	-0.47865	-1.95063	1.81880
C	-1.17174	-3.61550	-0.27906
H	-0.01134	-2.30388	-1.50158
C	-1.30974	-3.04269	2.04547
H	-0.21378	-1.31314	2.65264
C	-1.65881	-3.88264	0.99472
H	-1.43279	-4.26505	-1.10628
H	-1.68321	-3.23990	3.04336
H	-2.30296	-4.73628	1.16670
N	-3.54805	0.18226	-1.02981
C	-2.63940	-0.41179	-2.05270
H	-2.78525	-1.48972	-2.05234
H	-2.89715	0.00961	-3.02209
H	-1.61058	-0.17040	-1.78492
C	-3.37613	1.66397	-1.02757
H	-3.69973	2.04657	-1.99305
H	-3.98215	2.08157	-0.22710
H	-2.32178	1.87945	-0.86643
C	-3.19345	-0.35509	0.31549
H	-3.88635	0.05973	1.04395
H	-3.26212	-1.44151	0.28625
H	-2.17376	-0.05393	0.53711
C	-4.96118	-0.16153	-1.34885
H	-5.19214	0.21910	-2.34084
H	-5.06494	-1.24372	-1.32123
H	-5.60312	0.30212	-0.60345

18aB: ΔG = -3671011.44 kJ/mol

Si	-0.98927	0.03351	0.50374
C	0.36232	-0.99965	1.34533
C	1.00270	-0.54883	2.50324
C	0.81386	-2.20018	0.78820
C	2.06521	-1.24892	3.06781
H	0.67734	0.37653	2.96912
C	1.85608	-2.92323	1.35803
H	0.34831	-2.57720	-0.11785
C	2.49298	-2.44191	2.49687
H	2.55639	-0.86634	3.95465
H	2.17955	-3.85521	0.90946
H	3.31561	-2.99275	2.93608
F	-1.98109	-0.07153	1.92616
F	0.04952	0.14557	-0.91298
C	-2.33459	-0.81901	-0.54000
C	-3.22946	-1.73324	0.02929

C	-2.47545	-0.56353	-1.90948
C	-4.20726	-2.37085	-0.72706
H	-3.15977	-1.95216	1.08783
C	-3.46641	-1.17395	-2.67091
H	-1.79570	0.12894	-2.39083
C	-4.33323	-2.08680	-2.08159
H	-4.87518	-3.08364	-0.25820
H	-3.55860	-0.94283	-3.72547
H	-5.09980	-2.57256	-2.67294
C	-0.94106	1.92673	0.66546
C	0.25629	2.64040	0.54733
C	-2.10289	2.66579	0.91090
C	0.29950	4.02459	0.67650
H	1.18033	2.10170	0.36386
C	-2.07866	4.05322	1.01126
H	-3.04898	2.14700	1.02461
C	-0.87341	4.73697	0.89926
H	1.24485	4.54803	0.59664
H	-2.99756	4.60079	1.18391
H	-0.84788	5.81623	0.98706
N	3.45038	-0.11940	-1.68470
C	2.80871	1.05560	-2.34304
H	3.37090	1.94645	-2.07208
H	2.83660	0.90042	-3.41903
H	1.78328	1.11979	-1.98508
C	2.70948	-1.35782	-2.06131
H	2.78815	-1.48780	-3.13814
H	3.16297	-2.19836	-1.54033
H	1.67298	-1.23104	-1.75727
C	3.39238	0.05756	-0.20489
H	3.88081	-0.79351	0.26487
H	3.90449	0.98386	0.04654
H	2.34562	0.09649	0.08693
C	4.86906	-0.22948	-2.12223
H	4.88729	-0.34339	-3.20347
H	5.39064	0.67742	-1.82552
H	5.30964	-1.09850	-1.63955

18bA: ΔG = -3971635.81 kJ/mol

Si	-1.26013	-0.25948	-0.24964
C	-1.23455	1.63900	-0.47259
C	-0.65641	2.23184	-1.60132
C	-1.77092	2.49992	0.49155
C	-0.58868	3.61303	-1.75138
H	-0.23973	1.59700	-2.37636
C	-1.74323	3.88270	0.33645
H	-2.21554	2.08011	1.38682
C	-1.14019	4.44454	-0.78303

H	-0.11367	4.04146	-2.62602
H	-2.18003	4.52213	1.09441
H	-1.10056	5.52059	-0.90016
F	-1.58569	-0.44096	-1.94247
F	-0.95309	-0.03835	1.44970
C	-2.88662	-1.16441	0.17733
C	-4.00820	-1.10007	-0.65753
C	-3.01190	-1.92364	1.34740
C	-5.19961	-1.74370	-0.33722
H	-3.95030	-0.53179	-1.57819
C	-4.18784	-2.59514	1.66499
H	-2.17043	-1.98989	2.02686
C	-5.29105	-2.50040	0.82476
H	-6.05451	-1.66008	-0.99781
H	-4.24661	-3.18563	2.57168
H	-6.21326	-3.01123	1.07369
C	0.37956	-1.21701	-0.43673
C	1.06369	-1.74660	0.65917
C	1.00876	-1.36750	-1.68369
C	2.29677	-2.38740	0.54671
H	0.63277	-1.65001	1.64797
C	2.24273	-1.97859	-1.82340
H	0.52204	-0.98215	-2.57047
C	2.89654	-2.49538	-0.70398
H	2.77582	-2.78290	1.43156
H	2.71864	-2.06955	-2.79243
N	2.69252	2.06759	1.07139
C	2.12803	1.18561	2.13325
H	2.72304	0.27582	2.17171
H	2.18263	1.71913	3.07971
H	1.09389	0.95065	1.88200
C	1.88256	3.31744	0.99100
H	1.99072	3.85407	1.93091
H	2.25337	3.91684	0.16280
H	0.84211	3.03868	0.83367
C	2.63894	1.35986	-0.23975
H	3.09756	1.99781	-0.99212
H	3.17734	0.41790	-0.14783
H	1.59712	1.16989	-0.47987
C	4.10650	2.40327	1.39772
H	4.12761	2.90177	2.36373
H	4.67809	1.47873	1.43253
H	4.48984	3.05985	0.62013
O	4.10154	-3.08130	-0.92685
C	4.81100	-3.57371	0.19718
H	5.74481	-3.97636	-0.18507
H	5.02033	-2.77065	0.90768
H	4.24923	-4.36412	0.69969

18bB: ΔG = -3971627.60 kJ/mol

Si	1.20756	-0.08545	-0.51782
C	-0.48116	-0.81375	-0.97495
C	-1.15576	-0.44017	-2.14440
C	-1.14762	-1.69395	-0.12559
C	-2.43412	-0.89108	-2.43126
H	-0.67867	0.23726	-2.84615
C	-2.42253	-2.18682	-0.40114
H	-0.67096	-2.01070	0.79750
C	-3.07513	-1.76998	-1.55737
H	-2.95325	-0.57800	-3.32916
H	-2.89286	-2.87305	0.28929
F	1.90002	-0.73656	-1.97132
F	0.47907	0.58518	0.93770
C	2.39871	-1.11631	0.55375
C	2.88170	-2.35526	0.11568
C	2.82705	-0.67771	1.81243
C	3.73447	-3.12768	0.89761
H	2.58177	-2.72545	-0.85727
C	3.70110	-1.42815	2.59188
H	2.46743	0.27137	2.19146
C	4.15346	-2.66164	2.13803
H	4.07814	-4.08959	0.53609
H	4.02472	-1.05414	3.55599
H	4.82584	-3.25444	2.74608
C	1.67589	1.67736	-1.07351
C	0.86936	2.78237	-0.77880
C	2.84943	1.92408	-1.79573
C	1.20417	4.06759	-1.19262
H	-0.04283	2.63623	-0.21229
C	3.21192	3.20824	-2.18863
H	3.49488	1.09294	-2.05470
C	2.38422	4.28542	-1.89364
H	0.54932	4.89940	-0.96160
H	4.13613	3.36817	-2.73114
H	2.65664	5.28578	-2.20719
N	-2.73642	1.42213	2.05353
C	-1.75231	2.48000	2.42269
H	-2.08261	3.42082	1.98804
H	-1.71886	2.55283	3.50726
H	-0.78294	2.18650	2.02519
C	-2.27872	0.11594	2.60957
H	-2.23451	0.20476	3.69275
H	-2.99464	-0.64854	2.31529
H	-1.29679	-0.09590	2.19324
C	-2.81936	1.32538	0.56697
H	-3.52431	0.53604	0.31279

H	-3.16016	2.28430	0.18279
H	-1.83073	1.08034	0.18645
C	-4.07598	1.76344	2.60697
H	-3.99449	1.82697	3.68950
H	-4.38619	2.71904	2.19099
H	-4.77359	0.97945	2.32220
O	-4.32912	-2.16233	-1.91153
C	-5.01523	-3.03584	-1.03177
H	-5.98716	-3.21920	-1.48137
H	-4.47880	-3.98080	-0.92039
H	-5.14628	-2.57628	-0.04909

18cA: ΔG = -3971627.60 kJ/mol

Si	0.03370	0.02660	-0.38740
C	0.97482	1.69302	-0.32707
C	1.77019	2.11944	-1.39941
C	0.90515	2.54298	0.78413
C	2.46601	3.32370	-1.36463
H	1.84729	1.49476	-2.28169
C	1.57202	3.76387	0.81873
H	0.31323	2.24536	1.64108
C	2.36050	4.15650	-0.25642
H	3.08430	3.61605	-2.20500
H	1.48262	4.40498	1.68761
H	2.89083	5.10051	-0.22948
F	-0.10945	0.17259	-2.10077
F	0.12637	-0.08387	1.33947
C	-1.87432	-0.05786	-0.25187
C	-2.70766	0.62250	-1.14308
C	-2.49269	-0.82367	0.74540
C	-4.09372	0.56286	-1.04532
H	-2.26826	1.21503	-1.93573
C	-3.87183	-0.92192	0.84044
H	-1.88124	-1.35765	1.46185
C	-4.66956	-0.21797	-0.05498
H	-4.71641	1.11280	-1.73872
H	-4.32840	-1.53438	1.60928
C	1.04562	-1.58589	-0.57411
C	1.23706	-2.46828	0.49664
C	1.67863	-1.91564	-1.78042
C	2.01339	-3.61682	0.37536
H	0.77576	-2.24807	1.45101
C	2.47490	-3.04844	-1.90834
H	1.55593	-1.26419	-2.63623
C	2.64338	-3.90737	-0.82840
H	2.13326	-4.28025	1.22375
H	2.96186	-3.26334	-2.85217
H	3.25893	-4.79346	-0.92438

N	4.45541	0.08583	1.34396
C	3.27900	-0.22559	2.20627
H	3.22391	-1.30473	2.33049
H	3.42268	0.26423	3.16690
H	2.37793	0.13980	1.71635
C	4.57497	1.56392	1.19094
H	4.73085	1.99951	2.17521
H	5.42084	1.77467	0.54038
H	3.65112	1.93437	0.75051
C	4.26076	-0.53149	0.00040
H	5.15754	-0.35848	-0.59011
H	4.07806	-1.59704	0.13006
H	3.39920	-0.06012	-0.46514
C	5.69146	-0.45960	1.97071
H	5.80300	-0.01156	2.95528
H	5.58427	-1.53864	2.05220
H	6.53834	-0.20673	1.33736
C	-6.15611	-0.34184	0.06575
F	-6.81683	0.47603	-0.76149
F	-6.58764	-0.06790	1.30990
F	-6.58197	-1.59051	-0.20521

18cB: ΔG = -4556056.28 kJ/mol

Si	0.26554	0.65083	0.80695
C	1.39009	-0.64070	1.62403
C	2.44981	-0.25154	2.44877
C	1.24814	-2.00670	1.36212
C	3.34720	-1.17973	2.96841
H	2.59002	0.80001	2.68110
C	2.12349	-2.94644	1.89610
H	0.44322	-2.34820	0.71752
C	3.18424	-2.53273	2.69444
H	4.17105	-0.84815	3.58926
H	1.98575	-3.99973	1.68147
H	3.87754	-3.25906	3.10036
F	-0.33296	1.13785	2.35295
F	0.87150	0.11824	-0.74937
C	-1.50370	0.18218	0.25725
C	-2.44997	-0.29553	1.17322
C	-1.91053	0.30965	-1.07364
C	-3.73373	-0.64405	0.78168
H	-2.17634	-0.39676	2.21585
C	-3.20174	-0.00688	-1.48116
H	-1.20492	0.66571	-1.81370
C	-4.10729	-0.49037	-0.54918
H	-4.44508	-1.02581	1.50488
H	-3.49536	0.11600	-2.51532
C	0.93770	2.40222	0.47744

C	2.07422	2.61947	-0.31054
C	0.31634	3.53395	1.01867
C	2.57510	3.89651	-0.54013
H	2.58224	1.77162	-0.75463
C	0.79079	4.81836	0.77351
H	-0.55858	3.40858	1.64524
C	1.92828	5.00317	-0.00324
H	3.46557	4.02865	-1.14318
H	0.27739	5.67470	1.19425
H	2.30760	6.00069	-0.18843
N	3.54952	-1.61455	-2.09648
C	3.15325	-0.47968	-2.97968
H	4.01703	0.16756	-3.11368
H	2.83170	-0.88796	-3.93507
H	2.34058	0.05503	-2.49282
C	2.37898	-2.52072	-1.91294
H	2.09461	-2.91047	-2.88765
H	2.67371	-3.32913	-1.24746
H	1.57332	-1.93743	-1.47350
C	3.97141	-1.07994	-0.76883
H	4.25331	-1.91743	-0.13387
H	4.81579	-0.41181	-0.92414
H	3.12485	-0.55151	-0.33784
C	4.67580	-2.36583	-2.71651
H	4.34701	-2.74009	-3.68327
H	5.51636	-1.68626	-2.83557
H	4.94120	-3.18940	-2.05789
C	-5.50507	-0.85346	-0.94600
F	-5.73508	-0.69139	-2.25406
F	-6.42428	-0.11479	-0.29786
F	-5.79563	-2.13473	-0.65782

TS 18aA - 19a: ΔG = -3670937.78 kJ/mol

Si	0.01703	-0.09013	-0.07138
C	1.88163	-0.05727	-0.00348
C	2.51248	1.18940	0.07781
C	2.68196	-1.19961	-0.09373
C	3.89851	1.29619	0.05727
H	1.91785	2.09422	0.15187
C	4.06746	-1.09809	-0.10482
H	2.20904	-2.16607	-0.20819
C	4.67806	0.14952	-0.03528
H	4.36795	2.27039	0.11389
H	4.67363	-1.99293	-0.17970
H	5.75823	0.22757	-0.05261
F	-0.38800	1.12256	0.94039
F	0.87606	-2.48187	-2.03804
C	-0.75135	-1.63032	0.63186

C	-0.10322	-2.34155	1.64698
C	-1.99965	-2.08646	0.19768
C	-0.67765	-3.47716	2.20498
H	0.86520	-2.01011	2.00631
C	-2.57835	-3.22190	0.75182
H	-2.52606	-1.55408	-0.58701
C	-1.91549	-3.92043	1.75427
H	-0.15924	-4.01772	2.98716
H	-3.54398	-3.56377	0.40062
H	-2.36312	-4.80798	2.18404
C	-0.72506	0.50504	-1.67785
C	-1.05162	-0.33796	-2.74538
C	-0.93301	1.88118	-1.83242
C	-1.57338	0.17887	-3.92464
H	-0.84431	-1.39800	-2.65235
C	-1.44474	2.40166	-3.01566
H	-0.69490	2.55886	-1.02040
C	-1.76826	1.54909	-4.06360
H	-1.82033	-0.48736	-4.74255
H	-1.59359	3.46955	-3.11674
H	-2.17034	1.95065	-4.98560
N	2.77246	-0.99005	-4.33202
C	1.54286	-1.55324	-4.96206
H	1.00388	-0.73761	-5.44004
H	1.84530	-2.29327	-5.69981
H	0.95391	-2.00003	-4.16094
C	3.51571	-2.08808	-3.64736
H	3.78461	-2.83338	-4.39304
H	4.40942	-1.66075	-3.19693
H	2.84663	-2.49608	-2.88892
C	2.36171	0.01270	-3.30754
H	3.25760	0.39541	-2.81963
H	1.82066	0.81432	-3.80860
H	1.72578	-0.51368	-2.60001
C	3.63161	-0.35334	-5.36320
H	3.91768	-1.11010	-6.09012
H	3.06256	0.43936	-5.84354
H	4.51198	0.05550	-4.87270

TS 18bA - 19b: ΔG = -3971561.11 kJ/mol

Si	0.02029	-0.08315	-0.08190
C	1.88535	-0.01026	-0.01456
C	2.48671	1.25375	-0.01921
C	2.71354	-1.13549	-0.02343
C	3.86980	1.39242	-0.04548
H	1.87047	2.14719	-0.01080
C	4.09658	-1.00246	-0.03875
H	2.26366	-2.11820	-0.07115

C	4.67713	0.26131	-0.05549
H	4.31546	2.37934	-0.05754
H	4.72478	-1.88505	-0.04969
H	5.75525	0.36336	-0.07679
F	-0.40074	1.15300	0.89920
F	0.87642	-2.50806	-1.87321
C	-0.72863	-1.60385	0.68430
C	-0.08773	-2.25300	1.74421
C	-1.95956	-2.10317	0.25033
C	-0.65352	-3.37054	2.34655
H	0.86833	-1.88728	2.10396
C	-2.53038	-3.21998	0.84867
H	-2.47809	-1.61904	-0.57014
C	-1.87538	-3.85690	1.89631
H	-0.14132	-3.86320	3.16373
H	-3.48346	-3.59518	0.49711
H	-2.31644	-4.72976	2.36151
C	-0.73196	0.45595	-1.69610
C	-1.01309	-0.40567	-2.75662
C	-0.99719	1.82166	-1.88569
C	-1.53775	0.05661	-3.95886
H	-0.77046	-1.45646	-2.64776
C	-1.51107	2.30167	-3.07625
H	-0.79898	2.52781	-1.08742
C	-1.78566	1.41797	-4.12145
H	-1.73822	-0.64432	-4.75690
H	-1.70927	3.35683	-3.21699
N	2.79660	-1.22247	-4.27425
C	1.56763	-1.80406	-4.88793
H	1.04334	-1.00985	-5.41602
H	1.86904	-2.58680	-5.58057
H	0.96372	-2.19949	-4.07180
C	3.51699	-2.29177	-3.52279
H	3.79108	-3.07575	-4.22582
H	4.40711	-1.85075	-3.07858
H	2.83076	-2.65488	-2.75674
C	2.38718	-0.16064	-3.30965
H	3.28305	0.23873	-2.83512
H	1.86011	0.61801	-3.85917
H	1.73992	-0.64210	-2.58007
C	3.67593	-0.65363	-5.32782
H	3.95759	-1.45132	-6.01140
H	3.12415	0.12135	-5.85497
H	4.55751	-0.23328	-4.84934
O	-2.28724	1.96885	-5.25283
C	-2.56346	1.10536	-6.34394
H	-3.31710	0.36307	-6.07280
H	-2.94328	1.73765	-7.14129

H	-1.65588	0.59745	-6.67751

TS 18cA - 19c: ΔG = -4555983.24 kJ/mol

Si	0.03893	-0.07143	-0.08045
C	1.90852	-0.01979	-0.04724
C	2.53520	1.23182	-0.02745
C	2.70522	-1.16422	-0.07658
C	3.91726	1.34137	-0.04815
H	1.94061	2.13870	-0.00195
C	4.09088	-1.06787	-0.09052
H	2.23631	-2.13655	-0.14059
C	4.68784	0.18459	-0.07995
H	4.39220	2.31483	-0.04173
H	4.69991	-1.96176	-0.11994
F	-0.35321	1.09335	0.98462
F	1.05441	-2.47700	-2.09310
C	-0.66482	-1.65912	0.57722
C	-0.06178	-2.29015	1.67042
C	-1.80939	-2.23978	0.02432
C	-0.58244	-3.46737	2.19297
H	0.82843	-1.86253	2.12028
C	-2.33336	-3.41784	0.54222
H	-2.29423	-1.77271	-0.82593
C	-1.71818	-4.03374	1.62588
H	-0.10177	-3.94426	3.03801
H	-3.21845	-3.85741	0.09959
H	-2.12309	-4.95405	2.02821
C	-0.74089	0.53468	-1.66115
C	-0.82347	-0.25405	-2.81507
C	-1.26310	1.83291	-1.70307
C	-1.41030	0.24296	-3.97220
H	-0.39329	-1.25137	-2.79323
C	-1.84786	2.33152	-2.86191
H	-1.21758	2.46386	-0.82329
C	-1.92259	1.53595	-3.99810
H	-1.46667	-0.37876	-4.85776
H	-2.24587	3.33851	-2.87592
H	-2.37936	1.92179	-4.90117
N	3.06783	-1.02836	-4.31691
C	1.86617	-1.59203	-4.99850
H	1.35164	-0.77814	-5.50547
H	2.19904	-2.33693	-5.71788
H	1.24145	-2.03620	-4.22422
C	3.76410	-2.11912	-3.57304
H	4.06031	-2.88301	-4.28903
H	4.64091	-1.69143	-3.08989
H	3.05387	-2.50460	-2.84032
C	2.61946	0.00259	-3.33761

H	3.49596	0.38427	-2.81521
H	2.11985	0.80102	-3.88342
H	1.93960	-0.49513	-2.64951
C	3.98570	-0.42269	-5.31655
H	4.30172	-1.19888	-6.00963
H	3.44969	0.36305	-5.84404
H	4.84353	-0.01021	-4.79020
C	6.17988	0.31717	-0.15389
F	6.81335	-0.84222	0.05222
F	6.65851	1.19090	0.74522
F	6.57917	0.76539	-1.35857

19a: ΔG = -3670950.71 kJ/mol

Si	-1.53523	0.00052	1.02800
C	-0.49834	-1.52380	0.78398
C	0.15978	-2.12165	1.86356
C	-0.24303	-2.00893	-0.50260
C	1.05166	-3.16876	1.66310
H	-0.01671	-1.76250	2.87151
C	0.65387	-3.04882	-0.70883
H	-0.73586	-1.55972	-1.35953
C	1.30311	-3.62882	0.37554
H	1.55435	-3.62107	2.50876
H	0.84826	-3.40542	-1.71272
H	2.00432	-4.43870	0.21699
F	-1.98413	0.00090	2.57802
F	1.66602	-0.00021	-3.04367
C	-3.04587	0.00071	-0.05103
C	-3.64603	-1.20098	-0.44216
C	-3.64624	1.20253	-0.44140
C	-4.81037	-1.20323	-1.19913
H	-3.19873	-2.14677	-0.15562
C	-4.81055	1.20506	-1.19840
H	-3.19910	2.14822	-0.15426
C	-5.39248	0.00098	-1.57732
H	-5.26276	-2.14138	-1.49453
H	-5.26310	2.14332	-1.49323
H	-6.29928	0.00109	-2.16915
C	-0.49759	1.52427	0.78358
C	-0.24207	2.00906	-0.50308
C	0.16093	2.12191	1.86303
C	0.65543	3.04840	-0.70951
H	-0.73526	1.56004	-1.35990
C	1.05340	3.16846	1.66239
H	-0.01571	1.76300	2.87105
C	1.30507	3.62817	0.37474
H	0.84996	3.40476	-1.71346

H	1.55639	3.62061	2.50795
H	2.00676	4.43761	0.21603
N	3.84345	-0.00079	-0.56281
C	4.01339	1.21542	-1.40986
H	3.87326	2.08977	-0.77709
H	5.01863	1.20449	-1.82562
H	3.25490	1.16494	-2.19095
C	4.01327	-1.21644	-1.41072
H	5.01848	-1.20528	-1.82653
H	3.87314	-2.09121	-0.77853
H	3.25472	-1.16537	-2.19171
C	2.46171	-0.00094	0.00024
H	2.33843	-0.89778	0.60585
H	2.33865	0.89514	0.60701
H	1.77805	-0.00029	-0.84703
C	4.84197	-0.00124	0.53784
H	5.83815	-0.00121	0.10174
H	4.69027	0.89183	1.13985
H	4.69009	-0.89465	1.13929

19b: ΔG = -3971573.76 kJ/mol

Si	1.77340	-0.01625	1.02242
C	1.20567	1.70542	0.60369
C	0.79506	2.59308	1.60286
C	1.05123	2.08957	-0.73226
C	0.23920	3.82515	1.27759
H	0.90104	2.31954	2.64682
C	0.48992	3.31616	-1.06243
H	1.35870	1.41851	-1.52885
C	0.08094	4.18433	-0.05597
H	-0.07551	4.50124	2.06270
H	0.36834	3.59370	-2.10213
H	-0.35978	5.14002	-0.31114
F	2.20606	0.02822	2.57688
F	-1.60365	0.48980	-3.00999
C	3.22820	-0.53022	-0.01191
C	4.15670	0.41680	-0.45804
C	3.45121	-1.87618	-0.32110
C	5.27635	0.03158	-1.18376
H	4.00358	1.46817	-0.23854
C	4.56869	-2.26610	-1.04805
H	2.74365	-2.62973	0.00753
C	5.48218	-1.31121	-1.47878
H	5.98615	0.77642	-1.52070
H	4.72653	-3.31194	-1.27933
H	6.35372	-1.61318	-2.04610
C	0.37046	-1.21873	0.89068
C	-0.04612	-1.67272	-0.36040

C	-0.40261	-1.58024	2.00337
C	-1.19152	-2.44323	-0.52195
H	0.52032	-1.41128	-1.24944
C	-1.54146	-2.35168	1.86664
H	-0.11592	-1.24687	2.99452
C	-1.94664	-2.78100	0.59965
H	-1.48274	-2.76500	-1.51164
H	-2.13840	-2.62957	2.72619
N	-3.48204	1.29628	-0.40081
C	-4.02881	0.07499	-1.05970
H	-4.05235	-0.72621	-0.32286
H	-5.03521	0.29600	-1.40949
H	-3.36158	-0.16518	-1.88734
C	-3.43246	2.40762	-1.39507
H	-4.44658	2.60718	-1.73453
H	-3.01795	3.28425	-0.90075
H	-2.79372	2.07157	-2.21193
C	-2.09382	1.00799	0.06277
H	-1.69491	1.90586	0.53249
H	-2.13435	0.18589	0.77582
H	-1.51663	0.73598	-0.81931
C	-4.33544	1.68015	0.75399
H	-5.33893	1.88629	0.38889
H	-4.35013	0.85333	1.46061
H	-3.90882	2.56756	1.21627
O	-3.08072	-3.51601	0.55431
C	-3.52189	-3.98177	-0.71270
H	-2.77513	-4.63586	-1.16718
H	-4.43483	-4.54001	-0.52716
H	-3.73137	-3.14567	-1.38341

19c: ΔG = -4555997.05 kJ/mol

Si	-1.96083	-0.41570	1.04123
C	-0.28694	-1.17577	0.71827
C	0.61254	-1.39590	1.76708
C	0.14412	-1.40066	-0.59028
C	1.90886	-1.81608	1.51479
H	0.30427	-1.22883	2.79237
C	1.44373	-1.81249	-0.85698
H	-0.53089	-1.23544	-1.42379
C	2.31914	-2.01231	0.20040
H	2.60095	-1.98057	2.33192
H	1.76978	-1.96795	-1.87669
F	-2.25340	-0.65372	2.60808
F	0.75846	1.36860	-3.07235
C	-3.28116	-1.20855	0.00939
C	-3.17416	-2.54738	-0.38341
C	-4.42539	-0.49418	-0.36221

C	-4.18191	-3.15671	-1.11936
H	-2.29362	-3.12152	-0.11521
C	-5.43538	-1.09999	-1.09816
H	-4.52902	0.54724	-0.07674
C	-5.31291	-2.43194	-1.47674
H	-4.08474	-4.19318	-1.41664
H	-6.31489	-0.53453	-1.37893
H	-6.09836	-2.90469	-2.05311
C	-1.87531	1.42316	0.79582
C	-1.91923	1.96691	-0.49233
C	-1.63061	2.27914	1.87398
C	-1.71043	3.32378	-0.70066
H	-2.10265	1.32363	-1.34765
C	-1.43041	3.63970	1.67090
H	-1.58931	1.88216	2.88214
C	-1.46402	4.16069	0.38250
H	-1.73626	3.72798	-1.70499
H	-1.24205	4.29101	2.51507
H	-1.29875	5.21885	0.22205
N	2.60833	2.44566	-0.56483
C	2.09190	3.59743	-1.36009
H	1.49893	4.22644	-0.69899
H	2.94298	4.15207	-1.74942
H	1.48388	3.17958	-2.16244
C	3.39641	1.54912	-1.45992
H	4.22705	2.11985	-1.86941
H	3.76702	0.71701	-0.86438
H	2.71746	1.20876	-2.24191
C	1.44761	1.68064	-0.02454
H	1.83154	0.83026	0.53745
H	0.87251	2.34016	0.62402
H	0.85681	1.35930	-0.88112
C	3.46049	2.93400	0.55070
H	4.29956	3.48321	0.12963
H	2.85920	3.58278	1.18358
H	3.81377	2.07396	1.11558
C	3.73533	-2.44648	-0.04317
F	4.03781	-2.52294	-1.34218
F	3.98809	-3.65359	0.48909
F	4.61721	-1.59920	0.51460

6. References

1. P. Migowski, G. Machado, S. R. Texeira, M. C. Alves, J. Morais, A. Traverse, J. Dupont, *Phys. Chem. Chem. Phys.*, **2007**, *9*, 4814-4821.
2. J. J. Tufariello, H. Meckler, K.Pushpananda, A. Senaratne, *Tetrahedron* **1985**, *41*, 3447-3453.
3. J. Cason, R. J. Fessenden, *J. Org. Chem.* **1957**, *22*, 1326-1332.
4. M. Yus, R. P. Herrera, A. Guijarro, *Chem. - Eur. J.* **2002**, *8*, 2574-2584.
5. M. A. Tehfe, S. Schweizer, A. C. Chany, C. Yascco, J. L. Clement, D. Gigmes, F. Morlet-Savary, J. P. Fouassier, M. Neuburger, T. Tschamber, N. Blanchard, J. Lalevee, *Chem. - Eur. J.* **2014**, *20*, 5054-5063.
6. A. Karanfil, M. Eskici, *Synt. Commun.* **2017**, *47*, 2342-2351.
7. M. Donnard, T. Guérin, G. Hanquet, F. R. Leroux, A. Panossian, J. Saiter, *Eur. J. Org. Chem.* **2021**, *2021*, 3139-3147.
8. A. Tanaka, H. Togo, *Synlett*, **2009**, *20*, 3360-3364.
9. D. A. Powell, G. C. Fu, *J. Am. Chem. Soc.* **2004**, *126*, 7788-7789.
10. P. Švec, P. Novák, M. Nádvorník, Z. Padělková, I. Císařová, L. Kolářová, A. Růžička and J. Holeček, *J Fluorine Chem.*, 2007, **128**, 1390-1395.
11. Gaussian 16, Revision C.01, M. J. Frisch, G. W. Trucks, H. B. Schlegel, G. E. Scuseria, M. A. Robb, J. R. Cheeseman, G. Scalmani, V. Barone, G. A. Petersson, H. Nakatsuji, X. Li, M. Caricato, A. V. Marenich, J. Bloino, B. G. Janesko, R. Gomperts, B. Mennucci, H. P. Hratchian, J. V. Ortiz, A. F. Izmaylov, J. L. Sonnenberg, D. Williams-Young, F. Ding, F. Lipparini, F. Egidi, J. Goings, B. Peng, A. Petrone, T. Henderson, D. Ranasinghe, V. G. Zakrzewski, J. Gao, N. Rega, G. Zheng, W. Liang, M. Hada, M. Ehara, K. Toyota, R. Fukuda, J. Hasegawa, M. Ishida, T. Nakajima, Y. Honda, O. Kitao, H. Nakai, T. Vreven, K. Throssell, J. A. Montgomery Jr., J. E. Peralta, F. Ogliaro, M. J. Bearpark, J. J. Heyd, E. N. Brothers, K. N. Kudin, V. N. Staroverov, T. A. Keith, R. Kobayashi, J. Normand, K. Raghavachari, A. P. Rendell, J. C. Burant, S. S. Iyengar, J. Tomasi, M. Cossi, J. M. Millam, M. Klene, C. Adamo, R. Cammi, J. W. Ochterski, R. L. Martin, K. Morokuma, O. Farkas, J. B. Foresman and D. J. Fox, *Gaussian, Inc.*, Wallingford CT, 2016.
12. Y. Zhao and D. G. Truhlar, *Theor. Chem. Acc.*, 2007, **120**, 215-241.
13. O. Vahtras, J. Almlöf and M. W. Feyereisen, *Chem. Phys. Lett.*, 1993, **213**, 514-518.
14. D. Rappoport and F. Furche, *J. Chem. Phys.*, 2010, **133**, 134105.
15. F. Weigend and R. Ahlrichs, *Phys. Chem. Chem. Phys.*, 2005, **7**, 3297-3305.
16. A. V. Marenich, C. J. Cramer and D. G. Truhlar, *J. Phys. Chem. B*, 2009, **113**, 6378-6396.
17. F. Neese, F. Wennmohs, U. Becker and C. Riplinger, *J. Chem. Phys.*, 2020, **152**, 224108.
18. F. Neese, F. Wennmohs, A. Hansen and U. Becker, *Chem. Phys.*, 2009, **356**, 98-109.
19. J. Zheng, X. Xu and D. Truhlar, *Theoretical Chemistry Accounts*, 2011, **128**, 295-305.
20. F. Weigend, *Phys. Chem. Chem. Phys.*, 2006, **8**, 1057-1065.
21. V. Barone and M. Cossi, *J. Phys. Chem. A*, 1998, **102**, 1995-2001.
22. S. Grimme, S. Ehrlich and L. Goerigk, *J. Comp. Chem.*, 2011, **32**, 1456-1465.
23. APEX4, SAINT. Bruker AXS Inc., Madison, Wisconsin, USA, 2019.
24. A. Altomare, G. Cascarano, G. Giacovazzo, A. Guagliardi, M. C. Burla, G. Polidori, M. Camalli, *J. Appl. Cryst.* **1994**, *27*, 435.
25. P. W. Betteridge, J. R. Carruthers, R. I. Cooper, K. Prout, D. J. Watkin, *J. Appl. Cryst.* **2003**, *36*, 1487
